# Non-natural 3-Arylmorpholino-β-amino Acid as a PPII Helix Inducer

**DOI:** 10.1021/acs.orglett.0c02331

**Published:** 2020-07-30

**Authors:** Francesco Vaghi, Raffaella Bucci, Francesca Clerici, Alessandro Contini, M. Luisa Gelmi

**Affiliations:** DISFARM-Sez. Chimica Generale e Organica “A. Marchesini”, Università degli Studi di Milano, via Venezian 21, 20133 Milano, Italy

## Abstract

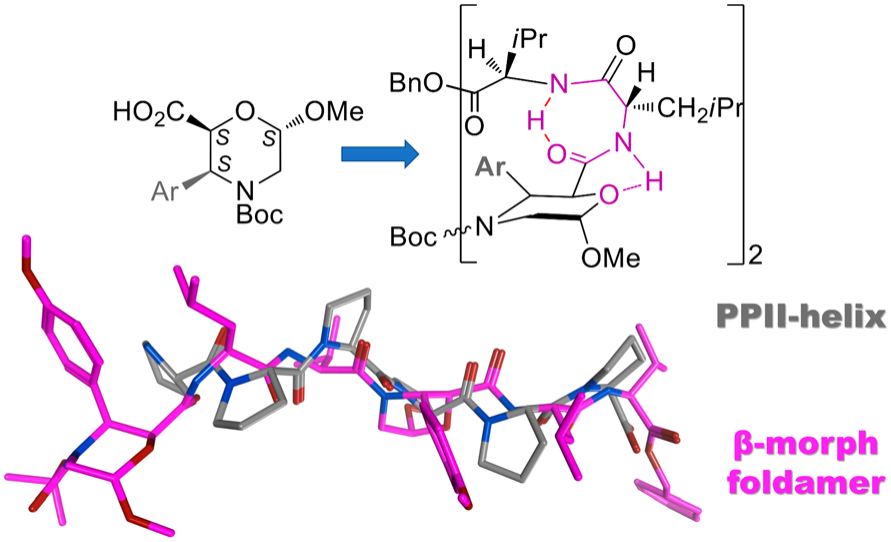

A new non-natural β-amino acid, named 3-Ar-β-Morph, was designed and synthesized via a regio- and diastereoselective Pd-catalyzed C(sp^3^)H-arylation of the corresponding 2*S*,6*S*-(6-methoxymorpholin-2-yl)carboxylic acid, readily available from glucose. According to the computational prevision and confirmed by IR and NMR data, the insertion of 3-Ar-β-Morph in a model foldamer represents a way to stabilize a PPII-like helix through the presence of two γ-turns, secondary structure motifs induced by the morpholine ring, and the *trans*-tertiary amide bond.

Peptides are bioactive molecules designed by Nature to absolve numerous functions in several biological processes. Their ability to assume specific conformations and to organize in three-dimensional folded structures is driven by the encoded amino acid sequences.^1,2^ Among the ubiquitous secondary structures in folded proteins (α-helices, 3,10-helices, and β-sheet), the less abundant but still frequently occurring regular structure is the polyproline II helix (PPII).^3^ This helix is characterized by ϕ and ψ torsional angles of about −75° and 150°, respectively, and, contrary to PPI, is left-handed with *trans* peptide bonds.^4^ Its importance in biological systems emerged in recent years: from the transcription to the cell motility and from the bacterial and viral pathogenesis to amyloidogenic proteins.^5^ Moreover, it is also at the basis of the collagen triple helix, formed by three PPIIs that coil into each other thanks to intermolecular hydrogen bonds.^6^ PPII is also important in unfolded proteins, being considered as a transition structure between a helix and a random coil.^3,7^ With these premises, it is clear how the synthesis of PPII structure-mimics is of relevance. Some examples of inhibitors/modulators of proline-rich-mediated protein–protein interactions are present,^8−10^ as well as some synthetic collagen model peptides containing modified prolines.^11−14^

Small molecules or noncoded amino acids (AAs)^15^ are commonly useful as inducers of a particular secondary structure. The derived peptidomimetics^16−18^ are characterized by similar features of the target peptide^19^ but with increased proteolytic stability.^20−22^

Despite the large number of protocols involving β-AAs for locking peptides into helixes and β-strand conformations, their use for generating mimics of PPII structures is absent in the literature.^23^

Recently, we reported on the synthesis of a morpholino β-AA, named β-Morph **1**, that was inserted in peptide sequences (*n* = 1,2; R = H; Figure 1).^24^

**Figure 1:**
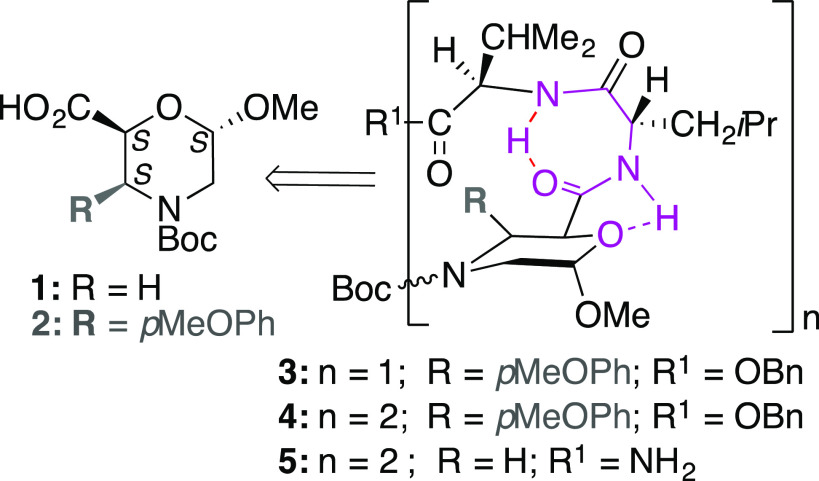
β-Morpholino-containing peptides.

Due to the formation of a strong H-bond between the oxygen of the morpholino ring and NH of amino acid *i*+1, γ-turn/s stabilized by a H-bond between C=O and NH at positions *i* and *i*+2 can be formed. Interestingly, the hexapeptide **5** (Figure 1) also showed an equilibrium between α- and PP-helixes. The shift between the two geometries, facilitated by the γ-turn, was attributed to the rotation of the tertiary amide bond.^24^ In order to block this rotation, we evaluated the possibility of introducing a bulky group in the α-position of the tertiary amide bond on the morpholino ring, thus stabilizing one of the two conformations.

Aiming to obtain a PPII structure, a preliminary computational study was performed evaluating the use of a 3-aryl-substituted analogue of **1** in both configurations. On the basis of computational predictions, the (2*S*,3*S*,6*S*)-6-methoxy-3-(4-methoxyphenyl)morpholine-2-carboxylic acid scaffold, named 3-Ar-β-Morph **2** (Figure 1), was prepared by a Pd-catalyzed regio- and diastereoselective C(sp^3^)H-arylation. Its insertion in the same sequence reported in Figure 1 (*n* = 2, R = *p*MeOPh) gave a PPII-like conformation, as confirmed by IR/NMR studies, proving that 3-Ar-β-Morph is the first non-natural β-AA that can be used to stabilize PPII in peptides.

## Computational Study

**Figure 2:**
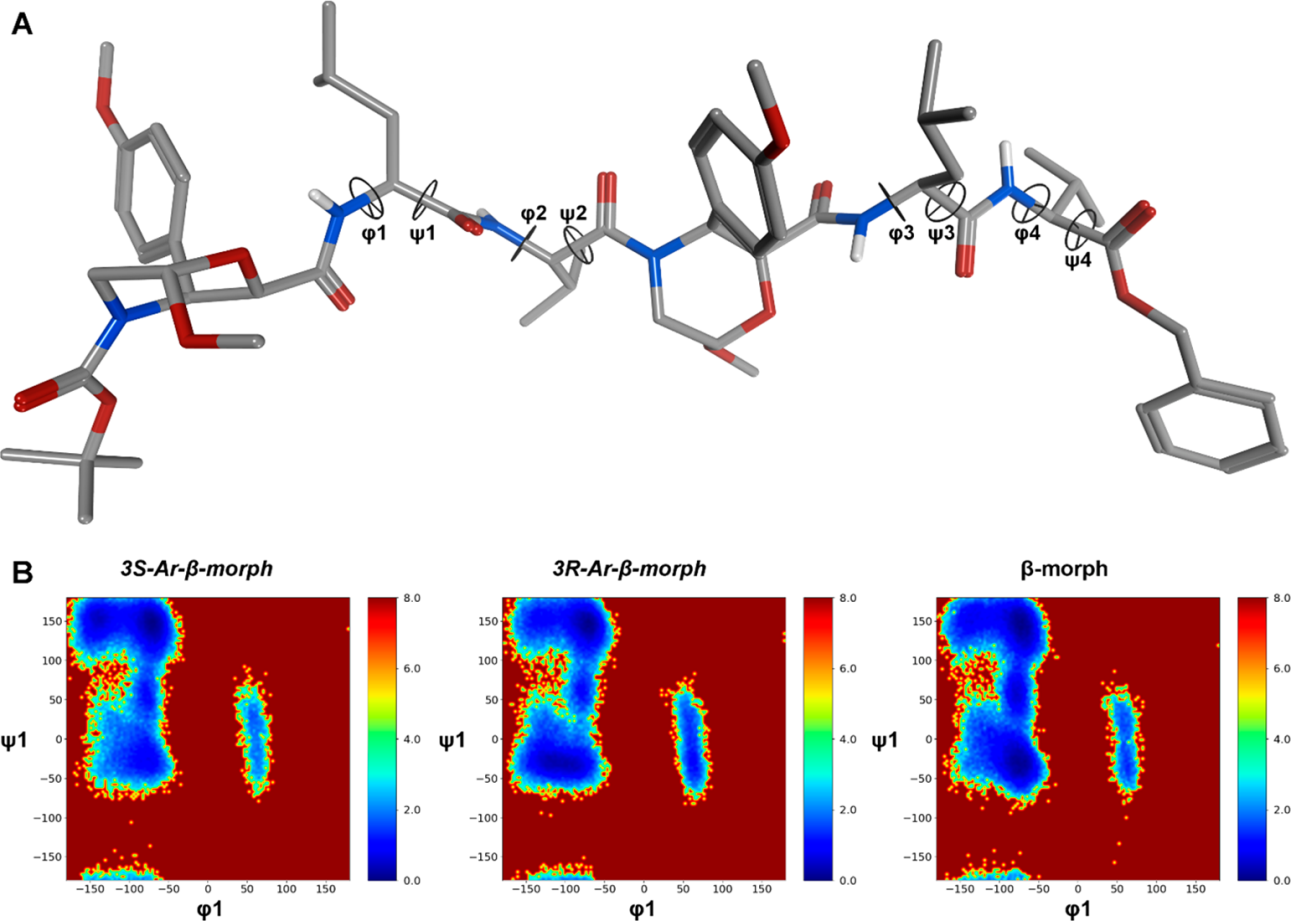
(A) Representative geometry of the most populated cluster c0 for (3*S*)-**4**. (B) Heatmaps describing the relative free energy (kcal/mol) associated with different values for the φ1/ψ1 dihedral pair for peptides (3*S*)-**4**, (3*R*)-**4**, and **5**,^24^ containing 3*S*-Ar-β-Morph, 3*R*-Ar-β-Morph, and β-Morph, respectively. The geometrical analysis was conducted on the 1000–1500 ns section of the unbiased H-REMD replica. Heatmaps for all the considered dihedral pairs are reported in Figure S1.

As reported,^24^ H-REMD simulations described the preferred conformations of tri- and hexapeptides containing β-Morph **1** successfully. Thus, we performed a prospective computational study to evaluate the role of the *p*-methoxyphenyl group at C-3 and of the stereochemical configuration at the same carbon in ruling the conformational preferences of *N*-Boc-[(*S*/*R*)-Ar-β-Morph-Leu-Val]_2_-OBn peptide, here referred to as (3*S*)- and (3*R*)-**4**. H-REMD simulations were performed in explicit MeCN solvent (12 replica of 1.5 μs each, for a total of 18 μs of simulations). The unbiased replica was then analyzed by clustering analysis to obtain a description of the different conformations, and relative weights, accessible by the two peptides. Additionally, the backbone φ and ψ dihedrals were analyzed on the same trajectory. Heatmaps representing the probability of occurrence of φ/ψ dihedral pairs for each residue were produced to evidence any difference between (3*S*)- and (3*R*)-**4**, in comparison with the peptide **5** containing **1** (Figure 1).^24^ Results are reported in Tables 1 and S1 and in Figures 2, S1, and S2. As expected, the insertion of the aryl group at C-3 induced a significant change in the conformational preferences of peptides (3*S*)-**4** and (3*R*)-**4** (Figures 2 and S1), compared to **5**.

**Table 1:** Representative Results for the Geometrical^a^ and Cluster Analyses of the H-REMD Trajectory for the Unbiased Replica

	(3*S*)-**4**	(3*R*)-**4**
φ1	–54.8 ± 43.4	–111.0 ± 17.0
ψ1	135.8 ± 28.0	–33.0 ± 9.7
φ2	–75.8 ± 27.7	–128.5 ± 8.9
ψ2	152.4 ± 21.1	82.7 ± 11.1
φ3	–86.9 ± 25.6	–72.0 ± 11.0
ψ3	147.0 ± 49.4	–6.9 ± 18.2
φ4	–75.4 ± 26.5	–80.8 ± 18.6
ψ4	127.9 ± 29.1	122.8 ± 12.1
c0 pop (%)^b^	67.3	29.2

a Dihedral values taken from the most representative conformation of the main cluster c0 (intervals are the mean deviations of the whole c0 population from the centroid).

b Population is reported for c0 only (see Table TS1 for all cluster populations).

However, a different behavior was observed depending on the stereochemistry at C-3. The population of the main conformational cluster is different for (3*S*)-**4** and (3*R*)-**4** (67.3% and 29.2%, respectively; Tables 1 and TS1) suggesting that the former is conformationally more stable. Moreover, for (3*S*)-**4**, the average φ and ψ dihedrals of the main cluster (c0) population are within the typical PPII-helices range (about −75° and 150°, respectively; Table 1 and Figure 2A). Conversely, a disordered conformation is predicted for (3*R*)-**4**, where the average φ and ψ dihedrals of c0 do not match any well-defined secondary structure (Table 1 and Figure S2). The different behavior of (3*S*)-**4**, (3*R*)-**4**, and **5** is also well described by the heatmaps (Figures 2B and S1) obtained from the analysis of the last 500 ns of the H-REMD trajectory, representing the conformational free energy surfaces derived from the Boltzmann distributions of selected dihedral pairs. Indeed, a deep well at about φ1 = −80° and ψ1 = 150° can be observed for (3*S*)-**4**. Conversely, for (3*R*)-**4**, an additional and rather wide low energy region is observed at about φ1 = −100° and ψ1 = −50°. Furthermore, the region corresponding to the left-handed helix (30° ≤ φ ≤ 130° and −50° ≤ ψ ≤ 100°)^25^ also is energetically more accessible, compared to (3*S*)-**4**. In conclusion, β-Morph **1** seems to favor both PP- and α-helix geometries, as well as the transition region between them represented by the inverse γ-turn region (φ ≈ −80° and ψ ≈ 70°). Conversely, (3*R*)-Ar-β-Morph still induces α- and PP-helixes, but with a less favored inverse γ-turn region. Moreover, the left-handed α-helix appears to be more accessible with respect to foldamers containing **1** or **2**. Interestingly, this latter seems to be able to stabilize the PP-region mainly. We also investigated if **4** might replace polyproline in a biological complex. Using the structure of the profilin–poly-l-proline complex^26^ as a reference, we performed MD simulations and binding energy calculations^27^ on both the reference and the model where (*3S*)-**4** replaced the polyproline chain. This latter remained stable during 100 ns of simulation and computed binding energy resulted lower than that of the reference (Figure S3 and Table TS2).

## Synthesis of the New Scaffold **2**

**Scheme 1:**
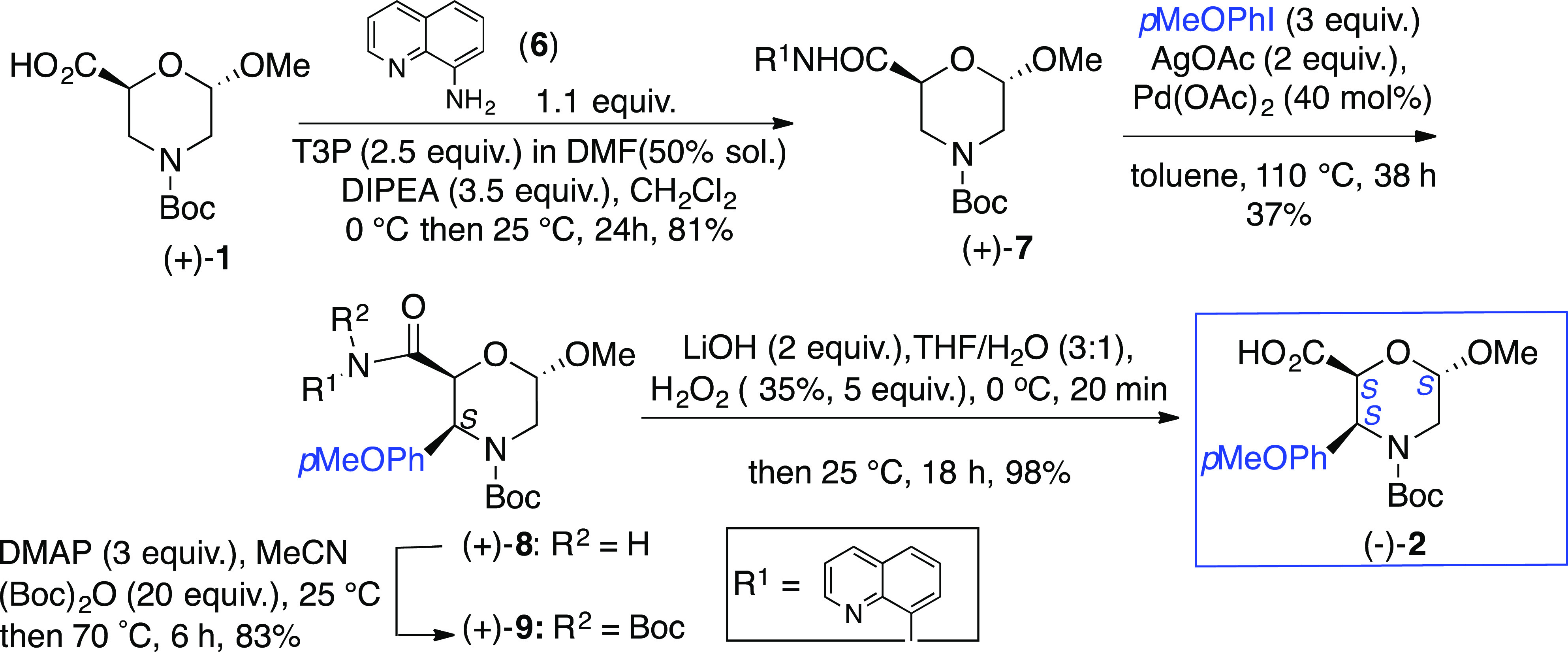
Synthesis of 3-Ar-β-Morph (−)-**2**

The enantiopure β-Morph (+)-**1** (Figure 1, R = H) was prepared starting from glucose.^24^ First, **1** was transformed into amide **7** by reaction with 8-aminoquinoline (**6**). As reported,^28^ a regio- and diastereoselective Pd-catalyzed C(sp^3^)H-arylation could be mediated by the Pd-coordinating nitrogen of quinoline ring. Amide **7** (43%) was first prepared by using a reported protocol.^28^ On the other hand, a strong improvement in the yield was achieved by activation of **1** with propylphosphonic anhydride [T3P, 2.5 equiv; 50% DMF solution in CH_2_Cl_2_, DMAP (3.5 equiv), 0 °C, 1 h; then 24 h at 25 °C] followed by reaction with **6** (1.1 equiv, 25 °C, 24 h) gaving (+)-**7** in 81% yield (Scheme 1).

The arylation at C-3 for a similar compound of **7** (10% yield) is reported [Pd(OAc)_2_ (0.1 equiv), AcOAg (2 equiv), MeOPhI (3 equiv) toluene, reflux, 38 h)].^28^ The same protocol, starting from **7** and *p*-iodoanisole, gave analogous yields of **8**. Several attempts were performed to optimize this procedure. While the use of toluene was found to be crucial (other solvents inhibit the reaction), incrementing the amount of Pd(OAc)_2_—from 0.2 to 0.4 equiv—increased the yield up to 37% (63% recovery starting material). Unfortunately, no improvement was observed by changing the catalyst (Cu(OAc)_2_, Cu(TFA)_2_, Pd(TFA)_2_, PdCl_2_) or the oxidant (AgTFA instead of AcOAg). The reaction is regio- and diastereoselective, affording only compound (+)-**8** having the aryl moiety in *cis* relationship with the carbonyl group.

To synthesize the deprotected carboxylic acid **2**, *N*-Boc amide (+)-**9** was prepared first [(Boc)_2_O (20 equiv), DMAP (3 equiv), MeCN, 70 °C, 6 h; 83%]. Its hydrolysis [LiOH·H_2_O (2 equiv)/H_2_O_2_ (35%, 5 equiv), THF/H_2_O (3:1), 25 °C, 18 h] gave (−)-**2** (98%).

## Foldamer Synthesis

**Scheme 2:**
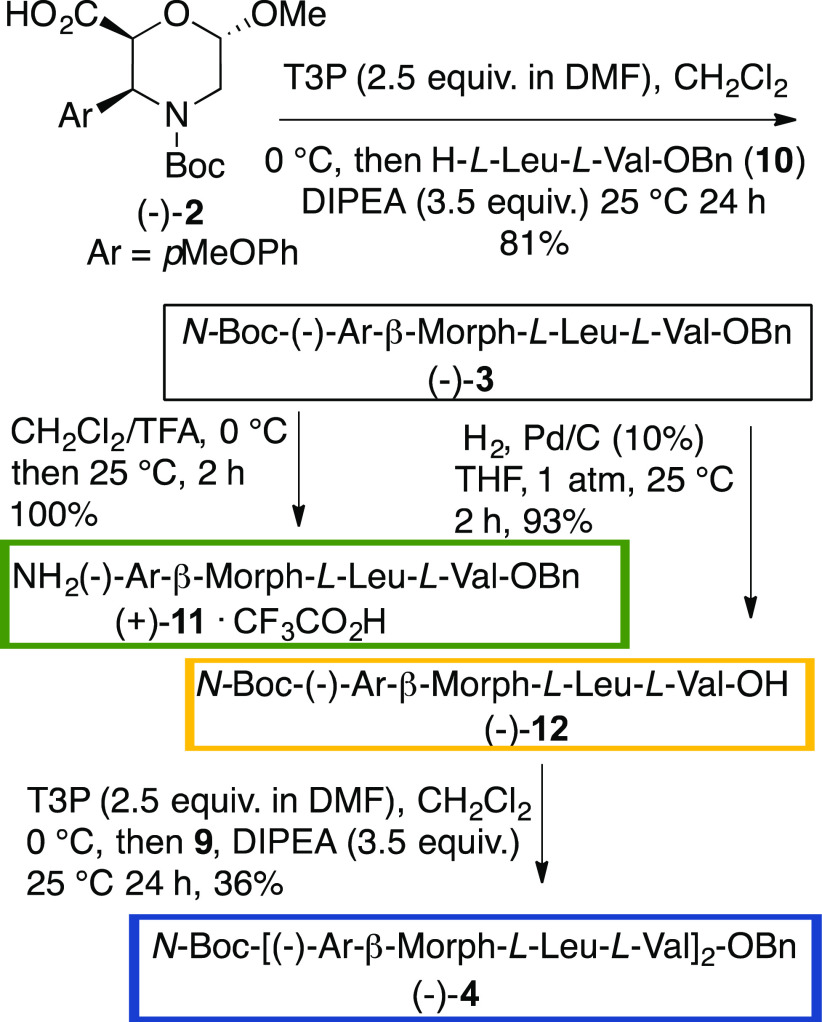
Synthesis of Tripeptide (−)-**3** and Hexapeptide (−)-**4**

Foldamer syntheses (Scheme 2) were optimized by using different coupling agents. Starting from (−)-**2** and dipeptide **10**, T3P [(DMF/DMAP solution) in CH_2_Cl_2_ (0 °C, 1 h then 24 h at 25 °C) is the most efficient coupling agent, giving tripeptide (−)-**3** in 81% yield. Tripeptide was selectively deprotected (TFA, CH_2_Cl_2_, 25 °C, 2 h), giving (+)-**11** (quantitative yield). A reductive debenzylation of **3** (H_2_, Pd/C, THF, 1 atm., 25 °C, 2 h) provided (−)-**12** (93%). The coupling of **11** with **12** gave low yield of hexapeptide **4** (17 and 8%, respectively) when using HOBt/EDC or HOBt [(1.1 equiv)/EtCN-oxime (1.1 equiv)/DIPEA (2.1 equiv)]. T3P was the best coupling agent, improving the yield of (−)-**4** (36%). The steric demanding coupling reaction probably affected the high reaction yields.

## IR Characterization

FTIR analysis was performed on a solid sample of (−)-**4** (Figure S8). The PP-conformation is confirmed by the presence of a peak around 1640 cm^–1^, corresponding to the PP-characteristic C=O stretching frequency (amide I).^29^

## NMR Characterization

The stereochemistry of (−)-**2** at C-3 was assigned based on *J* values and NOESY experiments. The *trans* disposition of H-2/H-3 is excluded by the *J* value (5.1 Hz), and a distorted morpholino chair is suggested (*J*_5ax,6_ = 8.9 Hz, *J*_5eq,6_ = 5.4 Hz). NOEs were detected between H-2/Boc (w) and aryl group with H-2, H-3, and H-5_ax_, indicating the pseudoaxial disposition of the aryl moiety *cis* with respect to the carboxylic function (Figures S4, Table TS3).

Tripeptides (−)-**3** and hexapeptide (−)-**4** were characterized by NMR (^1^H, ^13^C, COSY, TOCSY, HMBC, HMQC, NOESY; 600 MHz) in CD_3_CN solution, and δ values of morpholino and α-AA protons were unequivocally assigned (Tables S4 and S5). Tripeptide **3** showed several similarities^24^ with the tripeptide of Figure 1 (*n* = 1, R = H, R^1^ = Bn): a γ-turn is present at *C*-terminus. As reported in Figure S5, weak NOEs are those between NH_Leu_ with OMe_Morph_, H-6, and H-2, indicating its orientation toward the oxygen region of the ring. The formation of the γ-turn is supported by the spatial proximity of NH_Val_ with the leucine moiety. Furthermore, low δΔ/Δ*T* values (273–323 K; Figure S6) for NH_Val_ (−1.8 ppb K^–1^) and NH_Leu_ (−2.2 ppb K^–1^) were detected. Accordingly, a H-bond between NH_Val_ and C=O_Morph_ is suggested, driven by a second strong H-bond between NH_Leu_ and the oxygen of the ring.

**Figure 3:**
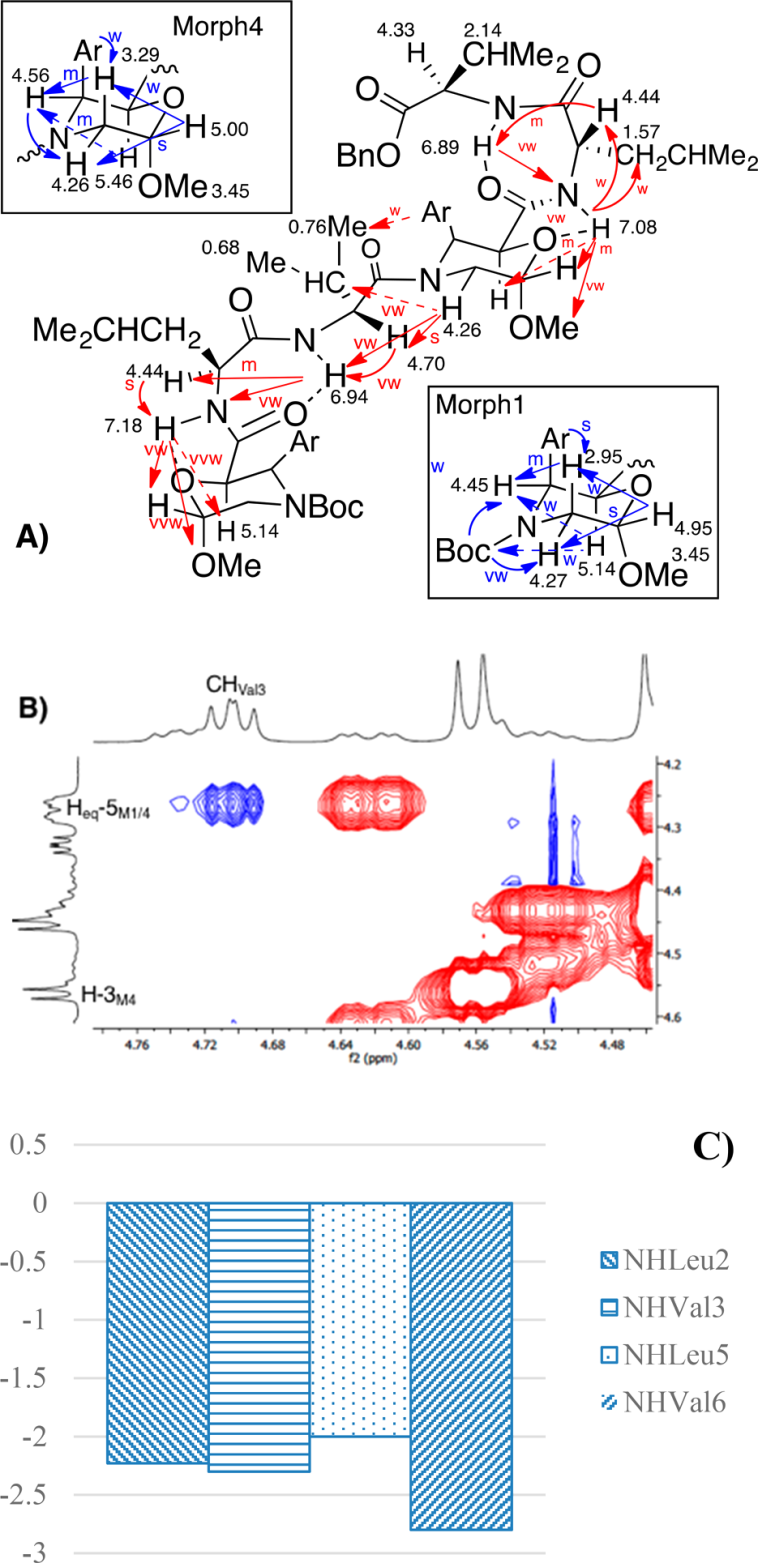
NMR data for hexapeptide (−)-**4** (CD_3_CN, mM, 600 MHz): (A) NOEs of morpholino ring protons (blue arrows) and between the different AAs (red arrows) and H-bonds (dotted lines). (B) Zoom of Val_3_/Morph_4_ region. (C) Δδ/Δ*T* NH values (273–333 K).

Interestingly, the NMR analysis of (−)-**4** showed the presence of a main conformer (80:20, ^1^H NMR data). Very low δΔ/Δ*T* values (273–333 K) for all NHs ranging from −2.8 to −2 ppbK^–1^ (Figure 3C) were found for the main isomer, supporting a strong H-bond network, as indicated for **3**. NOEs of compound (−)-**4** are shown in Figures 3A and S7. Similar spatial proximities between NH_Leu2_ and NH_Leu5_ with the acetal region of the corresponding morpholine ring at positions 1 and 4 are present, supporting the formation of two γ-turns. Spatial proximities between CH_Val3_ and protons of morpholine-4 are diagnostic for the prediction of the main conformer. NOEs were detected between CH_Val3_ and H_eq_-5_Morph4_ but not with H-3_Morph4_ (Figure 3B), thus indicating the orientation of C=O toward the aryl region. As a result, the *E*-conformer is suggested for the tertiary amide bond.

In conclusion, starting from our previous knowledge indicating that scaffold **1** is able to generate a mixture of α- and PPII-like helixes, we designed the new scaffold (−)-**2**. We demonstrated by computational, IR and NMR data that **2** represents the first β-AA able to induce a PPII helix when inserted in a model foldamer due to the presence of a hindered aryl-substituent at C-3 that blocks the rotation of the tertiary amide bond favoring the *trans*-conformation.

## Supporting Information Available

Computational details, synthetic protocols, and IR, ^1^H NMR, ^13^C NMR spectra for compounds **2**–**4**, **7**–**9**, **11**, and **12** (PDF)
