# Supplementary material for: Non-natural 3-Arylmorpholino-β-amino Acid as a PPII Helix Inducer
Source: Org Lett. 2020 Jul 30;22(15):6197–202. doi: 10.1021/acs.orglett.0c02331 (PMC8009597; doi:10.1021/acs.orglett.0c02331)
Supplement: Supplementary file 1 — ol0c02331_si_001.pdf [file ol0c02331_si_001.pdf]

# Supporting Information

## Non-Natural 3-Aryl-morpholino-β-amino Acid as PPII helix inducer

Francesco Vaghi,‡ Raffaella Bucci,‡ Francesca Clerici, Alessandro Contini,* and M. Luisa Gelmi*

DISFARM-Sez. Chimica Generale e Organica "A. Marchesini", Università degli Studi di Milano, via Venezian 21, 20133 Milano, Italy

1. Computational Studies ........ 2
Simulation of (3*S*)-4 in a biological complex ........ 3
Computational methods ........ 4
Coordinates of representative geometries ........ 6
2. Synthesis of compounds **2-4,7-9,11,12** ........ 13
3. $^1$H NMR and $^{13}$C NMR ........ 24
Compound **7** ........ 24
Compound **8** ........ 26
Compound **9** ........ 28
Compound **2** ........ 30
Compound **3** ........ 32
Compound **11** ........ 34
Compound **12** ........ 36
Compound **4** ........ 38
4. IR Spectrum of Peptide **4** ........ 40
References ........ 41

# 1. Computational Studies

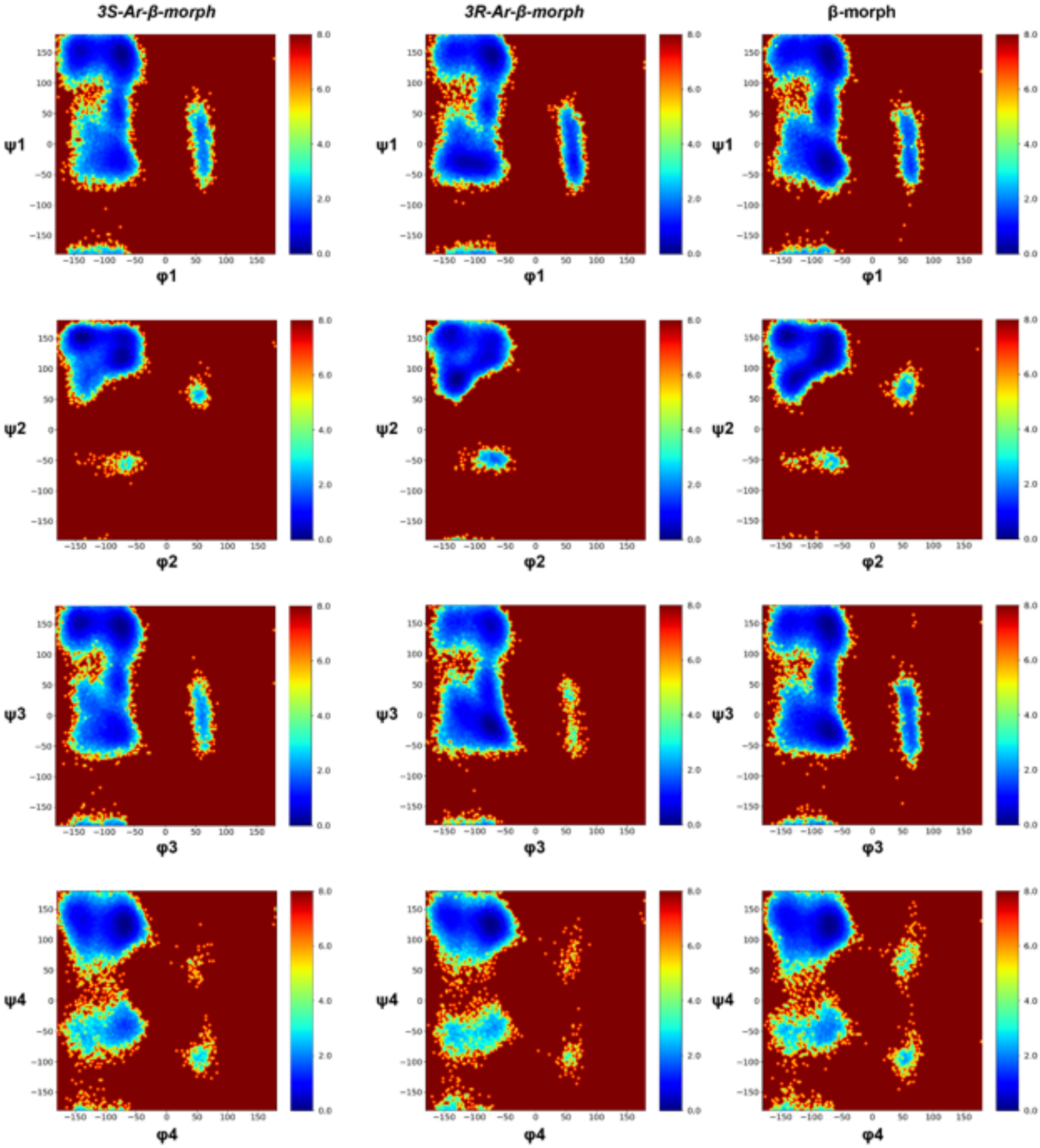


**Figure S1.** Heatmaps describing the relative free energy, in kcal/mol, associated to different values for the φ1/ψ1, φ2/ψ2, φ3/ψ3 and φ4/ψ4 dihedral pair for peptides (3*S*)-**4**, (3*R*)-**4** and **5**,[1] containing 3*S*-Ar-β-Morph, 3*R*-Ar-β-Morph and β-Morph, respectively.

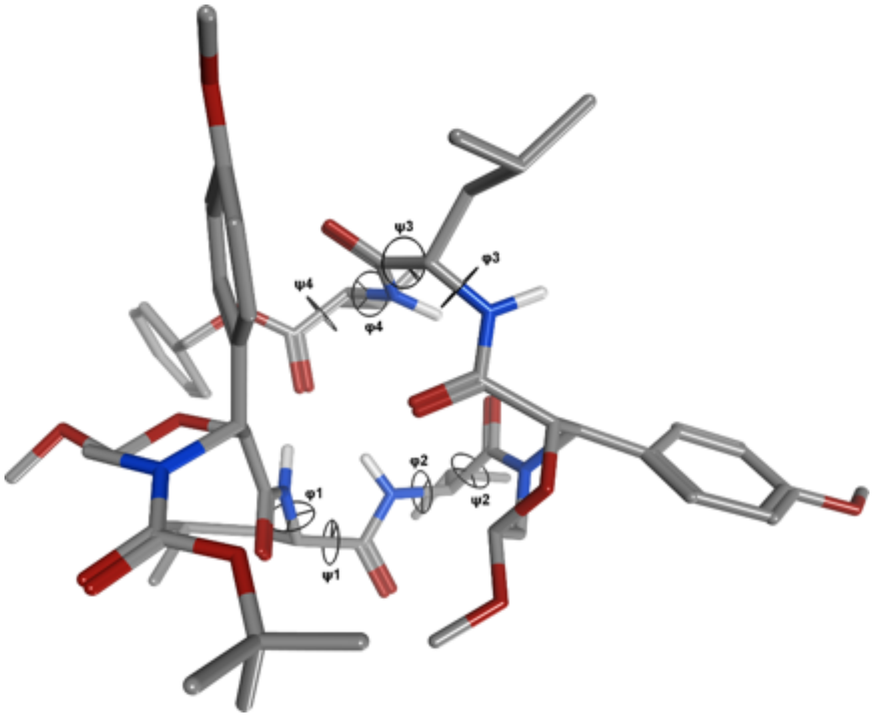


**Figure S2**. Representative geometry of the most populated cluster for (3*R*)-**4**.

**Table S1**. Full Cluster Populations (%) for (*S*)- and (*R*)-**4**.

| | c0 | c1 | c2 | c3 | c4 | c5 | c6 | c7 | c8 | c9 |
|---|---|---|---|---|---|---|---|---|---|---|
| (3*S*)-**4** | 67.3 | 18.0 | 5.0 | 4.0 | 2.3 | 1.3 | 1.1 | 0.7 | 0.2 | 0.1 |
| (3*R*)-**4** | 29.2 | 18.0 | 14.7 | 12.1 | 7.9 | 7.4 | 3.9 | 3.4 | 2.7 | 0.7 |

## Simulation of (3*S*)-4 in a biological complex

We investigated on the possibility for peptide (3*S*)-**4** to mimic a PPII helix within a biological complex. As a reference, we chosen the structure of the complex between human platelet profilin (HPP) and a poly-L-proline decamer (L-$Pro_{10}$),[2] for which a crystal structure is available (1AWI.pdb). The complex is formed by two molecules of HPP bound to L-$Pro_{10}$, where this latter adopts a PPII-helix. We performed MD simulations (100 ns) of both the HPP:L-$Pro_{10}$ and HPP:(3*S*)-**4** complexes, the latter obtained by a protein-protein docking approach. The binding energy of both L-$Pro_{10}$ and (3*S*)-**4** was then computed using the Nwat-MMGBSA method.[3] Results confirmed that peptide (3*S*)-**4** can actually behave as a PPII mimic in a biological complex. Figure S3 shows the geometry of the complex between HPP and peptide (3*S*)-**4**, as obtained by a cluster analysis of the last 50 ns of MD trajectory. Binding energies for both L-$Pro_{10}$ and (3*S*)-**4** are reported in Table TS2.

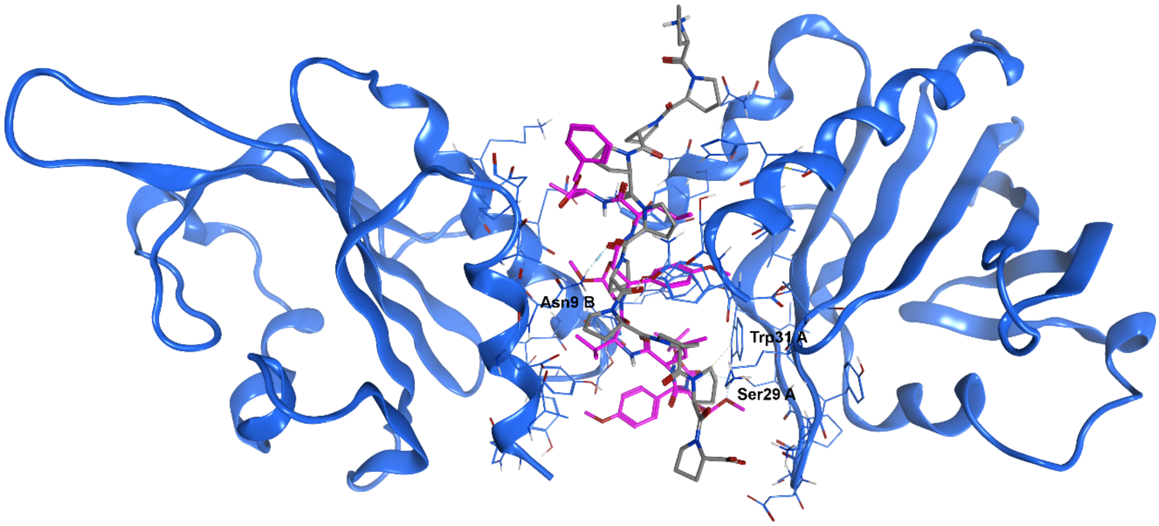


**Figure S3.** Representative geometry of the most populated cluster c0 obtained from the analysis of the last 50 ns of the 100 ns MD trajectory of HPP:(3*S*)-**4** (peptide (3*S*)-**4** carbons are colored in magenta). HPP:L-$Pro_{10}$ was subjected to the same protocol and the representative geometry of the most populated cluster was superposed to HPP:(3*S*)-**4**; but only L-$Pro_{10}$ is shown (grey carbon atoms).

**Table TS2.** Average Energies[a] (kcal/mol) and Standard Deviations Computed by Analyzing the 90-100 ns Segment of the MD trajectory of HPP:(3*S*)-4 and HPP:L-$Pro_{10}$.

| | **HPP:(3*S*)-4** | | **HPP:L-$Pro_{10}$** | |
|---|---|---|---|---|
| | *Nwat=0* | *Nwat=30* | *Nwat=0* | *Nwat=30* |
| $E_{tot}$ *Complex* | -25505.1 ± 48.6 | -25790.9 ± 49.9 | -25879.6 ± 50.8 | -26150.8 ± 50.6 |
| $E_{tot}$ *Receptor* | -25032.4 ± 48.0 | -25291.6 ± 49.2 | -25089.3 ± 51.2 | -25329.1 ± 52.4 |
| $E_{tot}$ *Ligand* | -408.1 ± 6.6 | -408.1 ± 6.6 | -741.4 ± 4.2 | -741.4 ± 4.2 |
| $\Delta E_{binding}$ | **-64.5 ± 4.9** | **-91.2 ± 9.6** | **-48.9 ± 6.5** | **-80.3 ± 10.5** |

[a]Nwat-MMGBSA binding energies were computed considering no explicit waters (Nwat=0) or including 30 explicit waters (Nwat=30). In this case, selected waters are the closest to the ligand in each frame of the MD trajectory and are considered part of the receptor, according to the Nwat-MMGBSA method. In both cases, entropy was neglected and the computed values should be considered as a "score" rather than an absolute binding free energy.

## Computational methods

*Parameterization of 3-Ar-β-Morph.* Charge parameterization for β-Morph was performed using the R.E.D.IV tools.[4] The amino acid structure was capped by acetyl and a NHMe group at the N and C termini, respectively, and subjected to a conformational search using the low mode method, the AMBER10EHT force field and the Born solvation model implemented in MOE.[5] The two conformations corresponding to the *E* and *Z* configurations at the peptide bond linking the acetyl cap to the residue were used for charge parameterization. For each conformation, two orientations were used to derive conformation and orientation independent RESP charges. Gaussian09[6] was used to perform quantum mechanical calculations at the HF/6-31G* level, accordingly to the force field specifications. All the molecular dynamics simulations were conducted with the Amber18 and AmberTools18 packages,[7] using the ff14SB forcefield.[8] Parameters for the peptide bond rotation were modified as suggested by Doshi and Hemelberg.[9]

*Hamiltonian replica exchange molecular* dynamics. H-REMD simulations were conducted starting from the extended configuration of (3*R*)- and (3*S*)-**4**. Twelve different Hamiltonians were generated by progressively lowering the torsional potential of the φ, ψ and ω dihedrals, starting from the default values. All the 12 replicas were subjected to a geometry minimization (1000 cycles of steepest descent and 1000 cycles of conjugated gradient, up to a gradient of 0.1 kcal/mol·Å), followed by constant volume (NVT) equilibration (5 ns, 300 K, Langevin thermostat with a collision frequency = 2.0 $ps^{-1}$, electrostatic cutoff = 8.0 Å, PME, SHAKE to constrain bonds involving hydrogens). A production run of 1.5 μs was then conducted under the same conditions. Simulations were conducted on a cluster of GPU-equipped nodes using the *pmemd.cuda.MPI* executable[10,11] of the Amber18 package.[7] Trajectory analyses were conducted on the final 500 ns of the unmodified replica, using *cpptraj* and *cpptraj.cuda*. Cluster analyses were done requesting 10 clusters, using the average-linkage algorithm and the pairwise mass-weighted root mean squared deviation (RMSD) on the Cα as the metric. Convergence was evaluated by doing a cluster analysis every 500 ns and comparing results in terms of population of the most populated clusters and RMSD between the main cluster representative conformations. All the simulations resulted converged between 1000 and 1500 ns of simulation time.
*Simulation of HPP complexes.* The HPP models containing L-$Pro_{10}$ and (3*S*)-**4** were prepared starting from the 1AWI.pdb file. *HPP:L-$Pro_{10}$ model*: the complex was processed with the Structure Preparation module of the software MOE and protonated at pH=7 using the Protonate3D tool. Waters were removed and the system was minimized up to a gradient of 0.1 kcal/mol·Å, using the Amber10:EHT force field and the Born solvation model for water, and keeping the backbone atoms restrained. The model was used for MD simulations as described below. *HPP*:(3*S*)-**4** *model*: starting from the HPP:L-$Pro_{10}$ model, the L-$Pro_{10}$ chain was removed and the system was subjected to a backbone-restrained minimization, as described above. The system was used as the receptor in a docking experiment using the Protein-Protein docking algorithm implemented in MOE. The representative geometry of the most populated cluster for (*3S*)-**4**, obtained by H-REMD simulations, was used as the ligand to be docked. The top-scored pose showed a decent superposition between (*3S*)-**4** and the L-$Pro_{10}$ chain as found in the crystal structure. Thus, the HPP:(3*S*)-**4** complex was used for the next steps.

The HPP:L-$Pro_{10}$ and HPP:(3*S*)-**4** models described above were used as the starting point for MD simulations. The systems were neutralized by adding 4 Cl- atoms and solvated by an octahedral box of TIP3P water, extending up to 10 Å from the solute. The system was equilibrated as described in previous works,[12] and subjected to 100 ns of MD simulations using *pmemd.cuda* of Amber18. The resulting trajectory was analyzed by computing time dependent RMSDs to verify that systems were stable during the dynamics. Then, the last 50 ns were subjected to a cluster analysis, using the same

protocol described above. Finally, binding energies for L-Pro$_{10}$ and (3*S*)-**4** were computed on the last 10 ns of MD trajectory using the Nwat-MMGBSA approach.[3] Computing the energy on the 50-60 ns segment of the MD trajectory did not lead to relevant changes in the relative binding energies.

## Coordinates of representative geometries

**Representative geometry of cluster c0 for (3*S*)-4**.

```
ATOM      1  O1  BOC    1      11.216  18.685  15.155
ATOM      2  C   BOC    1      11.343  17.472  15.689
ATOM      3  O   BOC    1      10.993  16.471  15.114
ATOM      4  CT  BOC    1      10.889  18.895  13.786
ATOM      5  CT1 BOC    1      11.058  20.365  13.661
ATOM      6  H11 BOC    1      10.545  20.917  14.449
ATOM      7  H12 BOC    1      10.607  20.654  12.711
ATOM      8  H13 BOC    1      12.113  20.637  13.701
ATOM      9  CT2 BOC    1       9.380  18.628  13.658
ATOM     10  H21 BOC    1       8.936  17.658  13.880
ATOM     11  H22 BOC    1       9.022  18.753  12.636
ATOM     12  H23 BOC    1       8.772  19.363  14.185
ATOM     13  CT3 BOC    1      11.652  18.050  12.809
ATOM     14  H31 BOC    1      11.632  18.374  11.768
ATOM     15  H32 BOC    1      11.232  17.047  12.730
ATOM     16  H33 BOC    1      12.662  17.898  13.189
ATOM     17  N   PSS    2      11.785  17.562  16.981
ATOM     18  CE  PSS    2      11.920  16.329  17.744
ATOM     19  HE2 PSS    2      11.486  15.488  17.203
ATOM     20  HE3 PSS    2      11.408  16.449  18.699
ATOM     21  CD  PSS    2      13.369  16.120  18.014
ATOM     22  HD  PSS    2      13.443  15.270  18.692
ATOM     23  OD  PSS    2      14.170  15.806  16.827
ATOM     24  CD1 PSS    2      15.537  15.498  17.056
ATOM     25 HD11 PSS    2      15.575  14.819  17.909
ATOM     26 HD12 PSS    2      16.078  16.429  17.221
ATOM     27 HD13 PSS    2      16.040  14.997  16.229
ATOM     28  OC  PSS    2      14.062  17.202  18.626
ATOM     29  CA  PSS    2      12.374  18.732  17.595
ATOM     30  HA  PSS    2      12.436  19.574  16.905
ATOM     31  CP  PSS    2      13.901  18.430  17.946
ATOM     32  HP  PSS    2      14.308  18.322  16.941
ATOM     33  CB4 PSS    2       9.609  19.856  20.682
ATOM     34  OM  PSS    2       8.671  20.157  21.568
ATOM     35  CM  PSS    2       8.669  19.870  22.908
ATOM     36  HM1 PSS    2       7.779  20.315  23.354
ATOM     37  HM2 PSS    2       9.589  20.284  23.321
ATOM     38  HM3 PSS    2       8.621  18.799  23.105
ATOM     39  CB3 PSS    2      10.729  18.999  21.026
ATOM     40  HB3 PSS    2      10.763  18.655  22.049
ATOM     41  CB2 PSS    2      11.660  18.772  20.051
ATOM     42  HB2 PSS    2      12.394  18.002  20.239
ATOM     43  CB1 PSS    2      11.531  19.202  18.724
ATOM     44  CB6 PSS    2      10.447  20.007  18.374
ATOM     45  HB6 PSS    2      10.368  20.476  17.405
ATOM     46  CB5 PSS    2       9.491  20.377  19.333
ATOM     47  HB5 PSS    2       8.641  21.004  19.110
ATOM     48  C   PSS    2      14.555  19.608  18.632
ATOM     49  O   PSS    2      14.780  20.653  18.070
ATOM     50  N   LEU    3      14.730  19.466  19.955
ATOM     51  H   LEU    3      14.608  18.484  20.154
ATOM     52  CA  LEU    3      15.393  20.329  20.902
ATOM     53  HA  LEU    3      14.978  21.334  20.829
ATOM     54  CB  LEU    3      15.298  19.785  22.332
ATOM     55  HB2 LEU    3      15.822  18.830  22.371
ATOM     56  HB3 LEU    3      15.834  20.544  22.901
ATOM     57  CG  LEU    3      13.914  19.459  22.898
ATOM     58  HG  LEU    3      13.476  18.770  22.176
ATOM     59  CD1 LEU    3      13.932  18.765  24.247
ATOM     60 HD11 LEU    3      14.416  17.799  24.105
ATOM     61 HD12 LEU    3      14.518  19.436  24.875
ATOM     62 HD13 LEU    3      12.971  18.554  24.718
ATOM     63  CD2 LEU    3      13.104  20.729  23.068
ATOM     64 HD21 LEU    3      13.474  21.285  23.930
ATOM     65 HD22 LEU    3      13.009  21.425  22.234
ATOM     66 HD23 LEU    3      12.066  20.418  23.182
ATOM     67  C   LEU    3      16.828  20.615  20.450
ATOM     68  O   LEU    3      17.526  19.642  20.131
ATOM     69  N   VAL    4      17.347  21.851  20.503
ATOM     70  H   VAL    4      16.764  22.599  20.849
ATOM     71  CA  VAL    4      18.724  22.182  19.940
ATOM     72  HA  VAL    4      18.716  21.607  19.014
ATOM     73  CB  VAL    4      18.984  23.618  19.509
ATOM     74  HB  VAL    4      19.974  23.690  19.057
ATOM     75  CG1 VAL    4      17.947  24.009  18.412
ATOM     76 HG11 VAL    4      16.905  24.208  18.663
ATOM     77 HG12 VAL    4      18.396  24.821  17.840
ATOM     78 HG13 VAL    4      17.953  23.140  17.754
ATOM     79  CG2 VAL    4      18.817  24.751  20.561
ATOM     80 HG21 VAL    4      19.279  25.648  20.147
ATOM     81 HG22 VAL    4      17.791  25.033  20.799
ATOM     82 HG23 VAL    4      19.350  24.510  21.480
ATOM     83  C   VAL    4      19.892  21.688  20.842
ATOM     84  O   VAL    4      19.709  21.595  22.048
ATOM     85  N   PSS    5      21.114  21.374  20.342
ATOM     86  CE  PSS    5      21.490  21.346  18.943
ATOM     87  HE2 PSS    5      20.782  21.788  18.243
ATOM     88  HE3 PSS    5      21.574  20.290  18.689
ATOM     89  CD  PSS    5      22.826  21.979  18.590
ATOM     90  HD  PSS    5      23.151  21.769  17.570
ATOM     91  OD  PSS    5      22.547  23.443  18.592
ATOM     92  CD1 PSS    5      23.697  24.183  18.130
ATOM     93 HD11 PSS    5      24.275  23.596  17.416
ATOM     94 HD12 PSS    5      24.281  24.330  19.038
ATOM     95 HD13 PSS    5      23.433  25.171  17.754
ATOM     96  OC  PSS    5      23.843  21.805  19.541
ATOM     97  CA  PSS    5      22.281  21.030  21.229
ATOM     98  HA  PSS    5      22.075  21.383  22.239
ATOM     99  CP  PSS    5      23.379  21.970  20.903
ATOM    100  HP  PSS    5      23.023  22.992  21.033
ATOM    101  CB4 PSS    5      23.262  16.770  21.905
ATOM    102  OM  PSS    5      23.588  15.505  22.026
ATOM    103  CM  PSS    5      22.976  14.698  22.993
ATOM    104  HM1 PSS    5      21.903  14.844  23.114
ATOM    105  HM2 PSS    5      23.275  15.069  23.973
ATOM    106  HM3 PSS    5      23.395  13.693  22.939
ATOM    107  CB3 PSS    5      22.310  17.489  22.765
ATOM    108  HB3 PSS    5      21.778  16.965  23.545
ATOM    109  CB2 PSS    5      21.888  18.781  22.435
```

```
ATOM    110  HB2 PSS     5      21.122  19.292  22.999
ATOM    111  CB1 PSS     5      22.476  19.498  21.355
ATOM    112  CB6 PSS     5      23.436  18.858  20.535
ATOM    113  HB6 PSS     5      23.848  19.480  19.754
ATOM    114  CB5 PSS     5      23.758  17.495  20.761
ATOM    115  HB5 PSS     5      24.376  16.954  20.060
ATOM    116  C   PSS     5      24.495  21.881  21.930
ATOM    117  O   PSS     5      24.496  22.706  22.836
ATOM    118  N   LEU     6      25.412  20.934  21.875
ATOM    119  H   LEU     6      25.406  20.488  20.969
ATOM    120  CA  LEU     6      26.478  20.603  22.807
ATOM    121  HA  LEU     6      26.242  20.818  23.849
ATOM    122  CB  LEU     6      26.953  19.135  22.782
ATOM    123  HB2 LEU     6      27.140  18.846  21.748
ATOM    124  HB3 LEU     6      27.892  19.165  23.335
ATOM    125  CG  LEU     6      26.047  18.145  23.543
ATOM    126  HG  LEU     6      25.003  18.313  23.280
ATOM    127  CD1 LEU     6      26.639  16.733  23.363
ATOM    128 HD11 LEU     6      26.412  16.422  22.344
ATOM    129 HD12 LEU     6      27.729  16.755  23.343
ATOM    130 HD13 LEU     6      26.218  15.993  24.043
ATOM    131  CD2 LEU     6      26.013  18.338  25.102
ATOM    132 HD21 LEU     6      26.981  18.767  25.361
ATOM    133 HD22 LEU     6      25.238  19.005  25.479
ATOM    134 HD23 LEU     6      25.842  17.455  25.718
ATOM    135  C   LEU     6      27.667  21.513  22.381
ATOM    136  O   LEU     6      27.811  21.620  21.171
ATOM    137  N   VAL     7      28.485  21.954  23.311
ATOM    138  H   VAL     7      28.255  21.945  24.295
ATOM    139  CA  VAL     7      29.697  22.693  22.942
ATOM    140  HA  VAL     7      29.402  23.235  22.044
ATOM    141  CB  VAL     7      30.291  23.547  24.072
ATOM    142  HB  VAL     7      30.870  22.955  24.781
ATOM    143  CG1 VAL     7      31.230  24.574  23.409
ATOM    144 HG11 VAL     7      31.644  25.289  24.120
ATOM    145 HG12 VAL     7      32.035  24.015  22.932
ATOM    146 HG13 VAL     7      30.615  25.109  22.686
ATOM    147  CG2 VAL     7      29.262  24.304  24.866
ATOM    148 HG21 VAL     7      29.803  24.892  25.607
ATOM    149 HG22 VAL     7      28.696  25.069  24.334
ATOM    150 HG23 VAL     7      28.533  23.621  25.302
ATOM    151  C   VAL     7      30.793  21.805  22.398
ATOM    152  O   VAL     7      31.124  20.861  23.068
ATOM    153  OS  BZN     8      31.335  22.096  21.277
ATOM    154  CB  BZN     8      32.370  21.283  20.568
ATOM    155  HB2 BZN     8      31.884  20.432  20.089
ATOM    156  HB3 BZN     8      33.040  20.952  21.360
ATOM    157  CG  BZN     8      33.174  22.083  19.514
ATOM    158  CD1 BZN     8      32.567  23.147  18.848
ATOM    159  HD1 BZN     8      31.533  23.421  18.998
ATOM    160  CE1 BZN     8      33.370  23.939  17.990
ATOM    161  HE1 BZN     8      32.924  24.764  17.455
ATOM    162  CZ  BZN     8      34.720  23.735  17.804
ATOM    163  HZ  BZN     8      35.346  24.386  17.211
ATOM    164  CE2 BZN     8      35.312  22.680  18.430
ATOM    165  HE2 BZN     8      36.361  22.495  18.255
ATOM    166  CD2 BZN     8      34.582  21.947  19.409
ATOM    167  HD2 BZN     8      35.186  21.155  19.827
TER     168      BZN     8
END
```

**Representative geometry of cluster c0 for (3*R*)-4.**

```
ATOM      1  O1  BOC     1      25.669  18.205  21.652
ATOM      2  C   BOC     1      26.095  18.633  20.464
ATOM      3  O   BOC     1      27.198  18.402  20.059
ATOM      4  CT  BOC     1      26.572  17.521  22.571
ATOM      5  CT1 BOC     1      27.351  16.290  22.102
ATOM      6  H11 BOC     1      26.636  15.575  21.694
ATOM      7  H12 BOC     1      27.772  15.815  22.988
ATOM      8  H13 BOC     1      28.140  16.499  21.380
ATOM      9  CT2 BOC     1      25.800  17.055  23.735
ATOM     10  H21 BOC     1      25.475  17.929  24.299
ATOM     11  H22 BOC     1      26.353  16.480  24.478
ATOM     12  H23 BOC     1      24.883  16.525  23.476
ATOM     13  CT3 BOC     1      27.595  18.658  23.029
ATOM     14  H31 BOC     1      28.023  18.524  24.023
ATOM     15  H32 BOC     1      27.097  19.628  23.007
ATOM     16  H33 BOC     1      28.343  18.833  22.256
ATOM     17  N   PSR     2      25.154  19.414  19.842
ATOM     18  CE  PSR     2      25.519  20.078  18.612
ATOM     19  HE2 PSR     2      24.830  19.653  17.882
ATOM     20  HE3 PSR     2      26.509  19.861  18.211
ATOM     21  CD  PSR     2      25.421  21.587  18.781
ATOM     22  HD  PSR     2      26.133  21.917  19.537
ATOM     23  OD  PSR     2      25.446  22.225  17.566
ATOM     24  CD1 PSR     2      26.771  22.601  17.256
ATOM     25 HD11 PSR     2      26.678  23.096  16.289
ATOM     26 HD12 PSR     2      27.455  21.758  17.159
ATOM     27 HD13 PSR     2      27.254  23.224  18.010
ATOM     28  OC  PSR     2      24.114  21.980  19.292
ATOM     29  CA  PSR     2      23.789  19.775  20.431
ATOM     30  HA  PSR     2      23.733  19.309  21.415
ATOM     31  CP  PSR     2      23.723  21.319  20.496
ATOM     32  HP  PSR     2      22.698  21.605  20.729
ATOM     33  CB4 PSR     2      20.644  17.828  18.122
ATOM     34  OM  PSR     2      19.705  17.374  17.251
ATOM     35  CM  PSR     2      19.489  15.963  17.120
ATOM     36  HM1 PSR     2      18.992  15.793  16.165
ATOM     37  HM2 PSR     2      18.813  15.576  17.883
ATOM     38  HM3 PSR     2      20.457  15.462  17.111
ATOM     39  CB3 PSR     2      21.525  17.069  18.888
ATOM     40  HB3 PSR     2      21.483  15.990  18.908
ATOM     41  CB2 PSR     2      22.427  17.721  19.705
ATOM     42  HB2 PSR     2      23.129  17.120  20.264
ATOM     43  CB1 PSR     2      22.629  19.133  19.677
ATOM     44  CB6 PSR     2      21.824  19.876  18.766
ATOM     45  HB6 PSR     2      21.911  20.941  18.610
ATOM     46  CB5 PSR     2      20.838  19.196  18.003
ATOM     47  HB5 PSR     2      20.135  19.656  17.324
ATOM     48  C   PSR     2      24.497  22.097  21.509
ATOM     49  O   PSR     2      25.487  21.569  22.066
ATOM     50  N   LEU     3      24.178  23.368  21.764
ATOM     51  H   LEU     3      23.349  23.758  21.339
ATOM     52  CA  LEU     3      24.910  24.254  22.621
ATOM     53  HA  LEU     3      25.853  23.785  22.903
ATOM     54  CB  LEU     3      25.191  25.509  21.818
ATOM     55  HB2 LEU     3      24.294  26.115  21.695
ATOM     56  HB3 LEU     3      25.735  26.188  22.474
ATOM     57  CG  LEU     3      25.937  25.309  20.446
ATOM     58  HG  LEU     3      25.247  24.802  19.772
ATOM     59  CD1 LEU     3      26.244  26.668  19.887
ATOM     60 HD11 LEU     3      25.378  27.025  19.331
```

```
ATOM     61 HD12 LEU    3      26.488  27.313  20.731
ATOM     62 HD13 LEU    3      27.072  26.715  19.179
ATOM     63  CD2 LEU    3      27.263  24.563  20.568
ATOM     64 HD21 LEU    3      27.717  24.607  19.578
ATOM     65 HD22 LEU    3      27.876  25.052  21.325
ATOM     66 HD23 LEU    3      27.060  23.539  20.884
ATOM     67  C   LEU    3      24.199  24.633  23.914
ATOM     68  O   LEU    3      24.805  24.809  24.968
ATOM     69  N   VAL    4      22.867  24.720  23.834
ATOM     70  H   VAL    4      22.495  24.614  22.901
ATOM     71  CA  VAL    4      22.002  25.102  24.977
ATOM     72  HA  VAL    4      22.621  24.974  25.864
ATOM     73  CB  VAL    4      21.425  26.550  24.993
ATOM     74  HB  VAL    4      20.748  26.702  24.152
ATOM     75  CG1 VAL    4      20.709  26.833  26.285
ATOM     76 HG11 VAL    4      21.417  26.826  27.113
ATOM     77 HG12 VAL    4      20.351  27.859  26.195
ATOM     78 HG13 VAL    4      19.915  26.158  26.603
ATOM     79  CG2 VAL    4      22.702  27.404  24.963
ATOM     80 HG21 VAL    4      23.216  27.542  24.011
ATOM     81 HG22 VAL    4      22.370  28.419  25.184
ATOM     82 HG23 VAL    4      23.359  26.999  25.732
ATOM     83  C   VAL    4      20.911  24.002  25.071
ATOM     84  O   VAL    4      19.825  24.247  24.517
ATOM     85  N   PSR    5      21.116  22.849  25.686
ATOM     86  CE  PSR    5      22.446  22.379  26.202
ATOM     87  HE2 PSR    5      22.254  22.155  27.251
ATOM     88  HE3 PSR    5      23.257  23.089  26.044
ATOM     89  CD  PSR    5      22.849  21.125  25.453
ATOM     90  HD  PSR    5      23.103  21.381  24.425
ATOM     91  OD  PSR    5      24.011  20.517  26.102
ATOM     92  CD1 PSR    5      25.238  20.661  25.503
ATOM     93 HD11 PSR    5      25.150  20.728  24.418
ATOM     94 HD12 PSR    5      25.891  19.849  25.822
ATOM     95 HD13 PSR    5      25.509  21.667  25.822
ATOM     96  OC  PSR    5      21.825  20.057  25.585
ATOM     97  CA  PSR    5      20.067  21.783  25.884
ATOM     98  HA  PSR    5      19.158  22.077  25.357
ATOM     99  CP  PSR    5      20.558  20.515  25.155
ATOM    100  HP  PSR    5      19.728  19.811  25.208
ATOM    101  CB4 PSR    5      18.918  21.421  30.125
ATOM    102  OM  PSR    5      18.511  21.337  31.467
ATOM    103  CM  PSR    5      17.685  22.374  32.013
ATOM    104  HM1 PSR    5      18.196  23.327  32.149
ATOM    105  HM2 PSR    5      17.336  22.039  32.990
ATOM    106  HM3 PSR    5      16.796  22.438  31.385
ATOM    107  CB3 PSR    5      18.332  22.375  29.254
ATOM    108  HB3 PSR    5      17.537  23.052  29.529
ATOM    109  CB2 PSR    5      18.703  22.412  27.900
ATOM    110  HB2 PSR    5      18.279  23.204  27.301
ATOM    111  CB1 PSR    5      19.762  21.572  27.387
ATOM    112  CB6 PSR    5      20.305  20.591  28.200
ATOM    113  HB6 PSR    5      20.895  19.802  27.759
ATOM    114  CB5 PSR    5      19.875  20.524  29.580
ATOM    115  HB5 PSR    5      20.251  19.761  30.245
ATOM    116  C   PSR    5      20.581  20.680  23.578
ATOM    117  O   PSR    5      21.592  20.969  22.894
ATOM    118  N   LEU    6      19.390  20.429  22.992
ATOM    119  H   LEU    6      18.586  20.266  23.581
ATOM    120  CA  LEU    6      19.214  20.376  21.551
ATOM    121  HA  LEU    6      19.991  19.764  21.092
ATOM    122  CB  LEU    6      17.814  19.808  21.399
ATOM    123  HB2 LEU    6      17.078  20.479  21.843
ATOM    124  HB3 LEU    6      17.530  19.779  20.347
ATOM    125  CG  LEU    6      17.627  18.352  21.861
ATOM    126  HG  LEU    6      18.253  18.193  22.740
ATOM    127  CD1 LEU    6      16.123  18.044  22.201
ATOM    128 HD11 LEU    6      15.987  17.000  22.482
ATOM    129 HD12 LEU    6      15.882  18.739  23.005
ATOM    130 HD13 LEU    6      15.431  18.236  21.381
ATOM    131  CD2 LEU    6      18.258  17.455  20.775
ATOM    132 HD21 LEU    6      19.320  17.648  20.628
ATOM    133 HD22 LEU    6      18.147  16.431  21.133
ATOM    134 HD23 LEU    6      17.672  17.453  19.856
ATOM    135  C   LEU    6      19.275  21.708  20.755
ATOM    136  O   LEU    6      19.257  21.591  19.522
ATOM    137  N   VAL    7      19.347  22.895  21.329
ATOM    138  H   VAL    7      19.174  23.018  22.317
ATOM    139  CA  VAL    7      19.485  24.097  20.457
ATOM    140  HA  VAL    7      18.945  24.110  19.510
ATOM    141  CB  VAL    7      18.937  25.374  21.189
ATOM    142  HB  VAL    7      19.465  25.556  22.125
ATOM    143  CG1 VAL    7      19.084  26.637  20.340
ATOM    144 HG11 VAL    7      18.549  26.498  19.401
ATOM    145 HG12 VAL    7      18.616  27.530  20.755
ATOM    146 HG13 VAL    7      20.154  26.775  20.187
ATOM    147  CG2 VAL    7      17.368  25.269  21.459
ATOM    148 HG21 VAL    7      17.089  26.028  22.190
ATOM    149 HG22 VAL    7      16.848  25.371  20.507
ATOM    150 HG23 VAL    7      17.034  24.320  21.878
ATOM    151  C   VAL    7      20.950  24.309  19.999
ATOM    152  O   VAL    7      21.850  24.499  20.840
ATOM    153  OS  BZN    8      21.098  24.340  18.667
ATOM    154  CB  BZN    8      22.354  24.605  18.125
ATOM    155  HB2 BZN    8      22.557  24.068  17.198
ATOM    156  HB3 BZN    8      23.183  24.331  18.778
ATOM    157  CG  BZN    8      22.383  26.040  17.786
ATOM    158  CD1 BZN    8      22.847  27.008  18.769
ATOM    159  HD1 BZN    8      23.079  26.680  19.772
ATOM    160  CE1 BZN    8      22.864  28.351  18.319
ATOM    161  HE1 BZN    8      23.162  29.145  18.987
ATOM    162  CZ  BZN    8      22.458  28.702  17.037
ATOM    163  HZ  BZN    8      22.445  29.764  16.840
ATOM    164  CE2 BZN    8      21.987  27.795  16.124
ATOM    165  HE2 BZN    8      21.636  28.097  15.148
ATOM    166  CD2 BZN    8      21.936  26.420  16.529
ATOM    167  HD2 BZN    8      21.526  25.696  15.840
TER     168      BZN    8
END
```

**Representative geometry of cluster c0 for HPP:(3*S*)-4 (binding site residues up to 4.5 Å to the ligand are reported; hydrogen atoms are omitted)**

```
ATOM      1  N   TRP    2      62.752  33.807  64.252
ATOM      2  CA  TRP    2      62.133  33.346  65.528
ATOM      3  CB  TRP    2      62.065  31.869  65.686
ATOM      4  CG  TRP    2      61.245  31.132  64.666
ATOM      5  CD1 TRP    2      61.659  30.552  63.508
ATOM      6  NE1 TRP    2      60.556  30.050  62.884
ATOM      7  CE2 TRP    2      59.436  30.301  63.553
ATOM      8  CZ2 TRP    2      58.058  30.107  63.331
ATOM      9  CH2 TRP    2      57.099  30.524  64.257
```

| | | | | | | | |
|---|---|---|---|---|---|---|---|
| ATOM | 10 | CZ3 | TRP | 2 | 57.457 | 31.095 | 65.450 |
| ATOM | 11 | CE3 | TRP | 2 | 58.873 | 31.246 | 65.712 |
| ATOM | 12 | CD2 | TRP | 2 | 59.833 | 30.963 | 64.760 |
| ATOM | 13 | C | TRP | 2 | 62.643 | 34.068 | 66.738 |
| ATOM | 14 | O | TRP | 2 | 61.795 | 34.490 | 67.502 |
| ATOM | 15 | N | TYR | 5 | 61.216 | 38.054 | 67.094 |
| ATOM | 16 | CA | TYR | 5 | 59.844 | 38.319 | 67.461 |
| ATOM | 17 | CB | TYR | 5 | 58.961 | 37.130 | 67.024 |
| ATOM | 18 | CG | TYR | 5 | 59.065 | 36.751 | 65.538 |
| ATOM | 19 | CD1 | TYR | 5 | 59.332 | 37.712 | 64.486 |
| ATOM | 20 | CE1 | TYR | 5 | 59.453 | 37.293 | 63.149 |
| ATOM | 21 | CZ | TYR | 5 | 59.405 | 35.890 | 62.829 |
| ATOM | 22 | OH | TYR | 5 | 59.739 | 35.471 | 61.623 |
| ATOM | 23 | CE2 | TYR | 5 | 59.157 | 34.952 | 63.853 |
| ATOM | 24 | CD2 | TYR | 5 | 59.036 | 35.361 | 65.155 |
| ATOM | 25 | C | TYR | 5 | 59.752 | 38.599 | 68.966 |
| ATOM | 26 | O | TYR | 5 | 59.309 | 39.642 | 69.397 |
| ATOM | 27 | N | ILE | 20 | 56.468 | 32.656 | 73.019 |
| ATOM | 28 | CA | ILE | 20 | 55.517 | 32.340 | 71.925 |
| ATOM | 29 | CB | ILE | 20 | 55.930 | 32.898 | 70.544 |
| ATOM | 30 | CG2 | ILE | 20 | 54.913 | 32.375 | 69.473 |
| ATOM | 31 | CG1 | ILE | 20 | 56.001 | 34.454 | 70.603 |
| ATOM | 32 | CD1 | ILE | 20 | 56.452 | 35.100 | 69.314 |
| ATOM | 33 | C | ILE | 20 | 55.410 | 30.811 | 71.874 |
| ATOM | 34 | O | ILE | 20 | 56.328 | 30.059 | 71.579 |
| ATOM | 35 | N | SER | 26 | 50.050 | 18.975 | 68.694 |
| ATOM | 36 | CA | SER | 26 | 51.058 | 18.610 | 69.659 |
| ATOM | 37 | CB | SER | 26 | 52.116 | 17.569 | 69.060 |
| ATOM | 38 | OG | SER | 26 | 52.965 | 17.027 | 70.088 |
| ATOM | 39 | C | SER | 26 | 51.798 | 19.932 | 69.942 |
| ATOM | 40 | O | SER | 26 | 52.328 | 20.550 | 68.990 |
| ATOM | 41 | N | PRO | 27 | 51.732 | 20.439 | 71.237 |
| ATOM | 42 | CD | PRO | 27 | 50.983 | 19.941 | 72.308 |
| ATOM | 43 | CG | PRO | 27 | 50.946 | 20.949 | 73.475 |
| ATOM | 44 | CB | PRO | 27 | 52.259 | 21.692 | 73.122 |
| ATOM | 45 | CA | PRO | 27 | 52.475 | 21.630 | 71.554 |
| ATOM | 46 | C | PRO | 27 | 53.956 | 21.777 | 71.150 |
| ATOM | 47 | O | PRO | 27 | 54.725 | 20.831 | 71.151 |
| ATOM | 48 | N | SER | 28 | 54.413 | 23.022 | 71.128 |
| ATOM | 49 | CA | SER | 28 | 55.861 | 23.365 | 71.025 |
| ATOM | 50 | CB | SER | 28 | 56.337 | 23.063 | 69.579 |
| ATOM | 51 | OG | SER | 28 | 57.732 | 23.308 | 69.536 |
| ATOM | 52 | C | SER | 28 | 56.160 | 24.885 | 71.287 |
| ATOM | 53 | O | SER | 28 | 55.449 | 25.784 | 70.765 |
| ATOM | 54 | N | TRP | 30 | 57.728 | 27.722 | 70.490 |
| ATOM | 55 | CA | TRP | 30 | 58.305 | 28.356 | 69.217 |
| ATOM | 56 | CB | TRP | 30 | 57.176 | 28.957 | 68.420 |
| ATOM | 57 | CG | TRP | 30 | 56.314 | 27.951 | 67.735 |
| ATOM | 58 | CD1 | TRP | 30 | 56.517 | 26.621 | 67.672 |
| ATOM | 59 | NE1 | TRP | 30 | 55.435 | 26.011 | 67.080 |
| ATOM | 60 | CE2 | TRP | 30 | 54.399 | 26.873 | 66.884 |
| ATOM | 61 | CZ2 | TRP | 30 | 53.094 | 26.751 | 66.307 |
| ATOM | 62 | CH2 | TRP | 30 | 52.337 | 27.905 | 66.020 |
| ATOM | 63 | CZ3 | TRP | 30 | 52.944 | 29.112 | 66.318 |
| ATOM | 64 | CE3 | TRP | 30 | 54.264 | 29.274 | 66.774 |
| ATOM | 65 | CD2 | TRP | 30 | 54.989 | 28.141 | 67.179 |
| ATOM | 66 | C | TRP | 30 | 59.359 | 29.442 | 69.490 |
| ATOM | 67 | O | TRP | 30 | 60.287 | 29.612 | 68.700 |
| ATOM | 68 | N | LYS | 106 | 45.977 | 29.959 | 66.367 |
| ATOM | 69 | CA | LYS | 106 | 47.161 | 29.189 | 66.176 |
| ATOM | 70 | CB | LYS | 106 | 47.825 | 29.381 | 64.729 |
| ATOM | 71 | CG | LYS | 106 | 46.829 | 28.981 | 63.594 |
| ATOM | 72 | CD | LYS | 106 | 47.346 | 28.888 | 62.176 |
| ATOM | 73 | CE | LYS | 106 | 46.397 | 28.230 | 61.198 |
| ATOM | 74 | NZ | LYS | 106 | 45.260 | 29.162 | 61.059 |
| ATOM | 75 | C | LYS | 106 | 48.272 | 29.443 | 67.219 |
| ATOM | 76 | O | LYS | 106 | 49.175 | 28.640 | 67.333 |
| ATOM | 77 | N | THR | 107 | 48.135 | 30.542 | 68.036 |
| ATOM | 78 | CA | THR | 107 | 49.288 | 30.933 | 68.857 |
| ATOM | 79 | CB | THR | 107 | 49.933 | 32.240 | 68.109 |
| ATOM | 80 | CG2 | THR | 107 | 51.447 | 32.393 | 68.469 |
| ATOM | 81 | OG1 | THR | 107 | 49.842 | 32.178 | 66.704 |
| ATOM | 82 | C | THR | 107 | 48.801 | 31.072 | 70.284 |
| ATOM | 83 | O | THR | 107 | 47.644 | 31.357 | 70.559 |
| ATOM | 84 | N | HIE | 132 | 48.004 | 41.645 | 66.464 |
| ATOM | 85 | CA | HIE | 132 | 49.044 | 41.523 | 65.388 |
| ATOM | 86 | CB | HIE | 132 | 50.443 | 42.026 | 65.776 |
| ATOM | 87 | CG | HIE | 132 | 51.626 | 41.774 | 64.837 |
| ATOM | 88 | ND1 | HIE | 132 | 51.714 | 42.185 | 63.578 |
| ATOM | 89 | CE1 | HIE | 132 | 52.842 | 41.645 | 63.025 |
| ATOM | 90 | NE2 | HIE | 132 | 53.459 | 40.917 | 63.975 |
| ATOM | 91 | CD2 | HIE | 132 | 52.664 | 40.952 | 65.103 |
| ATOM | 92 | C | HIE | 132 | 49.241 | 40.084 | 64.921 |
| ATOM | 93 | O | HIE | 132 | 49.441 | 39.781 | 63.832 |
| ATOM | 94 | N | LEU | 133 | 49.130 | 39.107 | 65.836 |
| ATOM | 95 | CA | LEU | 133 | 48.912 | 37.723 | 65.453 |
| ATOM | 96 | CB | LEU | 133 | 48.969 | 36.841 | 66.669 |
| ATOM | 97 | CG | LEU | 133 | 50.443 | 36.628 | 67.120 |
| ATOM | 98 | CD1 | LEU | 133 | 50.449 | 36.083 | 68.578 |
| ATOM | 99 | CD2 | LEU | 133 | 51.251 | 35.649 | 66.178 |
| ATOM | 100 | C | LEU | 133 | 47.596 | 37.527 | 64.770 |
| ATOM | 101 | O | LEU | 133 | 47.607 | 37.116 | 63.622 |
| ATOM | 102 | N | SER | 136 | 47.376 | 38.764 | 61.173 |
| ATOM | 103 | CA | SER | 136 | 48.270 | 37.855 | 60.441 |
| ATOM | 104 | CB | SER | 136 | 49.639 | 37.662 | 61.154 |
| ATOM | 105 | OG | SER | 136 | 50.133 | 39.000 | 61.402 |
| ATOM | 106 | C | SER | 136 | 47.678 | 36.456 | 60.251 |
| ATOM | 107 | O | SER | 136 | 48.372 | 35.636 | 59.570 |
| ATOM | 108 | N | TYR | 138 | 46.986 | 33.977 | 62.355 |
| ATOM | 109 | CA | TYR | 138 | 47.635 | 33.081 | 63.325 |
| ATOM | 110 | CB | TYR | 138 | 49.182 | 33.317 | 63.387 |
| ATOM | 111 | CG | TYR | 138 | 49.964 | 33.452 | 62.085 |
| ATOM | 112 | CD1 | TYR | 138 | 50.883 | 34.517 | 61.997 |
| ATOM | 113 | CE1 | TYR | 138 | 51.646 | 34.701 | 60.837 |
| ATOM | 114 | CZ | TYR | 138 | 51.478 | 33.866 | 59.722 |
| ATOM | 115 | OH | TYR | 138 | 52.290 | 33.882 | 58.609 |
| ATOM | 116 | CE2 | TYR | 138 | 50.628 | 32.718 | 59.846 |
| ATOM | 117 | CD2 | TYR | 138 | 49.922 | 32.520 | 61.059 |
| ATOM | 118 | C | TYR | 138 | 46.910 | 33.193 | 64.644 |
| ATOM | 119 | O | TYR | 138 | 47.189 | 32.393 | 65.606 |
| ATOM | 120 | OXT | TYR | 138 | 46.062 | 34.088 | 64.818 |
| TER | 121 | | TYR | 138 | | | |
| ATOM | 122 | N | TRP | 140 | 55.464 | 21.729 | 51.286 |
| ATOM | 123 | CA | TRP | 140 | 56.194 | 22.768 | 50.665 |
| ATOM | 124 | CB | TRP | 140 | 57.462 | 22.335 | 49.936 |
| ATOM | 125 | CG | TRP | 140 | 58.413 | 21.482 | 50.742 |
| ATOM | 126 | CD1 | TRP | 140 | 58.748 | 20.172 | 50.454 |
| ATOM | 127 | NE1 | TRP | 140 | 59.722 | 19.756 | 51.361 |
| ATOM | 128 | CE2 | TRP | 140 | 60.112 | 20.734 | 52.194 |
| ATOM | 129 | CZ2 | TRP | 140 | 60.979 | 20.760 | 53.266 |
| ATOM | 130 | CH2 | TRP | 140 | 61.120 | 21.964 | 53.940 |
| ATOM | 131 | CZ3 | TRP | 140 | 60.255 | 23.080 | 53.612 |

```
ATOM    132  CE3 TRP   140      59.351  23.030  52.578
ATOM    133  CD2 TRP   140      59.274  21.884  51.845
ATOM    134  C   TRP   140      55.319  23.729  49.751
ATOM    135  O   TRP   140      55.753  24.813  49.430
ATOM    136  N   ALA   142      52.621  25.228  50.780
ATOM    137  CA  ALA   142      52.323  26.184  51.813
ATOM    138  CB  ALA   142      52.091  25.537  53.208
ATOM    139  C   ALA   142      53.368  27.247  52.059
ATOM    140  O   ALA   142      53.039  28.428  52.293
ATOM    141  N   TYR   143      54.618  26.853  51.829
ATOM    142  CA  TYR   143      55.736  27.777  51.847
ATOM    143  CB  TYR   143      57.065  26.967  52.085
ATOM    144  CG  TYR   143      57.323  26.325  53.464
ATOM    145  CD1 TYR   143      58.512  26.517  54.124
ATOM    146  CE1 TYR   143      58.571  26.197  55.494
ATOM    147  CZ  TYR   143      57.595  25.446  56.069
ATOM    148  OH  TYR   143      57.699  25.132  57.351
ATOM    149  CE2 TYR   143      56.455  25.091  55.320
ATOM    150  CD2 TYR   143      56.284  25.507  54.016
ATOM    151  C   TYR   143      55.957  28.603  50.613
ATOM    152  O   TYR   143      56.536  29.687  50.717
ATOM    153  N   ASN   146      53.282  31.560  51.313
ATOM    154  CA  ASN   146      53.946  32.561  52.173
ATOM    155  CB  ASN   146      54.734  31.931  53.324
ATOM    156  CG  ASN   146      55.194  32.895  54.389
ATOM    157  OD1 ASN   146      56.362  33.187  54.473
ATOM    158  ND2 ASN   146      54.325  33.394  55.267
ATOM    159  C   ASN   146      54.809  33.524  51.263
ATOM    160  O   ASN   146      54.645  34.727  51.443
ATOM    161  N   ASP   150      52.675  38.851  50.465
ATOM    162  CA  ASP   150      52.818  40.288  50.733
ATOM    163  CB  ASP   150      54.012  40.558  51.722
ATOM    164  CG  ASP   150      55.353  40.275  51.141
ATOM    165  OD1 ASP   150      56.325  40.739  51.745
ATOM    166  OD2 ASP   150      55.561  39.591  50.112
ATOM    167  C   ASP   150      53.010  41.121  49.443
ATOM    168  O   ASP   150      53.180  42.311  49.475
ATOM    169  N   LYS   263      64.085  38.430  52.224
ATOM    170  CA  LYS   263      62.974  37.415  52.193
ATOM    171  CB  LYS   263      61.892  37.970  51.280
ATOM    172  CG  LYS   263      60.465  37.301  51.626
ATOM    173  CD  LYS   263      59.416  37.601  50.524
ATOM    174  CE  LYS   263      57.980  37.173  50.973
ATOM    175  NZ  LYS   263      57.402  38.089  51.924
ATOM    176  C   LYS   263      63.367  36.041  51.809
ATOM    177  O   LYS   263      62.844  35.067  52.323
ATOM    178  N   GLU   266      65.497  34.955  55.444
ATOM    179  CA  GLU   266      64.529  34.788  56.511
ATOM    180  CB  GLU   266      63.647  36.007  56.477
ATOM    181  CG  GLU   266      64.393  37.178  57.232
ATOM    182  CD  GLU   266      64.600  36.910  58.694
ATOM    183  OE1 GLU   266      65.328  37.771  59.195
ATOM    184  OE2 GLU   266      64.111  35.989  59.321
ATOM    185  C   GLU   266      63.772  33.405  56.302
ATOM    186  O   GLU   266      63.606  32.721  57.292
ATOM    187  N   MET   267      63.555  32.937  55.058
ATOM    188  CA  MET   267      63.005  31.650  54.726
ATOM    189  CB  MET   267      62.410  31.613  53.326
ATOM    190  CG  MET   267      61.879  30.243  52.908
ATOM    191  SD  MET   267      60.464  29.633  53.854
ATOM    192  CE  MET   267      59.183  30.664  53.280
ATOM    193  C   MET   267      64.100  30.639  54.901
ATOM    194  O   MET   267      63.790  29.671  55.554
ATOM    195  N   HIE   270      64.275  30.146  58.775
ATOM    196  CA  HIE   270      63.225  29.279  59.374
ATOM    197  CB  HIE   270      61.818  29.690  58.821
ATOM    198  CG  HIE   270      60.735  28.821  59.238
ATOM    199  ND1 HIE   270      60.553  28.324  60.530
ATOM    200  CE1 HIE   270      59.481  27.504  60.432
ATOM    201  NE2 HIE   270      59.010  27.521  59.157
ATOM    202  CD2 HIE   270      59.746  28.379  58.414
ATOM    203  C   HIE   270      63.499  27.925  58.968
ATOM    204  O   HIE   270      63.433  27.072  59.855
ATOM    205  N   ARG   273      66.329  26.708  61.237
ATOM    206  CA  ARG   273      65.793  26.465  62.648
ATOM    207  CB  ARG   273      65.085  27.773  63.209
ATOM    208  CG  ARG   273      64.671  27.606  64.668
ATOM    209  CD  ARG   273      65.871  27.745  65.563
ATOM    210  NE  ARG   273      65.505  28.039  66.978
ATOM    211  CZ  ARG   273      66.321  28.479  67.923
ATOM    212  NH1 ARG   273      67.621  28.313  67.765
ATOM    213  NH2 ARG   273      65.940  29.062  69.007
ATOM    214  C   ARG   273      64.955  25.197  62.668
ATOM    215  O   ARG   273      65.150  24.351  63.544
ATOM    216  N   SER   274      64.178  24.915  61.623
ATOM    217  CA  SER   274      63.656  23.485  61.364
ATOM    218  CB  SER   274      62.462  23.580  60.398
ATOM    219  OG  SER   274      61.633  24.605  60.841
ATOM    220  C   SER   274      64.641  22.298  61.023
ATOM    221  O   SER   274      64.047  21.363  60.494
ATOM    222  N   TYR   276      67.005  22.077  58.580
ATOM    223  CA  TYR   276      67.357  21.963  57.161
ATOM    224  CB  TYR   276      66.229  22.594  56.236
ATOM    225  CG  TYR   276      65.048  21.655  56.150
ATOM    226  CD1 TYR   276      64.015  21.629  57.119
ATOM    227  CE1 TYR   276      63.062  20.664  57.098
ATOM    228  CZ  TYR   276      63.011  19.718  56.020
ATOM    229  OH  TYR   276      62.097  18.732  56.130
ATOM    230  CE2 TYR   276      63.888  19.846  54.971
ATOM    231  CD2 TYR   276      64.886  20.795  55.011
ATOM    232  C   TYR   276      68.776  22.574  56.938
ATOM    233  O   TYR   276      69.154  22.668  55.758
ATOM    234  OXT TYR   276      69.430  22.993  57.925
TER     235      TYR   276
ATOM    236  O1  BOC   277      58.903  25.119  64.686
ATOM    237  C   BOC   277      58.390  24.100  65.303
ATOM    238  O   BOC   277      58.794  23.768  66.457
ATOM    239  CT  BOC   277      60.140  25.836  64.981
ATOM    240  CT1 BOC   277      59.925  26.793  66.089
ATOM    241  CT2 BOC   277      60.579  26.664  63.754
ATOM    242  CT3 BOC   277      61.351  24.897  65.261
ATOM    243  N   PSS   278      57.447  23.450  64.591
ATOM    244  CE  PSS   278      56.712  22.235  65.140
ATOM    245  CD  PSS   278      55.182  22.384  65.223
ATOM    246  OD  PSS   278      54.749  23.241  66.296
ATOM    247  CD1 PSS   278      53.447  22.889  66.875
ATOM    248  OC  PSS   278      54.603  22.985  64.099
ATOM    249  CA  PSS   278      56.824  23.996  63.420
ATOM    250  CP  PSS   278      55.290  24.111  63.604
ATOM    251  CB4 PSS   278      58.169  22.146  59.667
ATOM    252  OM  PSS   278      58.370  21.748  58.464
ATOM    253  CM  PSS   278      59.380  22.176  57.676
```

| | | | | | | | |
|---|---|---|---|---|---|---|---|
| ATOM | 254 | CB3 | PSS | 278 | 58.913 | 23.090 | 60.347 |
| ATOM | 255 | CB2 | PSS | 278 | 58.464 | 23.622 | 61.570 |
| ATOM | 256 | CB1 | PSS | 278 | 57.315 | 23.154 | 62.194 |
| ATOM | 257 | CB6 | PSS | 278 | 56.616 | 22.117 | 61.619 |
| ATOM | 258 | CB5 | PSS | 278 | 57.000 | 21.591 | 60.380 |
| ATOM | 259 | C | PSS | 278 | 54.671 | 24.724 | 62.262 |
| ATOM | 260 | O | PSS | 278 | 53.949 | 24.090 | 61.489 |
| ATOM | 261 | N | LEU | 279 | 54.887 | 26.033 | 62.120 |
| ATOM | 262 | CA | LEU | 279 | 54.183 | 26.931 | 61.202 |
| ATOM | 263 | CB | LEU | 279 | 53.216 | 27.660 | 62.200 |
| ATOM | 264 | CG | LEU | 279 | 52.423 | 28.857 | 61.644 |
| ATOM | 265 | CD1 | LEU | 279 | 51.597 | 28.363 | 60.471 |
| ATOM | 266 | CD2 | LEU | 279 | 51.388 | 29.443 | 62.604 |
| ATOM | 267 | C | LEU | 279 | 55.168 | 27.784 | 60.338 |
| ATOM | 268 | O | LEU | 279 | 56.162 | 28.295 | 60.802 |
| ATOM | 269 | N | VAL | 280 | 54.859 | 27.791 | 59.022 |
| ATOM | 270 | CA | VAL | 280 | 55.592 | 28.752 | 58.156 |
| ATOM | 271 | CB | VAL | 280 | 55.501 | 28.287 | 56.706 |
| ATOM | 272 | CG1 | VAL | 280 | 54.047 | 28.357 | 56.107 |
| ATOM | 273 | CG2 | VAL | 280 | 56.459 | 29.089 | 55.842 |
| ATOM | 274 | C | VAL | 280 | 55.086 | 30.211 | 58.441 |
| ATOM | 275 | O | VAL | 280 | 53.838 | 30.358 | 58.267 |
| ATOM | 276 | N | PSS | 281 | 55.931 | 31.156 | 58.828 |
| ATOM | 277 | CE | PSS | 281 | 57.381 | 30.941 | 59.027 |
| ATOM | 278 | CD | PSS | 281 | 58.300 | 32.060 | 58.609 |
| ATOM | 279 | OD | PSS | 281 | 58.378 | 32.178 | 57.193 |
| ATOM | 280 | CD1 | PSS | 281 | 59.251 | 33.293 | 56.691 |
| ATOM | 281 | OC | PSS | 281 | 57.828 | 33.318 | 58.953 |
| ATOM | 282 | CA | PSS | 281 | 55.431 | 32.501 | 59.201 |
| ATOM | 283 | CP | PSS | 281 | 56.398 | 33.467 | 58.455 |
| ATOM | 284 | CB4 | PSS | 281 | 54.864 | 32.837 | 63.521 |
| ATOM | 285 | OM | PSS | 281 | 54.700 | 33.062 | 64.820 |
| ATOM | 286 | CM | PSS | 281 | 53.494 | 32.762 | 65.525 |
| ATOM | 287 | CB3 | PSS | 281 | 53.789 | 32.546 | 62.622 |
| ATOM | 288 | CB2 | PSS | 281 | 54.006 | 32.403 | 61.248 |
| ATOM | 289 | CB1 | PSS | 281 | 55.293 | 32.561 | 60.754 |
| ATOM | 290 | CB6 | PSS | 281 | 56.317 | 32.805 | 61.685 |
| ATOM | 291 | CB5 | PSS | 281 | 56.092 | 33.097 | 63.011 |
| ATOM | 292 | C | PSS | 281 | 55.871 | 34.932 | 58.588 |
| ATOM | 293 | O | PSS | 281 | 55.184 | 35.347 | 57.629 |
| ATOM | 294 | N | LEU | 282 | 56.073 | 35.637 | 59.696 |
| ATOM | 295 | CA | LEU | 282 | 55.611 | 36.947 | 59.967 |
| ATOM | 296 | CB | LEU | 282 | 55.916 | 37.345 | 61.431 |
| ATOM | 297 | CG | LEU | 282 | 55.113 | 36.547 | 62.517 |
| ATOM | 298 | CD1 | LEU | 282 | 55.730 | 36.498 | 63.891 |
| ATOM | 299 | CD2 | LEU | 282 | 53.640 | 37.144 | 62.641 |
| ATOM | 300 | C | LEU | 282 | 56.178 | 37.992 | 58.958 |
| ATOM | 301 | O | LEU | 282 | 55.636 | 39.073 | 58.964 |
| ATOM | 302 | N | VAL | 283 | 57.314 | 37.670 | 58.330 |
| ATOM | 303 | CA | VAL | 283 | 57.993 | 38.399 | 57.270 |
| ATOM | 304 | CB | VAL | 283 | 59.325 | 37.748 | 56.913 |
| ATOM | 305 | CG1 | VAL | 283 | 60.097 | 38.513 | 55.756 |
| ATOM | 306 | CG2 | VAL | 283 | 60.277 | 37.668 | 58.156 |
| ATOM | 307 | C | VAL | 283 | 57.052 | 38.674 | 56.058 |
| ATOM | 308 | O | VAL | 283 | 56.949 | 37.824 | 55.107 |
| ATOM | 309 | OS | BZN | 284 | 56.286 | 39.753 | 56.146 |
| ATOM | 310 | CB | BZN | 284 | 55.125 | 39.849 | 55.323 |
| ATOM | 311 | CG | BZN | 284 | 54.457 | 41.160 | 55.620 |
| ATOM | 312 | CD1 | BZN | 284 | 53.375 | 41.178 | 56.493 |
| ATOM | 313 | CE1 | BZN | 284 | 52.572 | 42.365 | 56.496 |
| ATOM | 314 | CZ | BZN | 284 | 52.749 | 43.374 | 55.558 |
| ATOM | 315 | CE2 | BZN | 284 | 53.808 | 43.363 | 54.659 |
| ATOM | 316 | CD2 | BZN | 284 | 54.735 | 42.307 | 54.764 |
| TER | 317 | | BZN | 284 | | | |

**Representative geometry of cluster c0 for HPP:L-Pro$_{10}$ (binding site residues up to 4.5 Å to the ligand are reported; hydrogen atoms are omitted).**

| | | | | | | | |
|---|---|---|---|---|---|---|---|
| ATOM | 1 | N | TRP | 2 | 61.116 | 34.349 | 63.747 |
| ATOM | 2 | CA | TRP | 2 | 60.399 | 34.096 | 65.030 |
| ATOM | 3 | CB | TRP | 2 | 60.210 | 32.610 | 65.385 |
| ATOM | 4 | CG | TRP | 2 | 58.996 | 32.122 | 64.626 |
| ATOM | 5 | CD1 | TRP | 2 | 58.998 | 31.092 | 63.772 |
| ATOM | 6 | NE1 | TRP | 2 | 57.661 | 30.879 | 63.327 |
| ATOM | 7 | CE2 | TRP | 2 | 56.835 | 31.824 | 63.859 |
| ATOM | 8 | CZ2 | TRP | 2 | 55.506 | 32.195 | 63.527 |
| ATOM | 9 | CH2 | TRP | 2 | 54.872 | 33.208 | 64.302 |
| ATOM | 10 | CZ3 | TRP | 2 | 55.615 | 33.904 | 65.323 |
| ATOM | 11 | CE3 | TRP | 2 | 56.998 | 33.598 | 65.504 |
| ATOM | 12 | CD2 | TRP | 2 | 57.613 | 32.582 | 64.733 |
| ATOM | 13 | C | TRP | 2 | 61.082 | 34.875 | 66.216 |
| ATOM | 14 | O | TRP | 2 | 60.382 | 35.258 | 67.149 |
| ATOM | 15 | N | ALA | 4 | 62.489 | 38.119 | 66.090 |
| ATOM | 16 | CA | ALA | 4 | 62.104 | 39.531 | 65.976 |
| ATOM | 17 | CB | ALA | 4 | 61.816 | 39.843 | 64.535 |
| ATOM | 18 | C | ALA | 4 | 60.873 | 40.028 | 66.762 |
| ATOM | 19 | O | ALA | 4 | 60.872 | 41.190 | 67.166 |
| ATOM | 20 | N | TYR | 5 | 59.911 | 39.207 | 67.032 |
| ATOM | 21 | CA | TYR | 5 | 58.851 | 39.391 | 68.006 |
| ATOM | 22 | CB | TYR | 5 | 57.723 | 38.450 | 67.856 |
| ATOM | 23 | CG | TYR | 5 | 57.300 | 38.299 | 66.394 |
| ATOM | 24 | CD1 | TYR | 5 | 57.832 | 37.297 | 65.508 |
| ATOM | 25 | CE1 | TYR | 5 | 57.559 | 37.274 | 64.102 |
| ATOM | 26 | CZ | TYR | 5 | 56.609 | 38.157 | 63.631 |
| ATOM | 27 | OH | TYR | 5 | 56.300 | 38.237 | 62.324 |
| ATOM | 28 | CE2 | TYR | 5 | 55.965 | 39.072 | 64.461 |
| ATOM | 29 | CD2 | TYR | 5 | 56.287 | 39.100 | 65.825 |
| ATOM | 30 | C | TYR | 5 | 59.290 | 39.472 | 69.439 |
| ATOM | 31 | O | TYR | 5 | 58.987 | 40.395 | 70.154 |
| ATOM | 32 | N | ASN | 8 | 60.860 | 42.858 | 69.959 |
| ATOM | 33 | CA | ASN | 8 | 59.829 | 43.824 | 70.299 |
| ATOM | 34 | CB | ASN | 8 | 58.689 | 43.665 | 69.350 |
| ATOM | 35 | CG | ASN | 8 | 57.669 | 44.640 | 69.503 |
| ATOM | 36 | OD1 | ASN | 8 | 56.733 | 44.363 | 70.182 |
| ATOM | 37 | ND2 | ASN | 8 | 57.595 | 45.861 | 68.968 |
| ATOM | 38 | C | ASN | 8 | 59.360 | 43.707 | 71.788 |
| ATOM | 39 | O | ASN | 8 | 59.307 | 44.715 | 72.445 |
| ATOM | 40 | N | ASP | 25 | 47.277 | 20.475 | 67.849 |
| ATOM | 41 | CA | ASP | 25 | 47.666 | 19.143 | 67.523 |
| ATOM | 42 | CB | ASP | 25 | 48.056 | 18.956 | 66.045 |
| ATOM | 43 | CG | ASP | 25 | 46.957 | 19.268 | 65.028 |
| ATOM | 44 | OD1 | ASP | 25 | 45.867 | 18.684 | 65.177 |
| ATOM | 45 | OD2 | ASP | 25 | 47.257 | 19.931 | 64.047 |
| ATOM | 46 | C | ASP | 25 | 48.802 | 18.552 | 68.343 |
| ATOM | 47 | O | ASP | 25 | 48.846 | 17.353 | 68.646 |
| ATOM | 48 | N | TRP | 30 | 58.366 | 27.975 | 69.733 |
| ATOM | 49 | CA | TRP | 30 | 58.655 | 28.727 | 68.456 |
| ATOM | 50 | CB | TRP | 30 | 57.436 | 29.343 | 67.819 |
| ATOM | 51 | CG | TRP | 30 | 56.321 | 28.409 | 67.458 |
| ATOM | 52 | CD1 | TRP | 30 | 56.213 | 27.064 | 67.652 |
| ATOM | 53 | NE1 | TRP | 30 | 54.958 | 26.705 | 67.318 |
| ATOM | 54 | CE2 | TRP | 30 | 54.109 | 27.768 | 66.906 |

```
ATOM   55  CZ2 TRP   30     52.765  27.926  66.482
ATOM   56  CH2 TRP   30     52.319  29.172  66.008
ATOM   57  CZ3 TRP   30     53.200  30.298  66.043
ATOM   58  CE3 TRP   30     54.604  30.140  66.527
ATOM   59  CD2 TRP   30     55.055  28.905  66.896
ATOM   60  C   TRP   30    59.911  29.626  68.542
ATOM   61  O   TRP   30    60.898  29.592  67.831
ATOM   62  N   LYS  106    45.882  29.471  67.234
ATOM   63  CA  LYS  106    47.025  28.605  66.917
ATOM   64  CB  LYS  106    47.298  28.489  65.371
ATOM   65  CG  LYS  106    45.981  28.121  64.618
ATOM   66  CD  LYS  106    46.134  28.094  63.097
ATOM   67  CE  LYS  106    44.835  27.489  62.512
ATOM   68  NZ  LYS  106    44.861  27.291  61.077
ATOM   69  C   LYS  106    48.310  28.861  67.681
ATOM   70  O   LYS  106    49.224  28.111  67.541
ATOM   71  N   HIE  132    48.645  40.988  66.972
ATOM   72  CA  HIE  132    49.559  41.323  65.828
ATOM   73  CB  HIE  132    51.050  41.604  66.328
ATOM   74  CG  HIE  132    52.067  41.961  65.239
ATOM   75  ND1 HIE  132    52.095  43.231  64.660
ATOM   76  CE1 HIE  132    52.885  43.202  63.595
ATOM   77  NE2 HIE  132    53.399  41.949  63.493
ATOM   78  CD2 HIE  132    52.856  41.124  64.490
ATOM   79  C   HIE  132    49.533  40.061  64.946
ATOM   80  O   HIE  132    49.255  40.149  63.764
ATOM   81  N   LEU  133    49.824  38.877  65.508
ATOM   82  CA  LEU  133    49.711  37.639  64.742
ATOM   83  CB  LEU  133    50.389  36.495  65.644
ATOM   84  CG  LEU  133    51.876  36.838  66.035
ATOM   85  CD1 LEU  133    52.501  35.770  66.974
ATOM   86  CD2 LEU  133    52.815  36.999  64.839
ATOM   87  C   LEU  133    48.269  37.247  64.400
ATOM   88  O   LEU  133    48.026  36.548  63.413
ATOM   89  N   SER  136    47.307  38.600  61.050
ATOM   90  CA  SER  136    47.876  37.657  60.053
ATOM   91  CB  SER  136    49.447  37.612  60.340
ATOM   92  OG  SER  136    49.966  38.937  60.272
ATOM   93  C   SER  136    47.273  36.210  60.045
ATOM   94  O   SER  136    47.908  35.375  59.526
ATOM   95  N   GLN  137    46.056  36.094  60.625
ATOM   96  CA  GLN  137    45.228  34.951  60.640
ATOM   97  CB  GLN  137    44.767  34.599  59.212
ATOM   98  CG  GLN  137    43.951  35.801  58.577
ATOM   99  CD  GLN  137    42.655  36.173  59.252
ATOM  100  OE1 GLN  137    41.831  35.332  59.611
ATOM  101  NE2 GLN  137    42.371  37.392  59.510
ATOM  102  C   GLN  137    45.851  33.741  61.392
ATOM  103  O   GLN  137    45.850  32.573  60.960
ATOM  104  N   TYR  138    46.682  34.047  62.439
ATOM  105  CA  TYR  138    46.865  32.997  63.434
ATOM  106  CB  TYR  138    48.199  33.211  64.134
ATOM  107  CG  TYR  138    49.437  32.845  63.358
ATOM  108  CD1 TYR  138    50.309  31.878  63.854
ATOM  109  CE1 TYR  138    51.486  31.508  63.210
ATOM  110  CZ  TYR  138    51.725  32.134  61.955
ATOM  111  OH  TYR  138    52.804  31.747  61.216
ATOM  112  CE2 TYR  138    50.965  33.226  61.440
ATOM  113  CD2 TYR  138    49.840  33.538  62.214
ATOM  114  C   TYR  138    45.685  32.827  64.421
ATOM  115  O   TYR  138    45.810  31.923  65.322
ATOM  116  OXT TYR  138    44.530  33.338  64.258
TER   117      TYR  138
ATOM  118  N   ALA  142    52.016  30.566  49.246
ATOM  119  CA  ALA  142    52.404  31.603  50.222
ATOM  120  CB  ALA  142    51.271  31.630  51.246
ATOM  121  C   ALA  142    53.771  31.426  50.855
ATOM  122  O   ALA  142    54.496  32.350  51.193
ATOM  123  N   TYR  143    54.159  30.137  51.002
ATOM  124  CA  TYR  143    55.486  29.774  51.523
ATOM  125  CB  TYR  143    55.510  28.345  51.932
ATOM  126  CG  TYR  143    54.608  27.837  52.986
ATOM  127  CD1 TYR  143    53.250  27.599  52.727
ATOM  128  CE1 TYR  143    52.478  26.897  53.618
ATOM  129  CZ  TYR  143    53.079  26.460  54.836
ATOM  130  OH  TYR  143    52.358  25.678  55.677
ATOM  131  CE2 TYR  143    54.425  26.720  55.139
ATOM  132  CD2 TYR  143    55.172  27.434  54.185
ATOM  133  C   TYR  143    56.650  30.066  50.587
ATOM  134  O   TYR  143    57.666  30.657  50.935
ATOM  135  N   ASN  146    56.858  33.884  50.808
ATOM  136  CA  ASN  146    57.548  34.310  52.018
ATOM  137  CB  ASN  146    57.099  33.469  53.171
ATOM  138  CG  ASN  146    57.595  33.900  54.568
ATOM  139  OD1 ASN  146    58.014  33.103  55.351
ATOM  140  ND2 ASN  146    57.586  35.184  54.834
ATOM  141  C   ASN  146    59.081  34.221  51.963
ATOM  142  O   ASN  146    59.790  35.062  52.517
ATOM  143  N   HIE  270    59.871  25.922  60.361
ATOM  144  CA  HIE  270    58.434  26.277  60.228
ATOM  145  CB  HIE  270    58.511  27.627  59.398
ATOM  146  CG  HIE  270    57.151  28.237  59.741
ATOM  147  ND1 HIE  270    56.833  28.787  60.993
ATOM  148  CE1 HIE  270    55.533  29.198  60.866
ATOM  149  NE2 HIE  270    55.028  28.928  59.611
ATOM  150  CD2 HIE  270    56.041  28.407  58.859
ATOM  151  C   HIE  270    57.483  25.174  59.670
ATOM  152  O   HIE  270    56.348  25.052  60.118
ATOM  153  N   SER  274    54.607  23.204  62.492
ATOM  154  CA  SER  274    53.229  23.155  62.043
ATOM  155  CB  SER  274    53.156  23.723  60.639
ATOM  156  OG  SER  274    53.732  25.010  60.475
ATOM  157  C   SER  274    52.625  21.751  61.996
ATOM  158  O   SER  274    51.495  21.600  61.534
ATOM  159  N   TYR  276    52.980  18.683  60.424
ATOM  160  CA  TYR  276    52.821  17.864  59.218
ATOM  161  CB  TYR  276    53.292  18.574  58.000
ATOM  162  CG  TYR  276    52.628  19.829  57.554
ATOM  163  CD1 TYR  276    53.386  21.011  57.354
ATOM  164  CE1 TYR  276    52.731  22.216  57.005
ATOM  165  CZ  TYR  276    51.324  22.239  56.794
ATOM  166  OH  TYR  276    50.686  23.418  56.471
ATOM  167  CE2 TYR  276    50.634  21.002  56.922
ATOM  168  CD2 TYR  276    51.287  19.826  57.407
ATOM  169  C   TYR  276    53.461  16.496  59.445
ATOM  170  O   TYR  276    52.825  15.513  58.988
ATOM  171  OXT TYR  276    54.512  16.302  60.013
TER   172      TYR  276
ATOM  173  N   PRO  277    57.334  48.907  63.797
ATOM  174  CD  PRO  277    56.409  49.924  63.242
ATOM  175  CG  PRO  277    55.172  49.194  62.602
ATOM  176  CB  PRO  277    55.554  47.702  62.879
```

| | | | | | | | |
|---|---|---|---|---|---|---|---|
| ATOM | 177 | CA | PRO | 277 | 56.484 | 47.690 | 63.982 |
| ATOM | 178 | C | PRO | 277 | 57.311 | 46.411 | 64.013 |
| ATOM | 179 | O | PRO | 277 | 58.304 | 46.427 | 63.232 |
| ATOM | 180 | N | PRO | 278 | 56.957 | 45.339 | 64.699 |
| ATOM | 181 | CD | PRO | 278 | 55.937 | 45.241 | 65.667 |
| ATOM | 182 | CG | PRO | 278 | 55.542 | 43.736 | 65.812 |
| ATOM | 183 | CB | PRO | 278 | 56.895 | 43.083 | 65.600 |
| ATOM | 184 | CA | PRO | 278 | 57.448 | 43.922 | 64.482 |
| ATOM | 185 | C | PRO | 278 | 57.125 | 43.405 | 63.035 |
| ATOM | 186 | O | PRO | 278 | 56.146 | 43.822 | 62.417 |
| ATOM | 187 | N | PRO | 279 | 57.888 | 42.458 | 62.428 |
| ATOM | 188 | CD | PRO | 279 | 59.126 | 41.965 | 63.101 |
| ATOM | 189 | CG | PRO | 279 | 59.684 | 40.858 | 62.218 |
| ATOM | 190 | CB | PRO | 279 | 59.118 | 41.180 | 60.838 |
| ATOM | 191 | CA | PRO | 279 | 57.781 | 41.943 | 61.059 |
| ATOM | 192 | C | PRO | 279 | 56.502 | 41.135 | 60.785 |
| ATOM | 193 | O | PRO | 279 | 55.835 | 40.613 | 61.688 |
| ATOM | 194 | N | PRO | 280 | 56.208 | 40.870 | 59.506 |
| ATOM | 195 | CD | PRO | 280 | 56.928 | 41.389 | 58.359 |
| ATOM | 196 | CG | PRO | 280 | 56.318 | 40.690 | 57.112 |
| ATOM | 197 | CB | PRO | 280 | 54.928 | 40.245 | 57.648 |
| ATOM | 198 | CA | PRO | 280 | 55.130 | 39.956 | 59.130 |
| ATOM | 199 | C | PRO | 280 | 55.430 | 38.454 | 59.333 |
| ATOM | 200 | O | PRO | 280 | 56.472 | 38.013 | 58.953 |
| ATOM | 201 | N | PRO | 281 | 54.508 | 37.616 | 59.859 |
| ATOM | 202 | CD | PRO | 281 | 53.338 | 38.102 | 60.470 |
| ATOM | 203 | CG | PRO | 281 | 52.654 | 36.879 | 61.016 |
| ATOM | 204 | CB | PRO | 281 | 53.752 | 35.784 | 61.115 |
| ATOM | 205 | CA | PRO | 281 | 54.744 | 36.229 | 60.002 |
| ATOM | 206 | C | PRO | 281 | 54.725 | 35.433 | 58.611 |
| ATOM | 207 | O | PRO | 281 | 54.103 | 35.842 | 57.650 |
| ATOM | 208 | N | PRO | 282 | 55.402 | 34.233 | 58.629 |
| ATOM | 209 | CD | PRO | 282 | 56.400 | 33.672 | 59.549 |
| ATOM | 210 | CG | PRO | 282 | 56.270 | 32.156 | 59.521 |
| ATOM | 211 | CB | PRO | 282 | 55.926 | 31.979 | 58.016 |
| ATOM | 212 | CA | PRO | 282 | 55.009 | 33.166 | 57.713 |
| ATOM | 213 | C | PRO | 282 | 53.558 | 32.655 | 57.787 |
| ATOM | 214 | O | PRO | 282 | 52.873 | 32.881 | 58.789 |
| ATOM | 215 | N | PRO | 283 | 53.165 | 31.718 | 56.869 |
| ATOM | 216 | CD | PRO | 283 | 53.772 | 31.345 | 55.623 |
| ATOM | 217 | CG | PRO | 283 | 52.635 | 30.939 | 54.727 |
| ATOM | 218 | CB | PRO | 283 | 51.467 | 30.528 | 55.607 |
| ATOM | 219 | CA | PRO | 283 | 51.741 | 31.257 | 56.876 |
| ATOM | 220 | C | PRO | 283 | 51.420 | 30.405 | 58.122 |
| ATOM | 221 | O | PRO | 283 | 52.384 | 29.772 | 58.639 |
| ATOM | 222 | N | PRO | 284 | 50.198 | 30.369 | 58.657 |
| ATOM | 223 | CD | PRO | 284 | 49.101 | 31.134 | 58.156 |
| ATOM | 224 | CG | PRO | 284 | 47.909 | 30.695 | 59.049 |
| ATOM | 225 | CB | PRO | 284 | 48.506 | 30.328 | 60.410 |
| ATOM | 226 | CA | PRO | 284 | 49.904 | 29.774 | 59.969 |
| ATOM | 227 | C | PRO | 284 | 49.820 | 28.209 | 59.856 |
| ATOM | 228 | O | PRO | 284 | 49.648 | 27.739 | 58.739 |
| ATOM | 229 | N | PRO | 285 | 49.958 | 27.429 | 60.944 |
| ATOM | 230 | CD | PRO | 285 | 50.156 | 27.863 | 62.313 |
| ATOM | 231 | CG | PRO | 285 | 50.740 | 26.714 | 63.039 |
| ATOM | 232 | CB | PRO | 285 | 49.981 | 25.569 | 62.403 |
| ATOM | 233 | CA | PRO | 285 | 49.750 | 25.953 | 60.910 |
| ATOM | 234 | C | PRO | 285 | 48.255 | 25.511 | 60.536 |
| ATOM | 235 | O | PRO | 285 | 47.422 | 26.372 | 60.639 |
| ATOM | 236 | N | PRO | 286 | 47.977 | 24.242 | 60.131 |
| ATOM | 237 | CD | PRO | 286 | 48.938 | 23.356 | 59.604 |
| ATOM | 238 | CG | PRO | 286 | 48.122 | 22.456 | 58.734 |
| ATOM | 239 | CB | PRO | 286 | 46.851 | 22.186 | 59.525 |
| ATOM | 240 | CA | PRO | 286 | 46.620 | 23.655 | 59.872 |
| ATOM | 241 | C | PRO | 286 | 45.503 | 23.797 | 60.989 |
| ATOM | 242 | O | PRO | 286 | 44.450 | 24.371 | 60.650 |
| ATOM | 243 | OXT | PRO | 286 | 45.664 | 23.266 | 62.149 |
| TER | 244 | | PRO | 286 | | | |

# 2. Synthesis of compounds 2-4,7-9,11,12

*General information.* Chemicals were purchased from Sigma Aldrich and were used without further purification. Mass spectra were recorded on an LCQESI MS and on a LCQ Advantage spectrometer from Thermo Finningan and a LCQ Fleet spectrometer from Thermo Scientific. The NMR spectroscopic experiments were carried out either on Varian MERCURY 300 MHz (300 and 75 MHz for $^{1}H$ and $^{13}C$, respectively), or Bruker Avance I 500 MHz spectrometers (500 and 125 MHz for $^{1}H$ and $^{13}C$, respectively). Optical rotations were measured on a Perkin-Elmer 343 polarimeter at 20 °C (concentration in g/100 mL). Chemical shifts ($\delta$) are given in ppm relative to the $CHCl_3$ internal standard, and the coupling constants $J$ are reported in Hertz (Hz). The synthesis of dipeptide **10**[1] and compound **1**[1] are reported in the literature.

*(2S,6S)-4-Boc-6-methoxy-N-(quinolin-7-yl)-morpholine-2-carboxyamide (+)-(7). Method A.* To a solution of acid **1** (615 mg, 2.35 mmol, 1 equiv.) in anhydrous $CH_2Cl_2$ (25 mL), EDCI·HCl (402.4 mg, 2.6 mmol, 1.1 equiv.) and DMAP (57.57 mg, 0.471 mmol, 0.2 equiv.) were added at 0 °C. The

mixture was stirred for 1 h then 8-aminoquinoline (373.7 mg, 2.6 mmol, 1.1 equiv.) was added. The reaction mixture was stirred for 24 h at 25 °C. The organic layer was washed with a solution of $KHSO_4$ (5%, 25 mL), a saturated solution of $NaHCO_3$ (25 mL) and brine (25 mL). After drying over $Na_2SO_4$, the solvent was removed under reduced pressure. Purification of the crude product by silica gel flash chromatography (*n*hexane/AcOEt 8:2) afforded the amide **7** (385 mg, 0.99 mmol, 43%) as colorless oil. *Method B.* To a solution of compound **1** (277 mg, 1.06 mmol, 1 equiv.) in anhydrous $CH_2Cl_2$ (15 mL) at 0 °C, a propylphosphonic anhydride (T3P) (2.65 mmol, 2.5 equiv. 1.7 mL of 50% DMF solution), 8-aminoquinoline (168.23 mg, 1.16 mmol, 1.1 equiv.) and DIPEA (647 µL, 3.71 mmol, 3.5 equiv.) were added. The reaction mixture was stirred for 24 h at 25 °C. The crude was elaborated as reported in *Method A* and then purified by chromatography. Compound **7** was isolated as colorless oil in 81% (334 mg, 0.86 mmol). $^1$H NMR ($CDCl_3$, 300 MHz) $\delta$ 10.81 (s, 1H), 8.88 (dd, *J* = 4.2, 1.6 Hz, 1H), 8.84-8.80 (m, 1H), 8.20 (dd, *J* = 8.3, 1.6 Hz, 1H), 7.57 (d, *J* = 4.5 Hz, 2H), 7.49 (dd, *J* = 8.3, 4.2 Hz, 1H), 5.01 (brs, 1H), 4.66 (dd, *J* = 10.8, 3.3 Hz, 1H), 4.47 (brs, 1H), 4.08 (brs, 1H), 3.53 (s, 3H), 3.25-2.97 (m, 2H), 1.51 (s, 9H); $^{13}$C NMR (75 MHz, $CDCl_3$) $\delta$ 167.8, 154.9, 148.4, 138.7, 136.0, 133.6, 128.1, 127.4, 122.3, 121.0, 117.3, 96.8, 77.5, 69.3, 55.3, 46.0, 45.3, 28.4 (x3); HRMS (ESI-TOF) m/z: $[M+Na]^+$ Calcd for $C_{20}H_{25}N_3O_5Na$ 410.1692; Found 410.1697. Anal. Calcd for $C_{20}H_{25}N_3O_5$: C, 62.00; H, 6.50; N, 10.85. Found C, 61.88; H, 6.60; N, 10.79. $[\alpha]_D^{20}$ = +43.6 (c 0.2 in $CHCl_3$).

*(2S,3S,6S)-4-Boc-6-methoxy-3-(4-methoxyphenyl)-N-(quinolin-7-yl)-morpholine-2-carboxyamide (+)-(**8**).* Operating in a sealed tube amide **7** (1.4 g, 3.4 mmol, 1.0 equiv.) was dissolved in toluene (65 mL) and AgOAc (1.13 g, 6.8 mmol, 2.0 equiv.), 4-iodoanisole (2.4 g, 10.2 mmol, 3.0 equiv.), $Pd(OAc)_2$ (305.3 mg, 1.36 mmol, 0.4 equiv.) were added. The tube was flushed with argon and sealed, then placed in a preheated oil bath to 110 °C (oil bath) and stirred for 38 h. The reaction mixture was cooled at 25 °C and EtOAc (10 mL) was added. The resulting solution was filtered through a Celite®pad, that was washed with EtOAc (10 mL). The solvent was removed in vacuo, and the crude material was purified by flash column chromatography (*n*hexane/AcOEt, 8:2) affording pure compound **8** as a yellow oil (606 mg, 0.86 mmol, 37%). $^1$H NMR (300 MHz, $CDCl_3$) δ 10.84 (s, 1H), 8.83-8.66 (m, 2H), 8.19 (dd, *J* = 8.3, 1.6 Hz, 1H), 7.56-7.41 (m, 3H), 7.47, 6.88 (AA'XX' system, *J* = 8.8 Hz, 4H), 5.59 (d, *J* = 6.3 Hz, 1H), 5.12 (dd, *J* = 8.5, 5.2 Hz, 1H), 4.90 (d, *J* = 6.3 Hz, 1H), 4.33 (dd, *J* = 14.4, 5.2 Hz, 1H), 3.82 (s, 3H), 3.72 (s, 3H), 3.04 (dd, *J* = 14.4, 8.5 Hz, 1H), 1.43 (s, 9H); $^{13}$C NMR (75 MHz, $CDCl_3$) δ 167.7, 159.5, 155.2, 148.7, 139.1, 136.7, 134.3, 131.7, 129.3(x2), 128.4, 127.7, 122.4, 122.1, 117.1, 114.2(x2), 98.5, 81.1, 74.2, 56.6, 55.8, 55.6, 42.2, 28.7(x3); HRMS (ESI-TOF): m/z $[M+H]^+$ Calcd for $C_{27}H_{32}N_3O_6$ 494.2291; Found 494.2294. Anal. Calcd for

$C_{27}H_{31}N_3O$: C, 65.71; H, 6.33; N, 8.51. Found C, 65.49; H, 6.49; N, 8.39. $[\alpha]_D^{20}$ = +33.6 (c 0.3 in $CHCl_3$).

*(2S,3S,6S)-4-Boc-6-methoxy-3-(4-methoxyphenyl)-N-Boc-N'-(quinolin-7-yl)-morpholine-2-carboxyamide (+)-(**9**).* Compound **8** (606 mg, 1.22 mmol, 1.0 equiv.) was dissolved in MeCN (30 mL). DMAP (413.4 mg, 3.68 mmol, 3.0 equiv.) and $(Boc)_2O$ (5.3 g, 24.5 mol 20.0 equiv.) were added at 25 °C. The reaction mixture was stirred at 70 °C (oil bath) for 6 h. After cooling at 25 °C, the reaction mixture was concentrated in vacuo. The crude mixture was purified by flash chromatography (*n*hexane/AcOEt, 6:4) affording pure compound **9** as an oil (604 mg, 1.01 mmol, 83%). $^1H$ NMR (300 MHz, $CDCl_3$) δ 8.90 (dd, *J* = 4.1, 1.6 Hz, 1H), 8.19 (dd, *J* = 8.5, 1.6 Hz, 1H), 7.87-7.82 (m, 1H), 7.60-7.47 (m, 4H), 7.43 (dd, *J* = 8.4, 4.4 Hz, 1H), 6.92 (d, *J* = 8.9 Hz, 2H), 6.14 (brs, 1H), 5.74 (brs, 1H), 5.39 (brs, 1H), 4.16-4.03 (m, 1H), 3.84 (s, 3H), 3.53 (s, 3H), 2.85 (dd, *J* = 13.6, 9.4 Hz, 1H), 1.45 (s, 9H), 1.23 (s, 9H); $^{13}C$ NMR (75 MHz, $CDCl_3$) δ 159.4, 155.1, 152.8, 150.8, 144.6, 137.1, 136.4, 131.3, 129.4(x2), 129.3, 129.1, 128.7, 126.5, 121.9, 114.2(x2), 97.4, 83.8, 80.8, 75.9, 56.8, 55.7, 54.6, 42.4, 28.7(x3), 27.9(x3); HRMS (ESI-TOF): m/z $[M+Na]^+$ Calcd for $C_{32}H_{39}N_3O_8Na$ 616.2635; Found 616.2639 Anal. Calcd for $C_{32}H_{39}N_3O_8$: C, 64.74; H, 6.62; N, 7.08. Found: C, 64.63; H, 6.70; N, 7.00. $[\alpha]_D^{20}$ = +25.8 (c 0.2 in $CHCl_3$).

*(2S,3S,6S)-4-Boc-6-methoxy-3-(4-methoxyphenyl)-2-carboxylic acid (-)-(**2**).* A solution of compound **9** (450 mg, 0.758 mmol, 1.0 equiv.) in THF/$H_2O$ (10 mL, 3:1) was cooled at 0 °C. LiOH $H_2O$ (63.6 mg, 1.51 mmol, 2 equiv.) and $H_2O_2$ (35%, 128.9 mg, 3.79 mmol, 5 equiv.) were added and the reaction mixture was stirred for 20 min at 0 °C. After warming at 25 °C, the stirring was continued for 18 h. The reaction was extracted with $Et_2O$ to remove the organic impurities and then the aqueous layer was acidified with 1 M HCl to pH = 6 and extracted with EtOAc (3 x 10 mL). The combined organic layers were washed with brine (3 x 10 mL), dried over $Na_2SO_4$. The solvent was removed and the acid **2** was obtained as an oil and was used without further purification (271.6 mg 0.74 mmol, 98%). Detailed NMR data are reported in Table TS3. HRMS (ESI-TOF): m/z $[M+Na]^+$ Calcd for $C_{18}H_{25}NO_7Na$ 390.1529; Found 390.1532. Anal. Calcd for $C_{18}H_{25}NO_7$: C, 58.85; H, 6.86; N, 3.81. Found: C, 58.63; H, 6.90; N, 3.65. $[\alpha]_D^{20}$ = -12.24 (c 1.24 in $CHCl_3$).

*N-Boc-(-)-3-Ar-β-Morph-L-Leu-L-Val-OBn (-)-(**3**). Method A.* Operating in a round-bottom flask equipped with a magnetic stirrer and thermometer, acid **2** (25 mg, 0.068 mmol, 1 equiv.) was dissolved in $CH_2Cl_2$ (2 mL). The solution was cooled to 0 °C. HOBt (10.1 mg, 0.074 mmol, 1.1 equiv.) and EDC (11.6 mg, 0.074 mmol, 1.1 equiv.) were added. After 1 h, TFA·$NH_2$-*L*-Leu-*L*-Val-OBn (**10**) (21.8 mg, 0.068 mmol, 1.1 equiv.) and DIPEA (24.8 µL, 0.142 mmol, 2.1 equiv.) were added and stirring was continued for 24 h at 25 °C. The organic layer was washed with a solution of $KHSO_4$ (5%, 5 mL), a saturated solution of $NaHCO_3$ (5 mL) and brine (5 mL). After drying over

$Na_2SO_4$, the solvent was removed under reduced pressure. Purification of the crude product by silica gel flash chromatography (*n*hexane/AcOEt, 7:3) afforded the corresponding tripeptide **3** as a colorless oil (30 mg, 0.044 mmol, 65 %). *Method B.* To a solution of acid **2** (50 mg, 0.13 mmol, 1 equiv.) in $CH_2Cl_2$ (2.5 mL) at 0 °C, propylphosphonic anhydride solution (T3P) (50% solution in DMF, 215 µL, 0.34 mmol, 2.5 equiv.), dipeptide **10** (65 mg, 0.15 mmol, 1.1 equiv.) and DIPEA (71.1 µL, 0.40 mmol, 3.5 equiv.) were added. The reaction mixture was stirred for 24 h at 25 °C and then treated as reported in *Method A*. Purification of the crude product by silica gel flash chromatography (*n*hexane/AcOEt, 7:3) afforded tripeptide **3** as a colorless oil (67.5 mg, 0,1 mmol, 81%). Detailed NMR data are reported in Table TS4. HRMS (ESI-TOF): m/z $[M+Na]^+$ Calcd for $C_{36}H_{51}N_3O_9Na$ 692.3523; Found 692.3528.$[\alpha]_D^{20}$ = -24.13 (c 0.7 in $CHCl_3$).

*$CF_3CO_2H$ ·$NH_2$(-)-3-Ar-β-Morph-L-Leu-L-Val-OBn (+)-(**11**).* Operating in a round-bottom flask equipped with magnetic stirrer, compound **3** (26 mg, 0.04 mmol) was dissolved in $CH_2Cl_2$ (2 mL). The solution was cooled to 0 °C and TFA (2 mL) was slowly dropped. The solution was stirred at 25 °C for 2 h. The solvent was removed under reducing pressure affording compound **11** as $CF_3CO_2H$ salt, obtained in quantitative yield (23.3 mg) that was used without further purification. $^1H$ NMR (300 MHz, $CDCl_3$) δ 7.33 (m, 7H), 6.91 (d, *J* = 7.4 Hz, 1H), 6.83 (s brs, 2H), 6.30 (d, *J* = 8.5 Hz, 1H), 5.13 (dd, *J* = 25.7, 12.2 Hz, 2H), 4.80 (m, 2H), 4.44 (dd, *J* = 8.5, 4.9 Hz, 1H), 4.29 (s brs, 1H), 4.15 (s brs, 1H), 3.76 (s, 3H), 3.45 (s, 3H), 3.10 (s, brs 1H), 2.82 (s brs, 1H), 2.09 (m, 1H), 1.66-1.39 (m, 3H), 0.96 -0.61 (m, 12H); $^{13}C$ NMR (75 MHz, $CDCl_3$) δ 171.6, 166.5, 161.0, 135.6, 130.6, 129.0, 128.9, 128.8, 123.8, 114.6, 94.3, 70.7, 67.5, 60.5, 57.6, 56.0, 55.6, 51.7, 45.9, 41.0, 31.4, 30.0, 25.0, 23.1, 22.4, 19.2, 17.9. $[\alpha]_D^{20}$ = +22.5 (c 0.32 in $CHCl_3$).

*N-Boc-(-)-3-Ar-β-Morph-L-Leu-L-Val-OH (-)-(**12**).* Operating in a round-bottom flask equipped with a magnetic stirrer, compound **3** (52 mg, 0.077 mmol) was dissolved in THF (5 mL) and Pd/C (50 mg, 10% loading) was added to the solution. The suspension was stirred under $H_2$ (1 atm) at 25 °C for 2 h. The catalyst was filtered over a Celite pad. The solvent was removed under reduced pressure and the obtained clear oil was dissolved in $CH_2Cl_2$ (20 mL) and washed with a saturated solution of $NaHCO_3$ (20 mL). The aqueous layer was then acidified with 37% HCl until pH 2. The product was extracted with $CH_2Cl_2$ (2 x 20 mL). The organic layer was concentrated under vacuum, affording compound **12** (41.4 mg, 0.071 mmol, 93%) as colourless oil. $^1H$ NMR (300 MHz, $CDCl_3$) δ 7.29-7.26 (m, 2H), 6.99 (d, *J* = 8.9 Hz, 1H), 6.92-6.77 (m, 3H), 5.13 (d, *J* = 8.1 Hz, 1H), 4.91 (dd, *J* = 7.8, 5.9 Hz, 1H), 4.60 (m, 1H), 4.50 (m, 2H), 4.30 (dd, *J* = 14.1, 5.7 Hz, 1H), 3.78 (s, 3H), 3.44 (s, 3H), 2.96 (dd, *J* = 14.7, 8.1 Hz, 1H), 2.18 (m, 1H), 1.74-1.54 (m, 3H), 1.37 (s, 9H), 1.02-0.77 (m, 12H). $^{13}C$ NMR (75 MHz, $CDCl_3$) δ 174.6, 172.0, 169.7, 159.5, 155.2, 131.2, 128.9, 114.2, 98.3, 81.2, 72.2,

58,0, 57.4, 56.1, 55.6, 51.8, 42.3, 41.2, 31.4, 30.0, 28.6 (x3), 25.2, 23.2, 22.6, 19.3, 18.0. $[\alpha]_D^{20}$ = -15.75 (c 0.36 in $CHCl_3$).

*N-Boc-(-)-3-Ar-β-Morph-L-Leu-L-Val-(-)-3-Ar-β-Morph-L-Leu-L-Val-OBn (-)-(**4**). Method A.* According to *procedure A* reported for peptide **3**, tripeptide **12** (1 equiv.) was made to react with **11** (1.1 equiv.). Purification of the crude product by silica gel flash chromatography (AcOEt/*n*hexane, 1:1) afforded peptide **4** (8 %) as a colorless oil. *Method B.* According to *procedure B* reported for peptide **3**, tripeptide **12** (1 equiv.) was made to react with **11** (1.1 equiv.). The purification of the crude product by silica gel flash chromatography (*n*hexane/ AcOEt 1:1) afforded **4** (36%) as colorless oil. *Method C.* Operating in a round-bottom flask equipped with a magnetic stirrer and thermometer, acid **12** (20 mg, 0.034 mmol, 1 equiv.) was dissolved in $CH_2Cl_2$ (2 mL). The solution was cooled to 0 °C. HOBt (5.13 mg, 0.037 mmol, 1.1 equiv.) and EtCN-oxime (5.39 mg, 0.037 mmol, 1.1 equiv.) were added. After 1 h, tripeptide **11** (23 mg, 0.037 mmol, 1.1 equiv.) and DIPEA (13.2 μL, 0,74 mmol, 2.1 equiv.) were added. The reaction mixture was stirred for 24 h at 25 °C. The organic layer was washed with a solution of $KHSO_4$ (5%, 5 mL), a saturated solution of $NaHCO_3$ (5 mL) and brine (5 mL). After purification by silica gel flash chromatography (AcOEt/*n*hexane: 1:1) peptide **4** (8%) was isolated as colorless oil. Detailed NMR data are reported in Table TS5 and IR in Figure S8. HRMS (ESI-TOF): m/z $[M+Na]^+$ Calcd for $C_{60}H_{86}N_6O_{15}Na$ 1153.6049; Found 1153.6052. $[\alpha]_D^{20}$ = -41.3 (*c* 0.36 in $CHCl_3$).

**Table TS3**. $^1H$, $^{13}C$ NMR ($CDCl_3$, 750μL, 0.036 mM, 300 MHz,) and NOEs (600 ms) data for (-)-Boc-3-Ar-β-Morph **2**

| AA | Atom | $^1H$ δ | Molteplicity *J* (Hz) | $^{13}C$ δ | Noesy |
|---|---|---|---|---|---|
| | COOH | | | 173.9 | |
| | CH-2 | 5.42 | d *J* 5.1 | 55.3 | Boc(w), Ar(7.38,m), H-3 (m) |
| | CH-3 | 4.79 | d *J* 5.1 | 72.7 | H-2 (m), $H_{ax}$-5 (w), $H_o$(m), ArO*Me*(vvw) |
| | $CH_2$-5 | $H_{eq}$ 4.19<br>$H_{ax}$ 2.90 | brd *J* 14.0, 5.4<br>dd *J* 14.0, 8.9 | 41.9 | $H_{ax}$-5(s), H-6(m)<br>---------<br>$H_{eq}$-5(vs), H-3(w), H-6(vw), Ar(7.38,m) |
| **ArMorph** | CH-6 | 5.05 | dd , *J* 8.5, 5.4 | 97.5 | OMe-6 (s), H-5($s_{eq}$; $vw_{ax}$) |
| | OMe | 3.56 | | 56.3 | H-2(w), H-6 (m) |
| | MeOAr | MeO: 3.82<br>$H_m$: 6.89<br>$H_o$: 7.38 | s<br>dd, *J* 8.8 | 55.2<br>113.9<br>128.7<br>$C_q$130.1, 159.2 | MeO: $H_m$ (s)<br>$H_m$: ArO*Me*(s)<br>$H_o$: Boc (w), H-3(m), H-2(m), $H_{ax}$-5(m) |
| | Boc | 1.37 | | 28.3, 81.0, 154.7 | H-2 (w), $H_o$ (w) |

**B)**

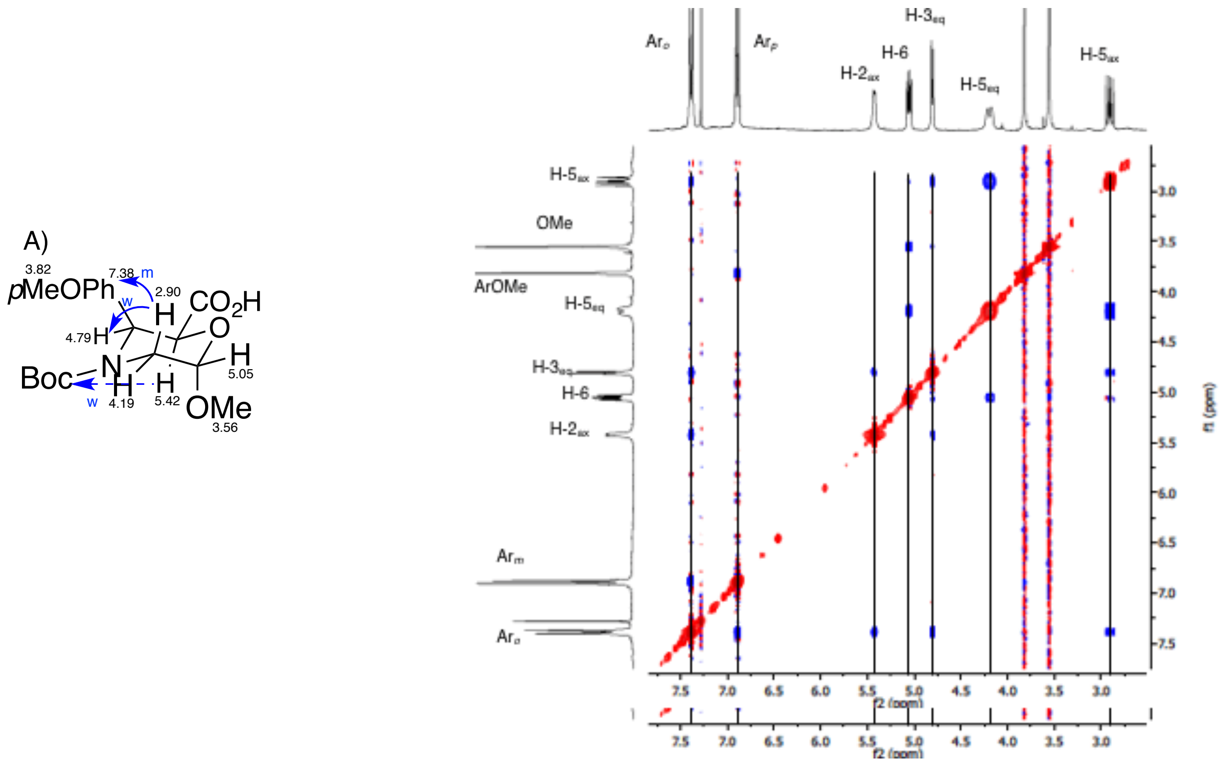


**Figure S4.** NOEs ($CDCl_3$, 300 MHz, 600 ms) of Boc-3-Ar-morpholino acid (-)-**2**. A) More significant NOEs are indicated. B) Ar/CH and CH/CH NOEs region.

**Table TS4.** $^1$H, $^{13}$C NMR ($CD_3CN$, 750µL 0.020 mM, 500 MHz,) and NOEs (600 ms) data for *N*-Boc-(-)-Ar-β-Morph-*L*-Leu-*L*-Val-OBn (-)-**3**.

| AA | Atom | $^1$H δ | Molteplicity *J* (Hz) | $^{13}$C δ | Noesy |
|---|---|---|---|---|---|
| **ArMorf-1** | CO | | | 168.5[a] | |
| | CH-2 | 5.13 | d *J* 8.0 | 56.8 | Boc(vw), $NH_{Leu}$ (w), $H_o$(m), H-3(w) |
| | CH-3 | 4.45 | d *J* 8.0 | 72.1 | H-2 (w), $H_{ax}$-5(m), ArO*Me* (w), $H_o$(m) |
| | $CH_2$-5 | $H_{eq}$ 4.27<br><br>$H_{ax}$2.96 | dd *J* 14.6, 5.5<br><br>d *J* 14.6, 8.2 | 41.7 | H-6(m), $H_{ax}$-5(s)<br>---------<br>$H_{eq}$-5(s), H-3(m), H-6(w), $H_o$(m) |
| | CH-6 | 4.94 | dd, *J* 8.2, 5.5 | 97.8 | OMe (m), $NH_{Leu}$ (w) H-5($m_{eq}$, $w_{ax}$) |
| | OMe | 3.45 | | 55.0 | $NH_{Leu}$ (w), H-6(m) |
| | MeOAr | MeO: 3.79<br>$H_m$: 6.89<br>$H_o$: 7.27 | s<br>AA'BB' system *J* 8.7 | 54.7<br>113.5<br>128.5<br>$C_q$131.3, 159.1 | $H_m$(s)<br>$H_m$: ArO*Me* (s)<br>$H_o$: Boc(m), H-3(m), $H_{ax}$-5(m), H-2(m) |
| | Boc | 1.37 | | 27.6, 80.0<br>154.6 | H-2 (vw), $H_o$(m) |
| **Leu-2** | CO | | | 171.2[a] | |
| | CH | 4.47-4.43 | m | 51.3 | $NH_{Val}$ (s), *$Me_2$CH$CH_2$* (m) |
| | CH<br><br>$CH_2$ | 1.71-1.64<br><br>1.60-1.59 | m<br><br>m | 24.5<br><br>40.8 | $Me_{Leu}$(s), *NH*(w)*$CH_{Leu}$*vw),<br><br>*$NHCH_{Leu}$*(m), $Me_{Leu}$(m) $NH_{Val}$(w) |
| | Me | 0.93<br>0.95 | d *J* 6.0<br>d *J* 6.4 | 21.0 | N*CH*(s), *$CHCH_2$* (s) |

| | | | | | |
|---|---|---|---|---|---|
| | NH | 7.12 | d *J* 8.6 | | CH(s)$CH_2$(w), $NH_{Val}$ (vw), H-6(w), H-2(w), OMe(w) |
| **Val-3** | CO | | | 171.8[a] | |
| | CH | 4.32 | m | 57.6 | *$Me_2CH$*(s), $NH_{Val}$(m) |
| | CH | 2.16-2.10 | m | 30.5 | $CH_{Val}$(m), $NH_{Val}$(vw) |
| | Me | 0.90<br>0.88 | d *J* 6.8<br>d *J* 6.7 | 18.3<br>17.5 | $NH_{Val}$(m)<br>$Me_2$*CHCH* (s) |
| | NH | 6.84 | d, *J* 7.8 | | $CH_{2Leu2}$(vw), $CH_{Leu}$(s), $NH_{Leu}$(vw), $Me_2$*CH*$_{Val}$(w), $CH_{Val}$(m), $Me_{Val}$ (m) |
| | OBn | $OCH_2$ 5.17, 5.12<br>Ph 7.27, 7.89 | AB system *J* 12.2 | 66.5<br>136.0, 128.5, 128.3, 128.2 | Ph(w)<br>7.27: $OCH_2$ (s), $Me_{Val}$ (m) |

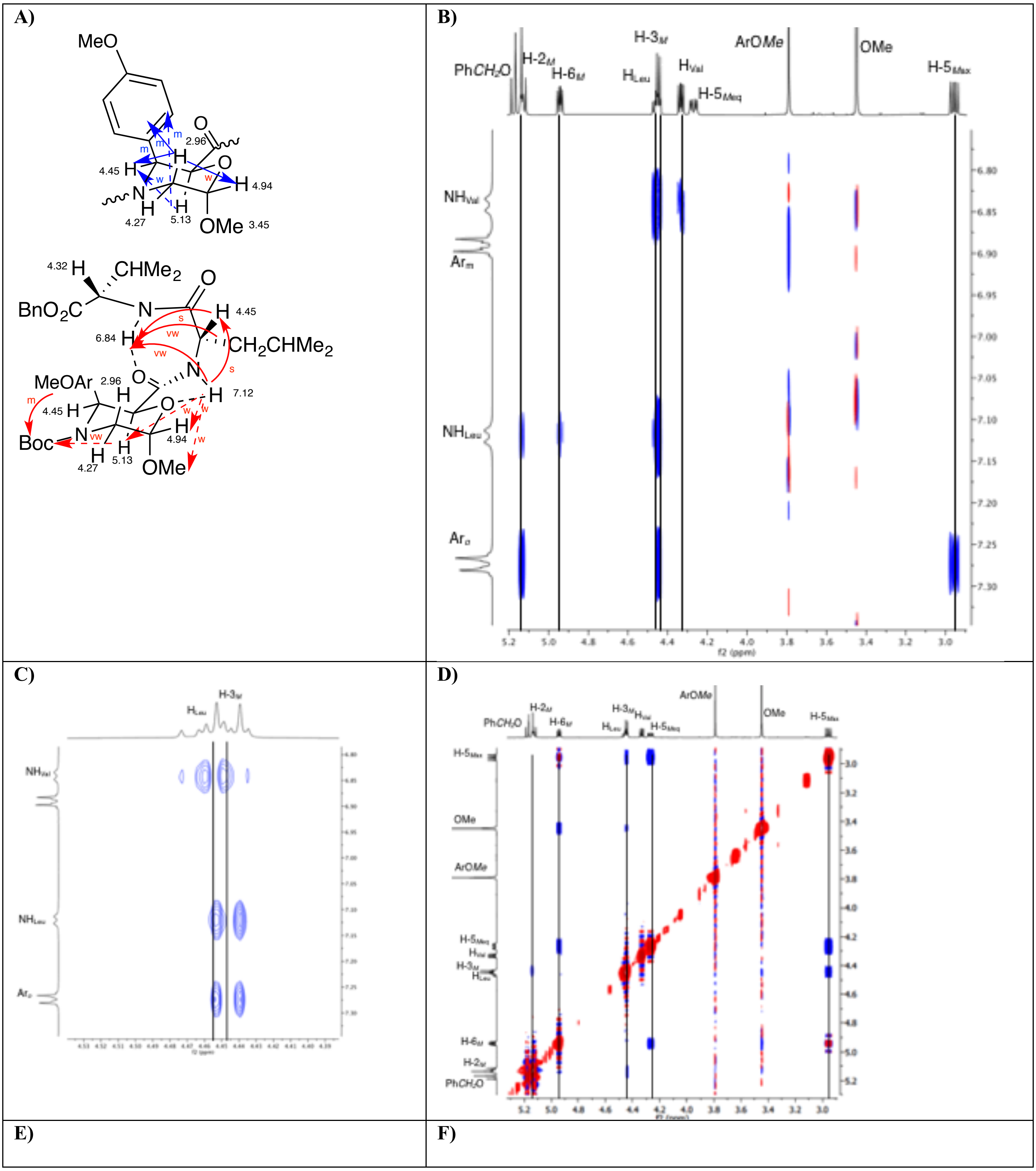
A)
B)
C)
D)
E)
F)
MeO
OMe
BnO2C
CHMe2
CH2CHMe2
MeOAr
Boc
PhCH2O
H-2M
H-6M
H-3M
HLeu
HVal
H-5Meq
ArOMe
OMe
H-5Max
NHVal
Arm
NHLeu
Aro
f2 (ppm)

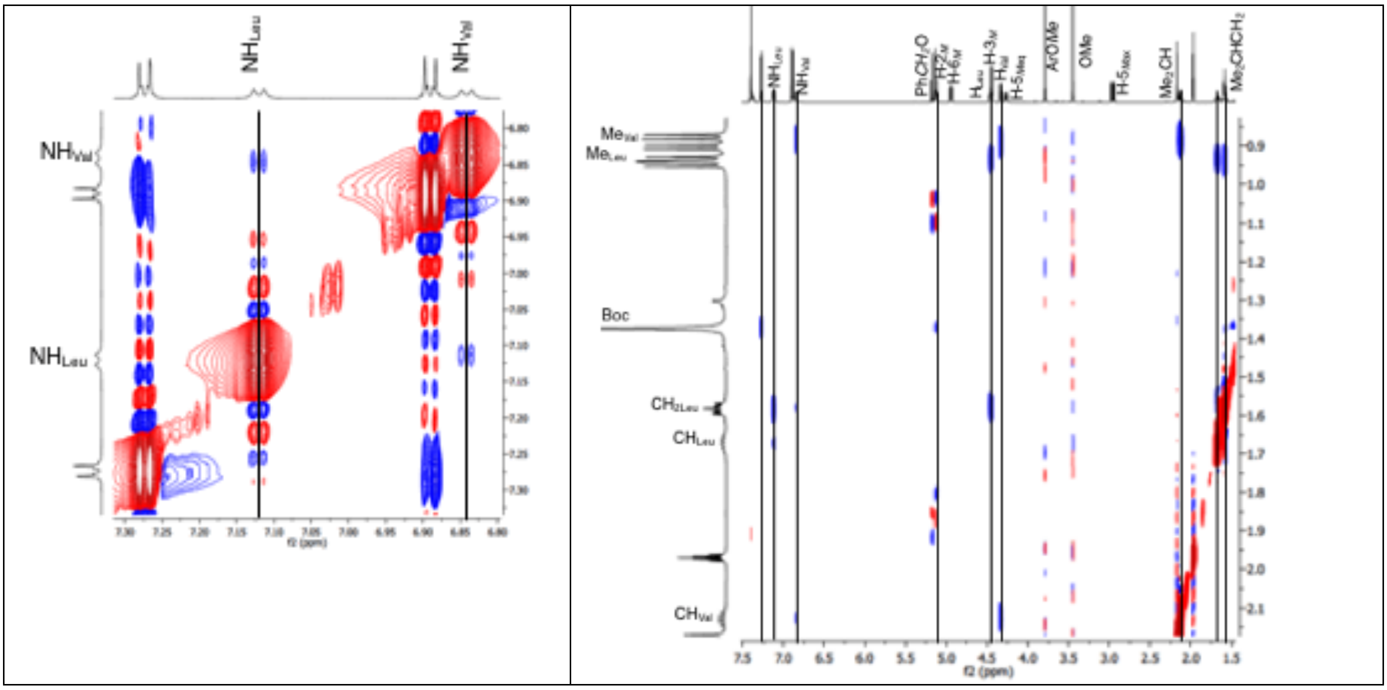


**Figure S5.** NOESY for tripeptide (-)-**3** ($CD_3CN$, 500 MHz, 600ms): A) NOEs of morpholino ring protons (blue arrows) and between the different amino acids (red arrows). H-bonds (dotted lines). B) CH/Ar and CH/NH region. c) Zoom of CH-NH region. D) CH/CH region. E) zoom NH/NH region. F) Hight field/all protons region.

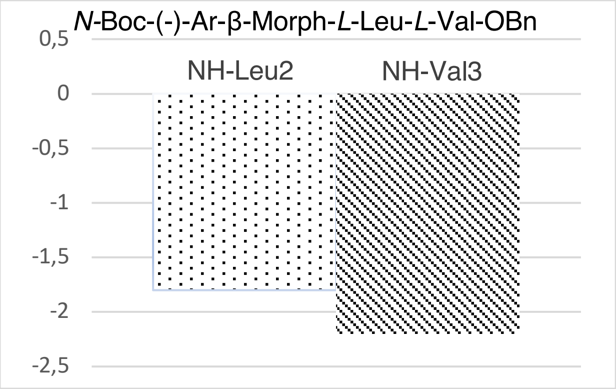


**Figure S6.** Δδ/ΔT NH values for peptide (-)-**3** (273-323 K; $CD_3CN$, 600 MHz).

**Table S5.** $^{1}$H, $^{13}$C NMR ($CD_3CN$, 750μL 0.021 mM, 500 MHz,) and NOEs (600 ms) data for *N*-Boc-(-)-Ar-β-Morph-*L*-Leu-*L*-Val-(-)-Ar-β-Morph-*L*-Leu-*L*-Val-OBn (-)-**4**

| AA | Atom | $^{1}$H δ | Molteplicity *J* (Hz) | $^{13}$C δ | Noesy |
|---|---|---|---|---|---|
| **ArMorf-1** | CO | | | 168.7 | |
| | CH-2 | 5.14 | d *J* 7.6 | 56.9 | Boc(w), $NH_{Leu2}$ (w), Ar(7.28,s), H-$3_{M1}$(w) |
| | CH-3 | 4.45 | d *J* 7.6 | 72.3 | H-$2_{M1}$ (w), $H_{ax}$-$5_{M1}$ (m), $H_o$ (s), OMe(w) |
| | $CH_2$-5 | $H_{eq}$ 4.27<br>$H_{ax}$2.95 | Overl.<br>dd *J* 14.4, 8.0 | 41.8 | ------<br>$H_{eq}$-$5_{M1}$(vs), H-$3_{M1}$(m), H-$6_{M1}$ (w), Ar(7.28,m) |
| | CH-6 | 4.95 | dd, *J* 8.0, 5.7 | 97.8 | OMe (vs), $NH_{Leu2}$ (w), H- $5_{M1}$ (4.27,s; 2.96,w) |
| | 6-OMe | 3.45 | s | [a] | H-$6_{M1}$ (s), H-$3_{M1}$(w) |
| | MeOAr | [b] | [b] | | OMe: $H_m$(s), H-$3_{M1}$(s)<br>$H_m$: OMe (s)<br>$H_o$: Boc (w), H-$3_{M1}$(m), $H_{ax}$-$5_{M1}$(m), H$2_{M1}$(s), $H_m$(s) |

| | | | | | |
|---|---|---|---|---|---|
| | Boc | 1.37 | | 27.5, 80.1<br>154.7 | H-2$_{M1}$ (w), H$_{eq}$-5$_{M1}$(vw), Ar$_o$(w) |
| **Leu-2** | CO | | | 171.5 | |
| | CH | 4.44 | Overl. | 51.5 | |
| | CH<br>$CH_2$ | 1.69<br>1.63 | m<br>m | 24.6<br>40.7 | CH$_{Leu}$(m), Me$_{Leu}$(s)<br>Me$_{Leu}$(s) |
| | Me | 0.94<br>0.98 | d *J* 6.2<br>d *J* 6.7 | 22.5 | *CHCH*$_2$(s)*CH*N (m)<br>CH$_{2Leu}$(s) |
| | NH | 7.17 | d *J* 8.5 | | *CH*(w)*CH*$_2$(m)*CH*N$_{Leu}$ (s), NH$_{Val3}$ (vw)<br>OMe(vvw), H-6$_{M1}$(vw), H-2$_{M1}$(vvw) |
| **Val-3** | CO | | | 170.7 | |
| | CH | 4.70 | dd *J* 8.5, 6.8 | 53.6 | H$_{eq}$-5$_{M4}$(s), *Me*$_2$*CH*(m), NH$_{Val3}$(vw) |
| | CH | 1.95 | m | 30.8 | Me$_{Val}$ (m), H-5$_{eqM4}$(vw), *NHCH*$_{Val}$ (s) |
| | Me | 0.76<br>0.68 | d *J* 7.0<br>d *J* 6.5 | 18.7<br>16.6 | Ar$_o$(w), Me$_2$*CHCH*(m),<br>Me$_2$*CHCHNH*$_{Val}$(m) |
| | NH | 6.94 | d, *J* 8.5 | | *CH*$_2$(vw)*CH*(m)*NH*(vw)$_{Leu2}$, H$_{eq}$-5$_{M4}$(vw)<br>*CHCH*N$_{Val2}$(w), Me(0.69,m) |
| **ArMorf-4** | CO | | | 168.3 | |
| | CH-2 | 5.46 | d *J* 8.9 | 55.9 | NH$_{Leu5}$ (vw), H$_o$(s), H-3$_{M4}$(m) |
| | CH-3 | 4.56 | d *J* 8.9 | 71.8 | H-2$_{M4}$ (m), H$_{ax}$-5$_{M4}$(m), OMe (3.45, w)<br>Ar(7.28,s) |
| | $CH_2$-5 | H$_{eq}$ 4.26<br>H$_{ax}$3.29 | Overl.<br>dd *J* 14.6, 8.1 | 44.0 | ---------<br>H$_{eq}$-5$_{M4}$(s), H-3$_{M4}$(m), H$_o$(w), H-6$_{M4}$(w) |
| | CH-6 | 5.00 | dd, *J* 8.1, 6.0 | 97.9 | OMe (vs), H- 5$_{M4}$ (4.27s; 3.29w), NH$_{Leu}$ (vvw) |
| | OMe | 3.45 | s | [a] | H-6$_{M4}$ (s), H-3$_{M4}$(w) |
| | MeOAr | [b] | [b] | [b] | OMe: H$_m$(s)<br>H$_m$: OMe (s)<br>H$_o$: H-3$_{M4}$(s), H$_{ax}$-5$_{M4}$ (w), H-2$_{M4}$(s), H$_m$(s)<br>Me$_{Val3}$(w) |
| **Leu-5** | CO | | | 171.8 | |
| | CH | 4.44 | Overl. | 51.5 | |
| | CH<br>$CH_2$ | 1.66<br>1.57 | m<br>m | 24.6<br>40.9 | CH$_{Leu}$ (m), Me$_{Leu}$(s)<br>Me$_{Leu}$(s) |
| | Me | 0.93<br>0.91 | d *J* 6.5<br>d *J* 6.7 | 21.0 | N*CH*(m), *CHCH*$_2$ (s) |
| | NH | 7.08 | d *J* 8.3 | | *Me*(0.93w)*CHCH*$_2$(w),*CH* (m)$_{Leu4}$, NH$_{Val}$ (vw)<br>OMe(vw), H-6$_{M4}$(m), H-2$_{M4}$(m) |
| **Val-6** | CO | | | 170.7 | |
| | CH | 4.33 | dd *J* 8.3, 5.9 | 57.6 | *Me*$_2$*CH*(s), NH$_{Val}$(w) |
| | CH | 2.14 | m | 30.4 | *Me*(s)*CHNH* (m)$_{Val6}$ |
| | Me | 0.89<br>0.87 | d *J* 6.9<br>d *J* 6.9 | 18.4<br>17.6 | Me$_2$*CH*(s)*CH*(w)*NH*(w)$_{Val6}$ |
| | NH | 6.89 | Overl. | | *Me*$_2$(s)*CH*(w)*CH*N(m) $_{Leu5}$, NH$_{Leu5}$(vw) |
| | OBn | OCH$_2$ 5.19, 5.12<br>Ph 7.42-7.34 | AB system<br>*J* 12.3<br>m | 66.5<br>C$_q$136.2,<br>128.8-128.3 | Ph(m)<br><br>OCH$_2$(m) |

[a]$\delta_{OMe}$: 55.2, 55.1; [b]$\delta_{ArOMe}$: 3.78 (OMe); AA'BB' system, 6.86 (113.6), 7.28 (128.7), *J* 8.7; 3.79, AA'BB' system, 6.90 (113.8), 7.28 (128.7), *J* 8.5; C$_q$(159.9, 131.4, 130.4).

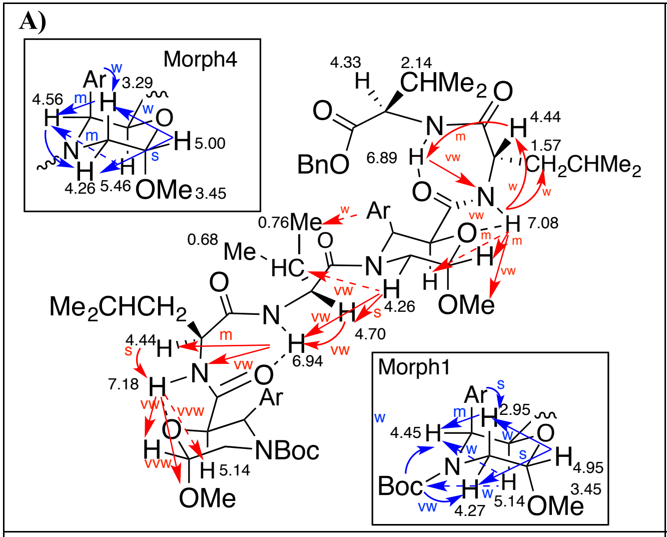


**Figure S7.** NOESY for hexapeptide (-)-**4** ($CD_3CN$,0.021 mM, 500 MHz, 500 ms): A) NOEs of morpholino ring protons (blu arrows) and between the different amino acids (red arrows); H-bonds (dotted lines). B) CH/Ar and CH/NH region. c) Zoom of NH-NH region. D) CH/CH region. F) Hight field/medium field CH region.

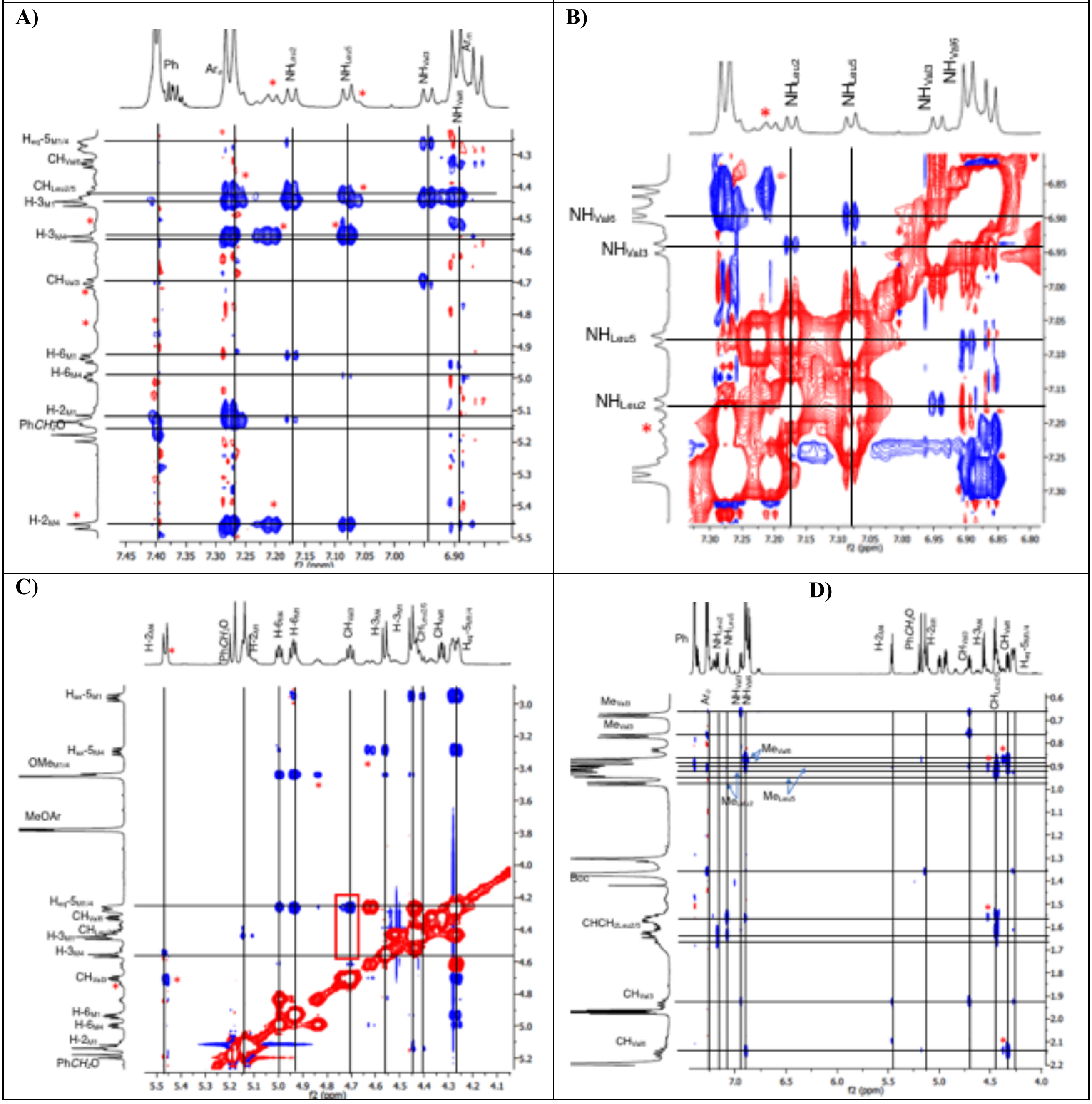

# 3. $^1$H NMR and $^{13}$C NMR

## Compound 7

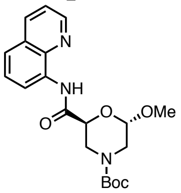


$^1$H NMR ($CDCl_3$, 300 MHz)

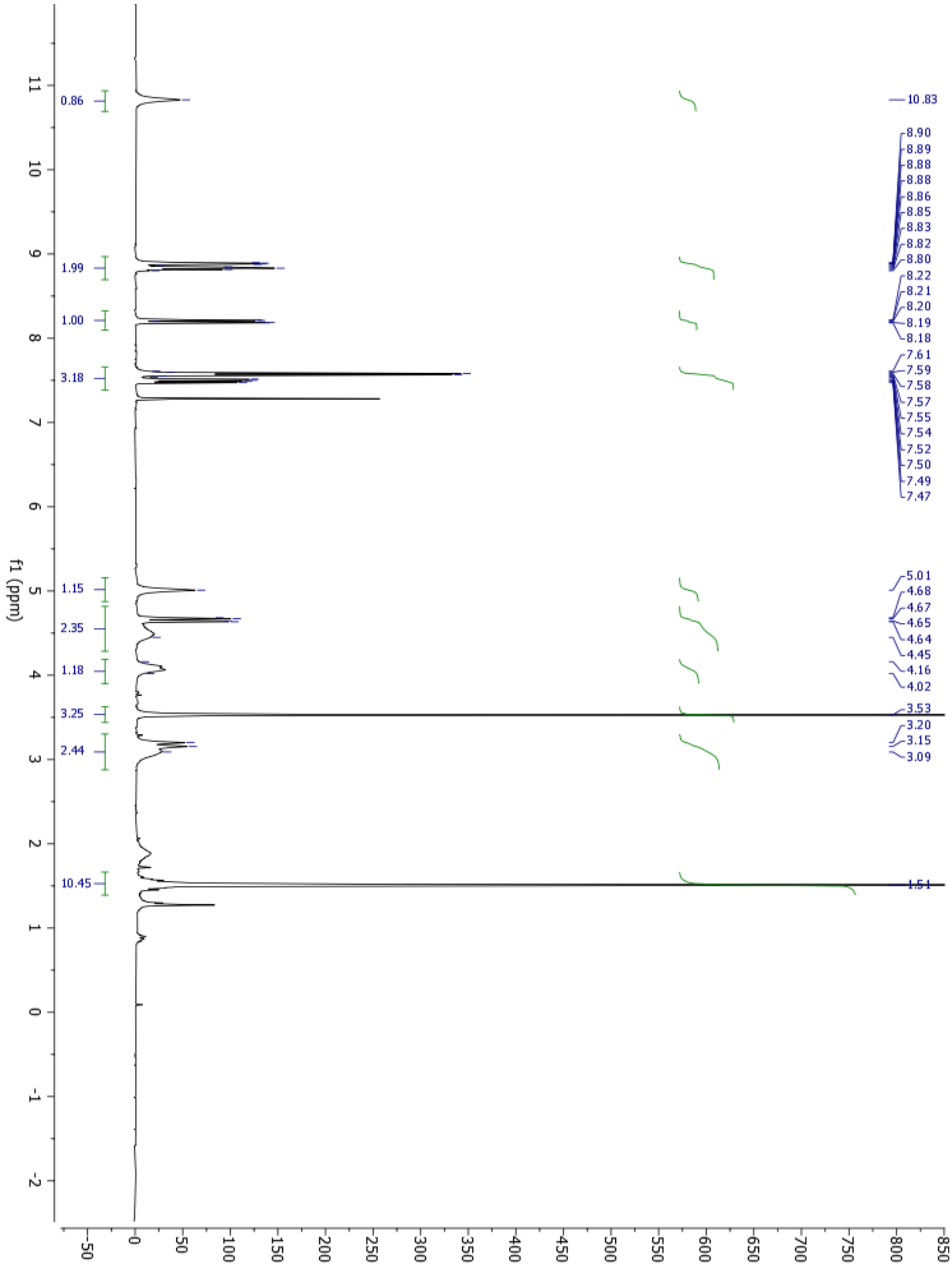

$^{13}C$ NMR (75 MHz, $CDCl_3$)

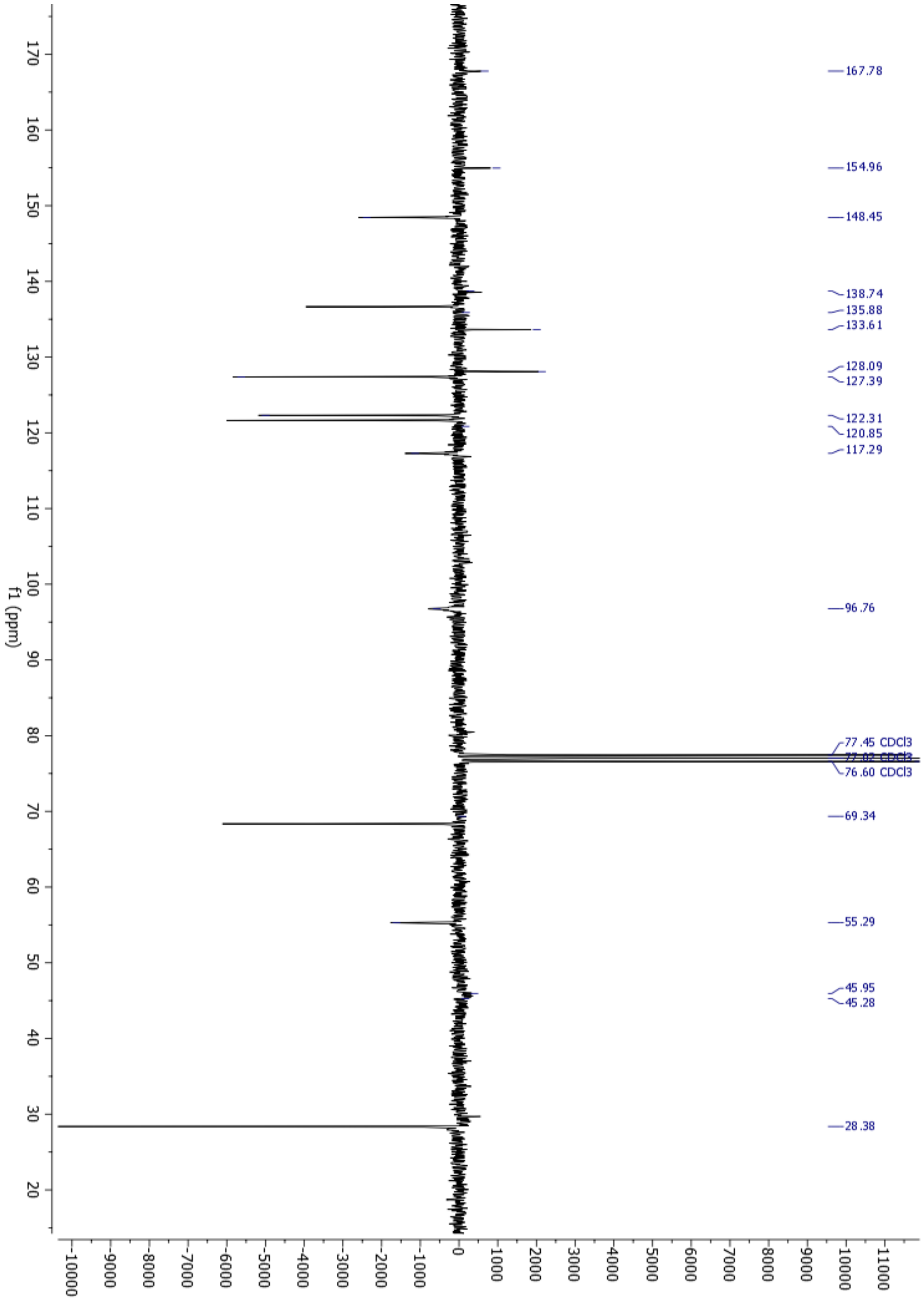

**Compound 8**

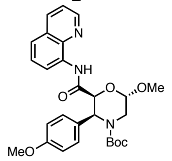


$^1H$ NMR (300 MHz, $CDCl_3$)

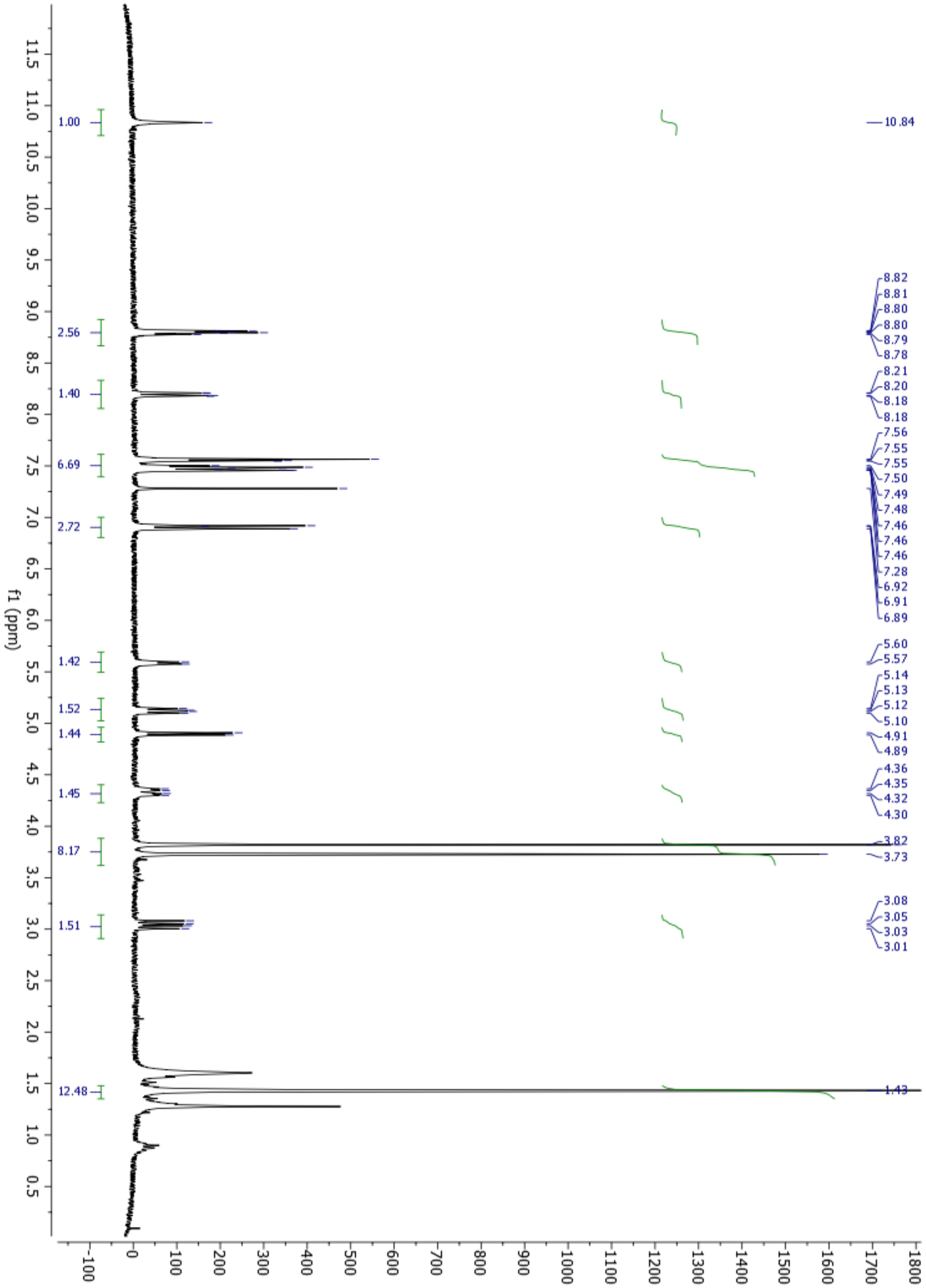

$^{13}C$ NMR (75 MHz, $CDCl_3$)

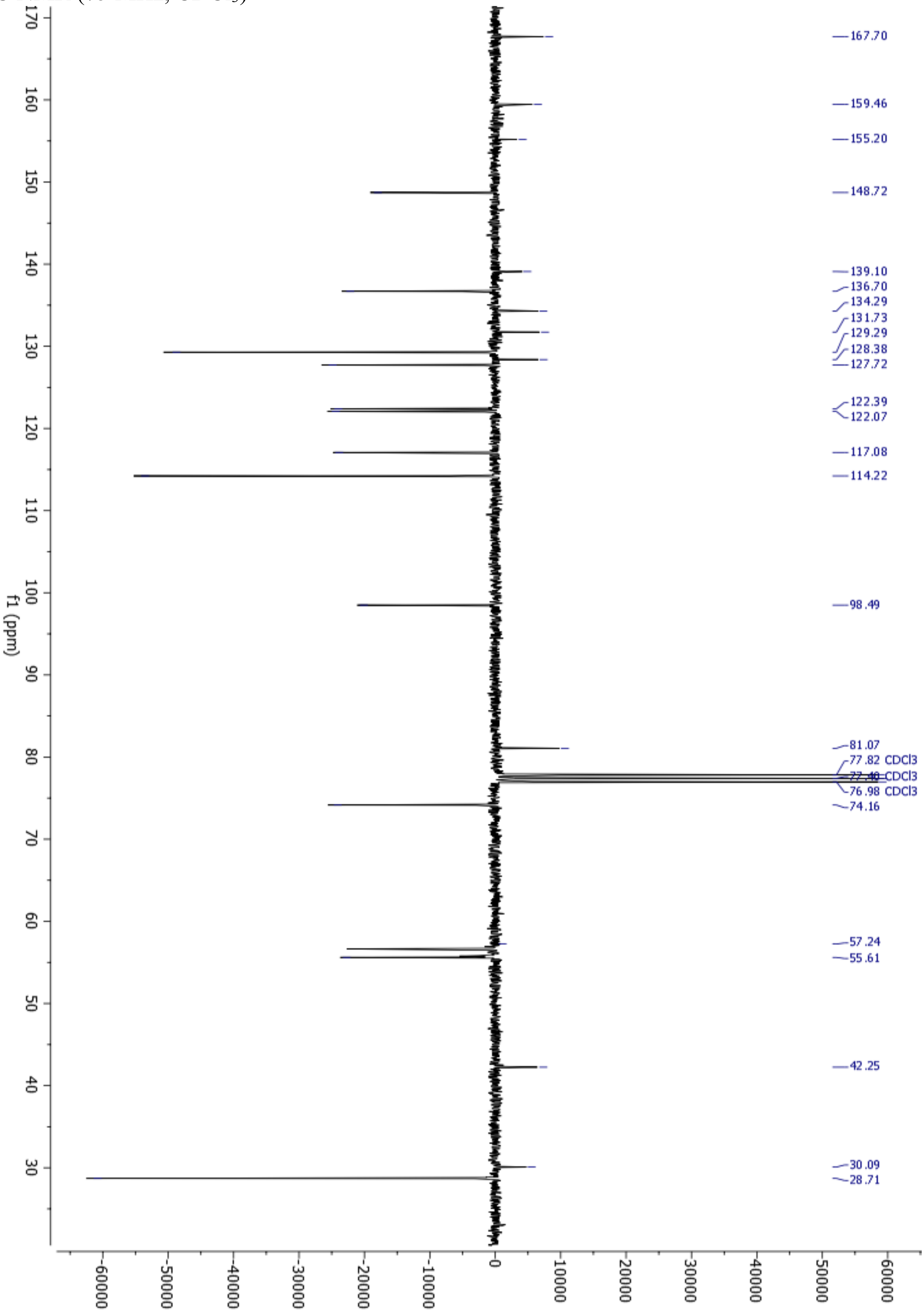

**Compound 9**

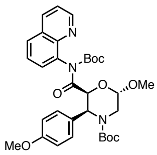


$^{1}$H NMR (300 MHz, $CDCl_3$)

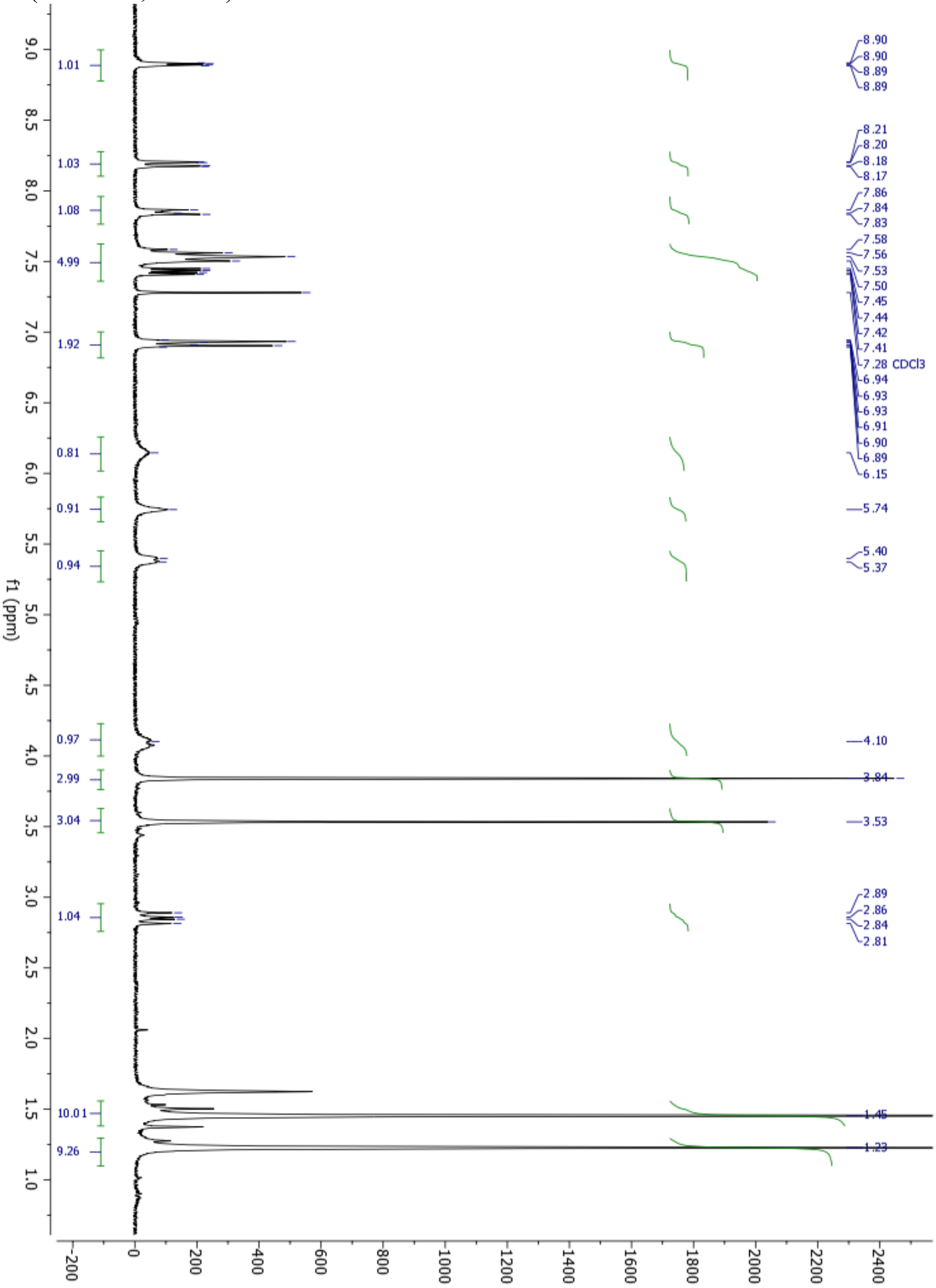

$^{13}C$ NMR (75 MHz, $CDCl_3$)

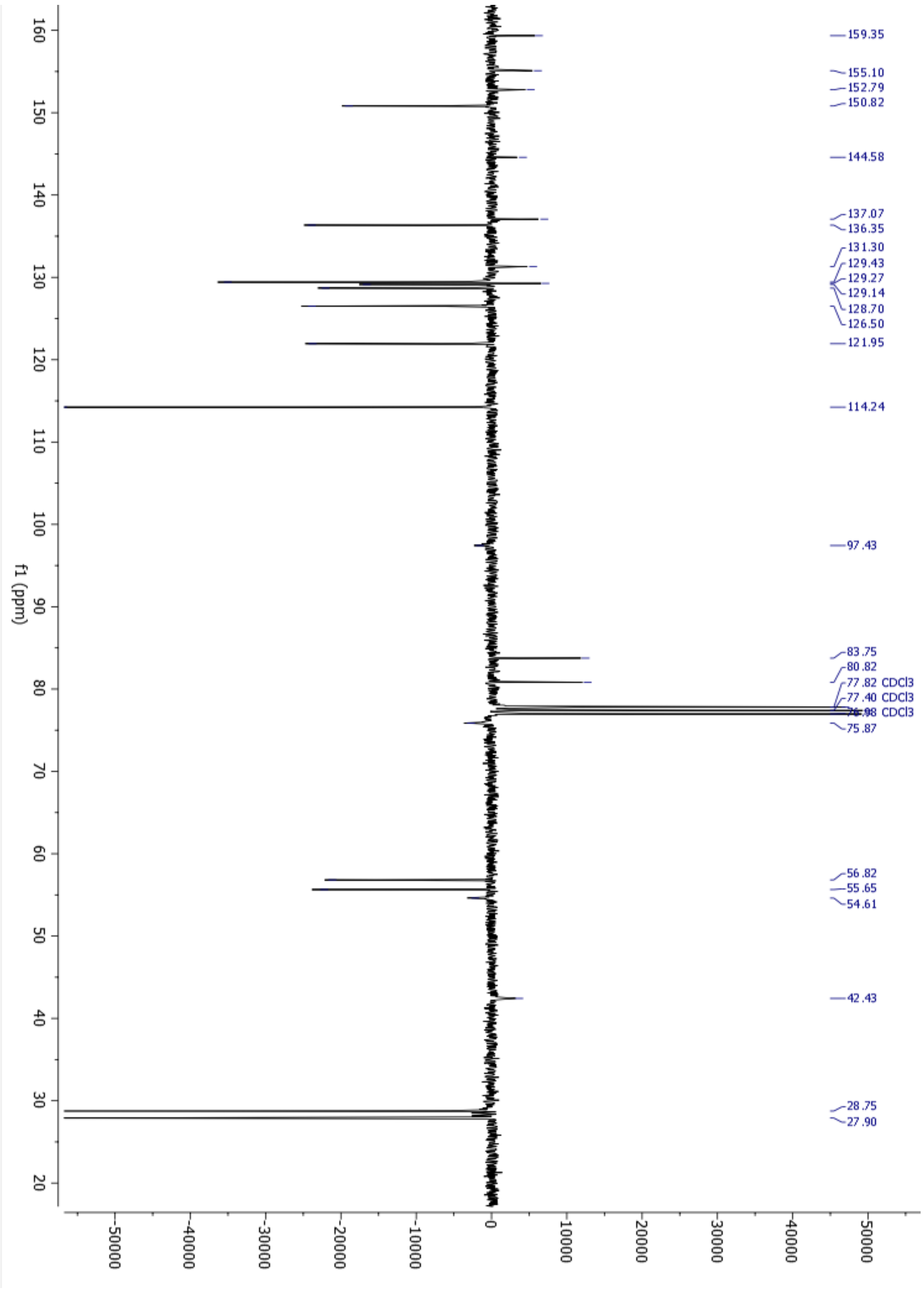

**Compound 2**

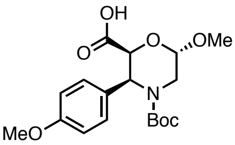


$^1$H NMR (300 MHz, $CDCl_3$)

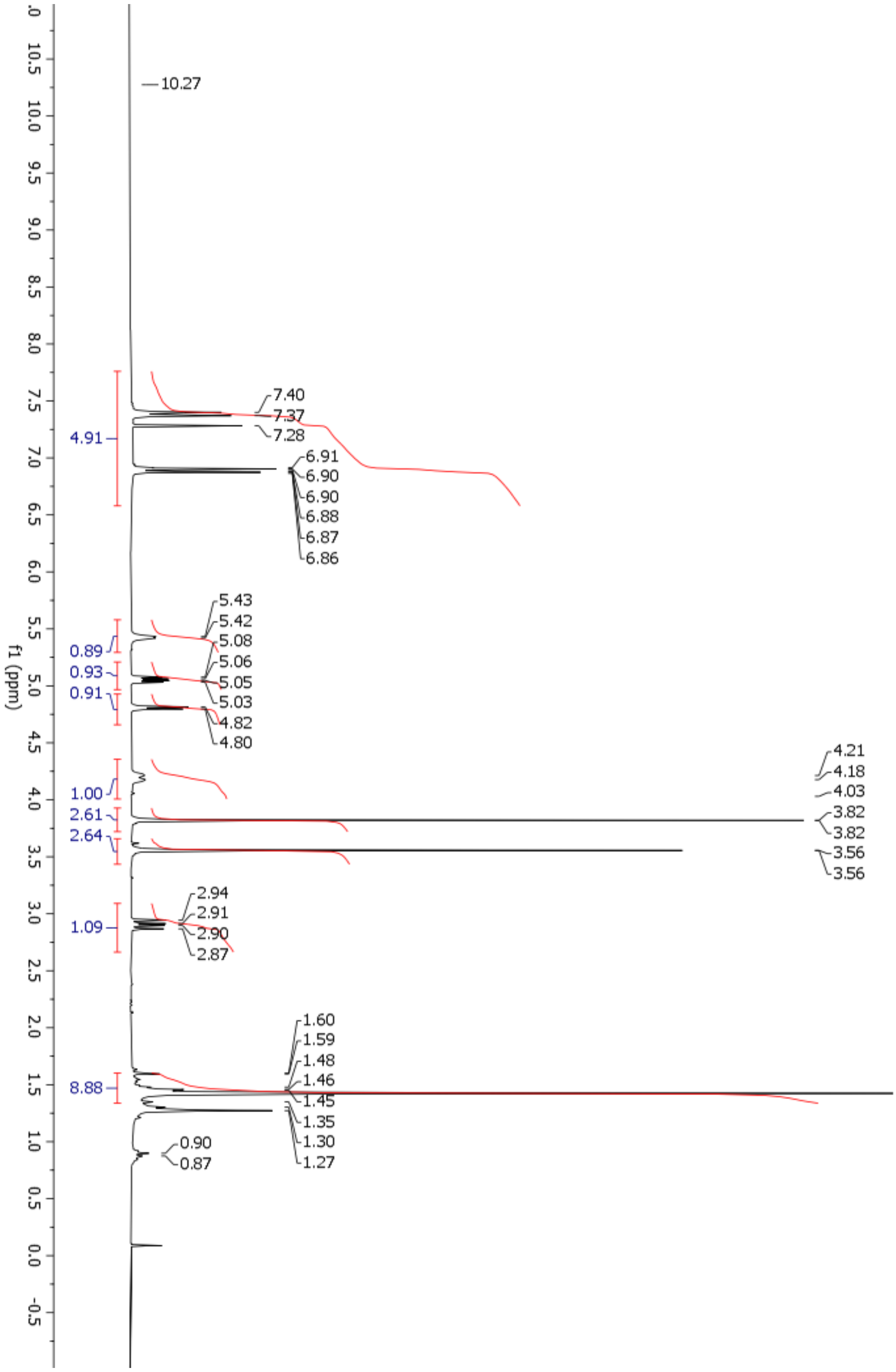

$^{13}C$ NMR (75 MHz, $CDCl_3$)

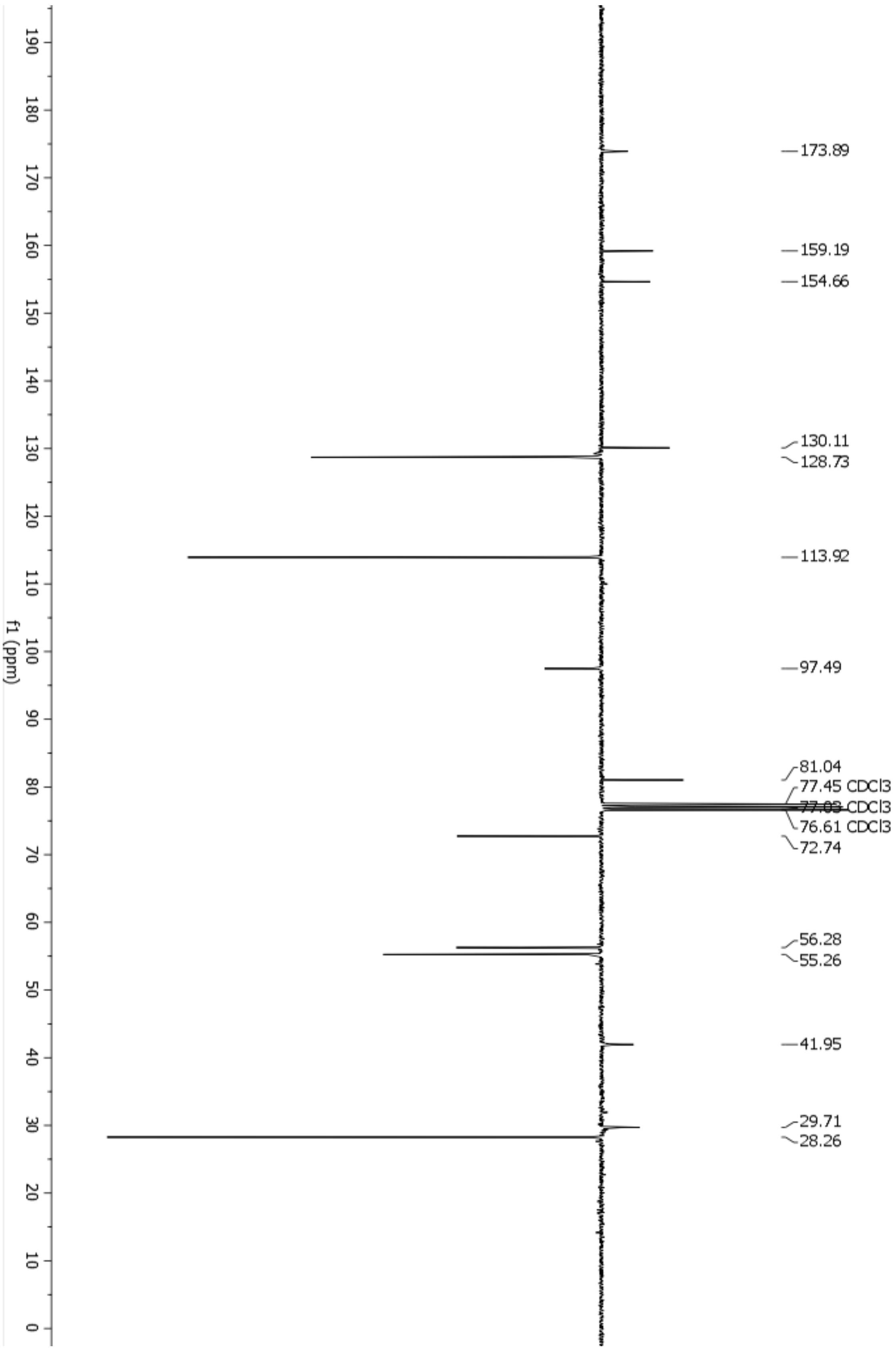

**Compound 3**

$^1$H NMR (500 MHz, $CD_3CN$)

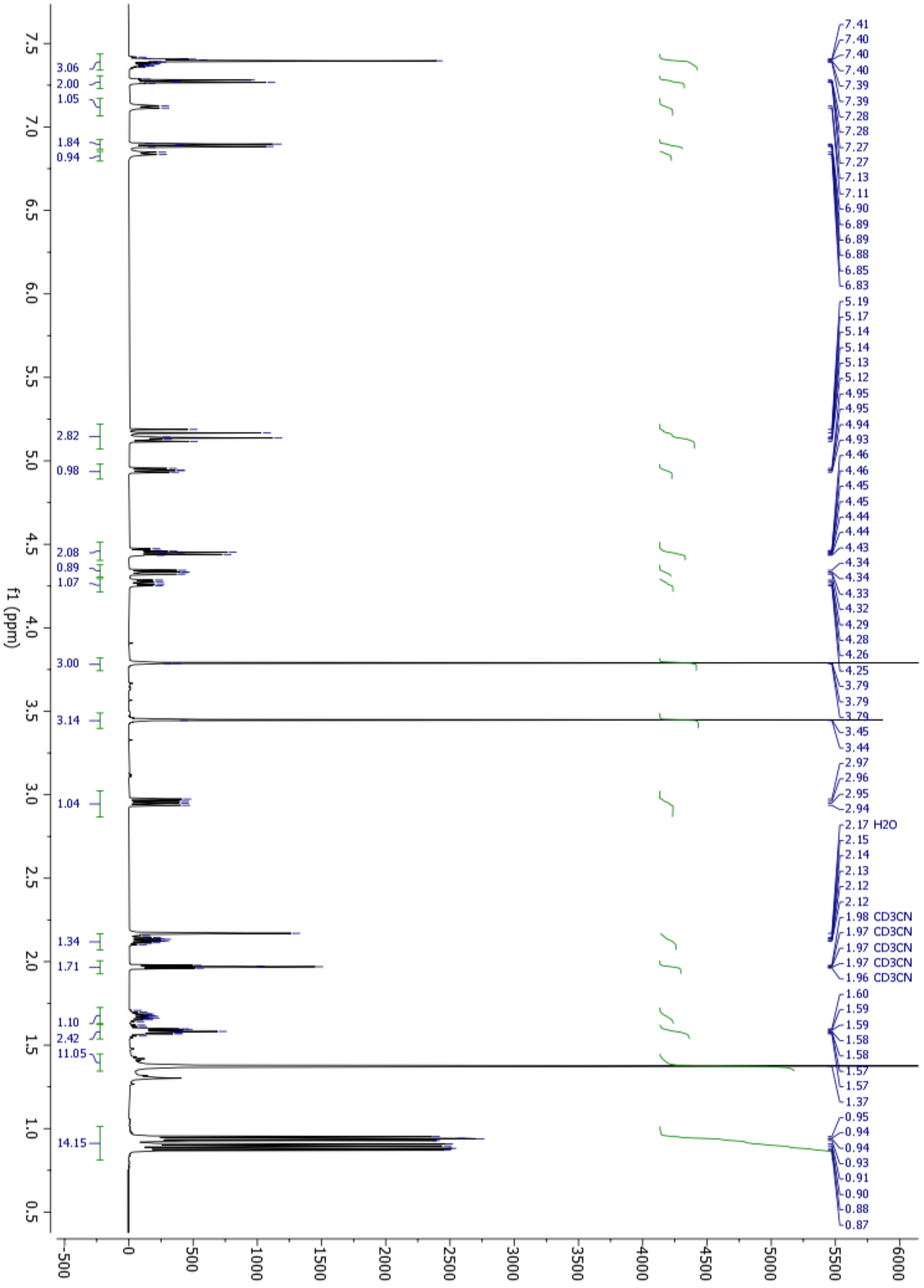

$^{13}C$ NMR (125 MHz, $CD_3CN$)

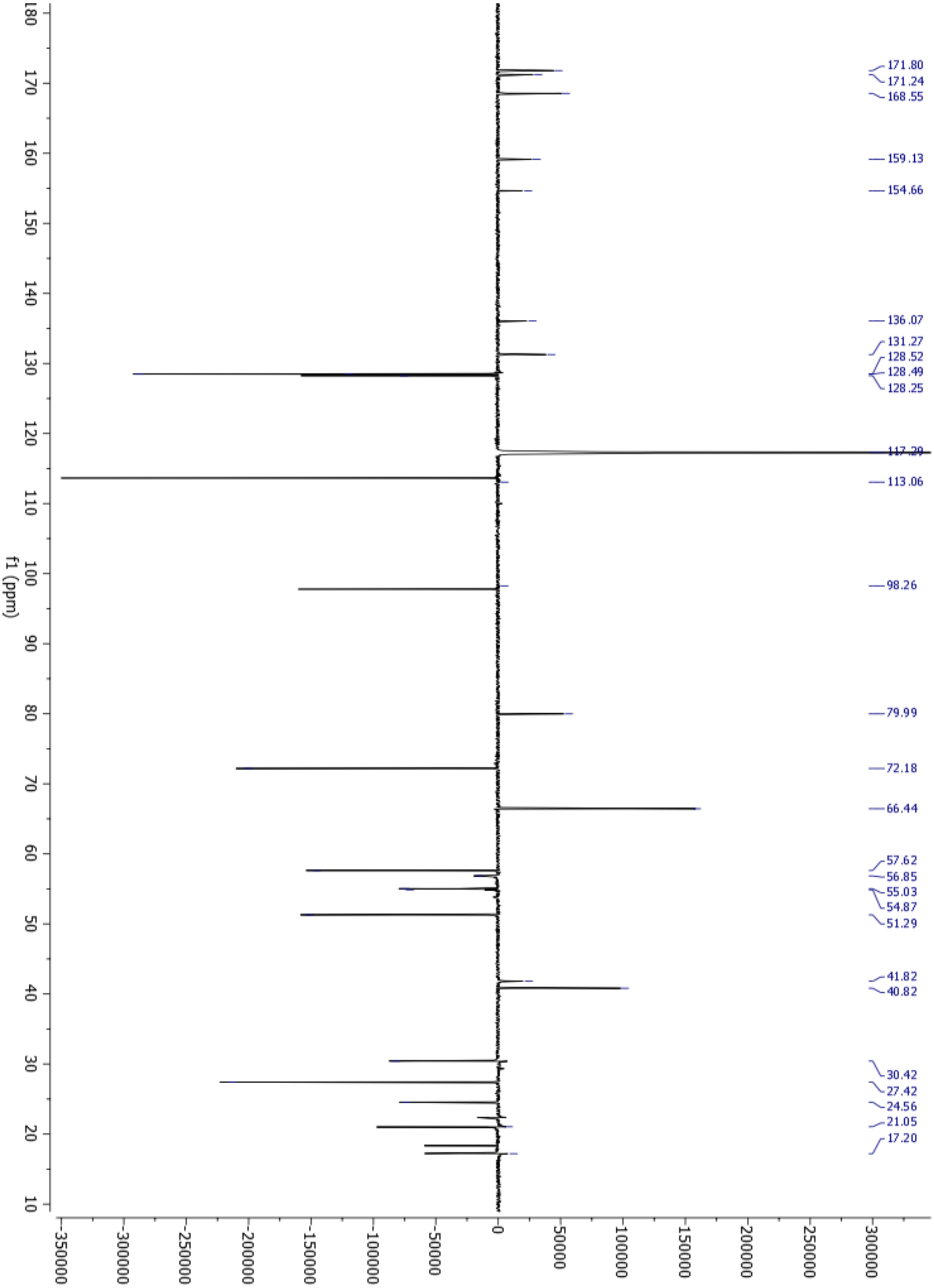

**Compound 11**

$^{1}H$ NMR (300 MHz, $CDCl_3$)

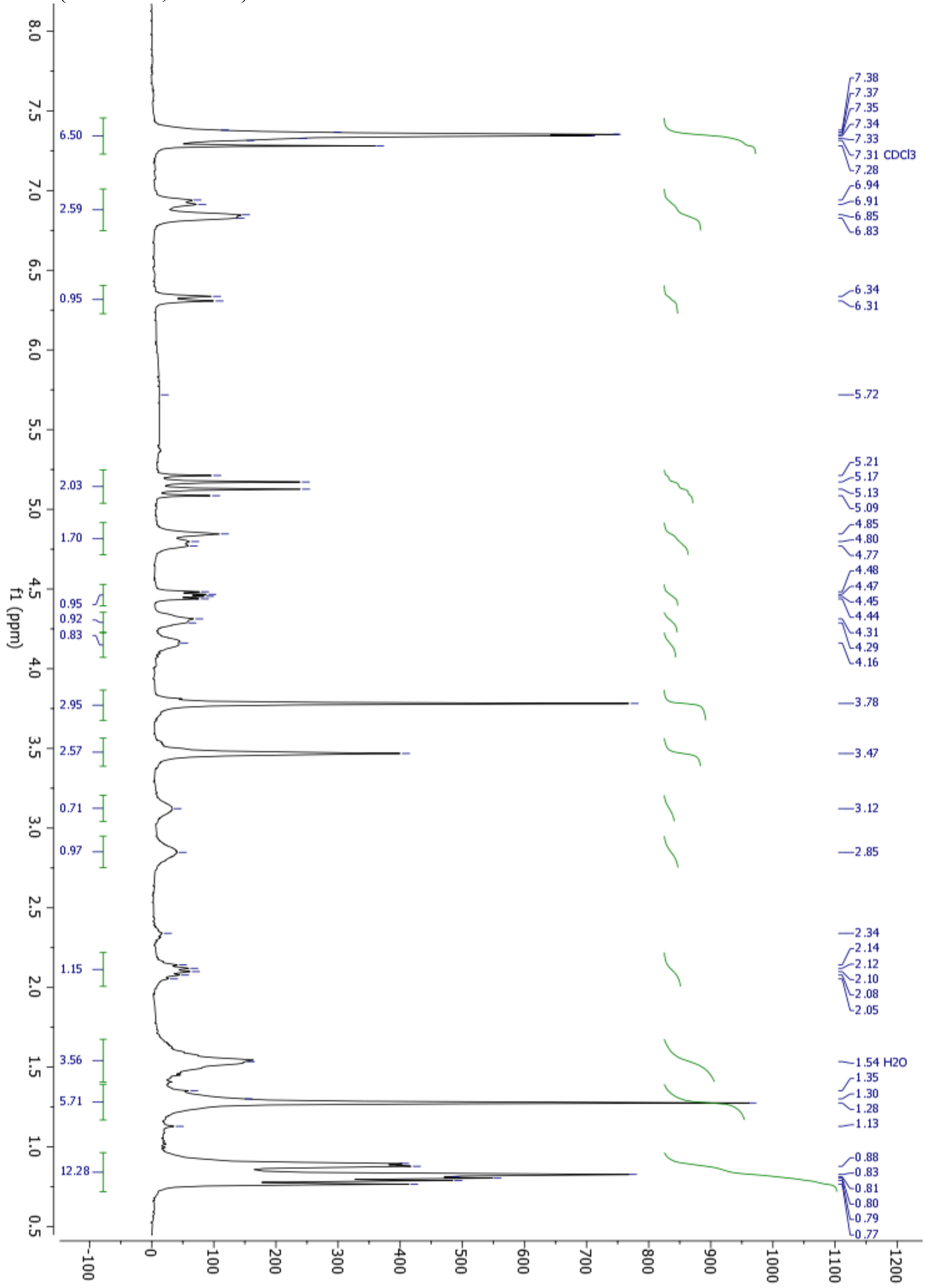

$^{13}C$ NMR (75 MHz, $CDCl_3$)

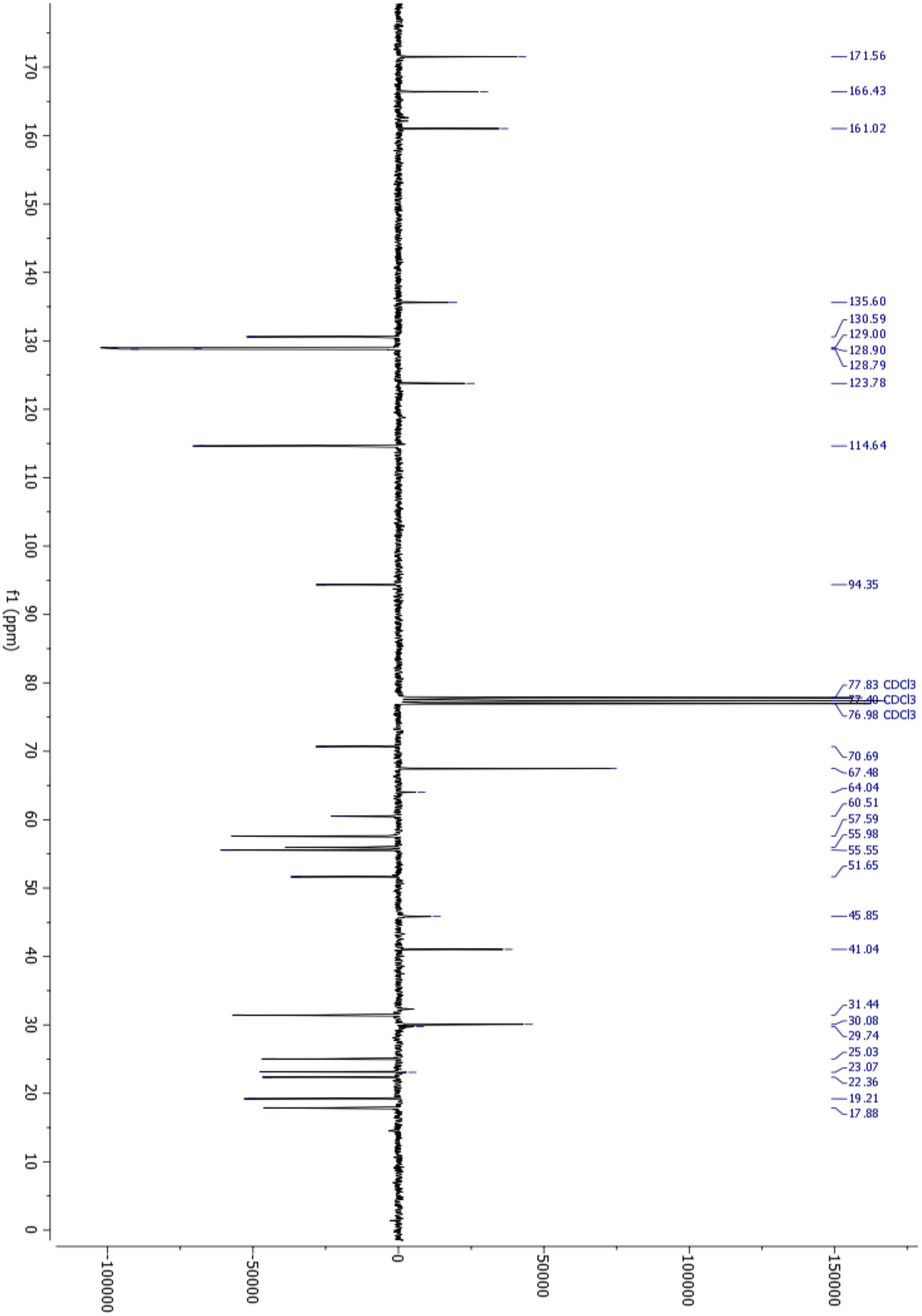

**Compound 12**

$^{1}H$ NMR (300 MHz, $CDCl_3$)

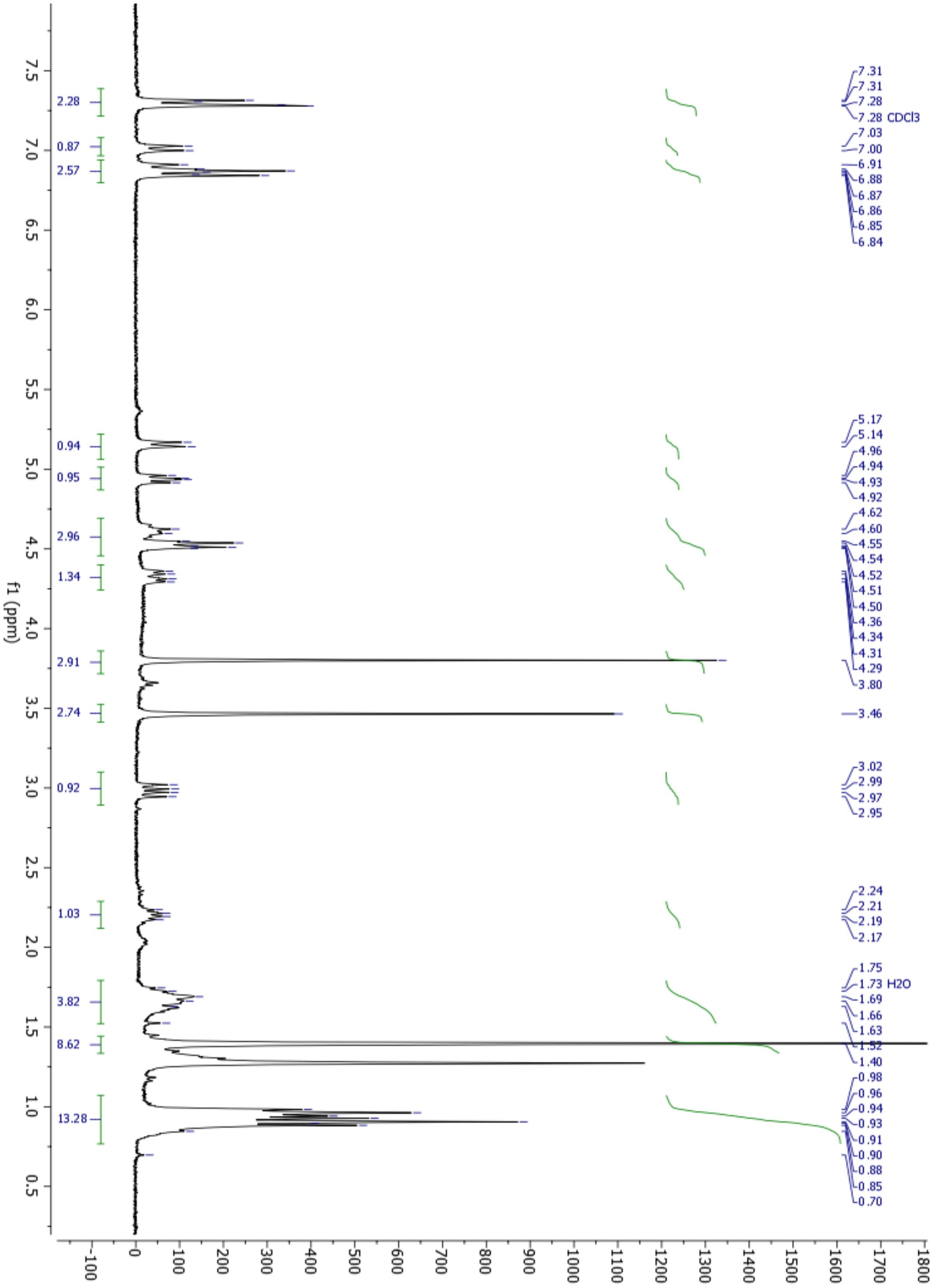

$^{13}$C NMR (75 MHz, $CDCl_3$)

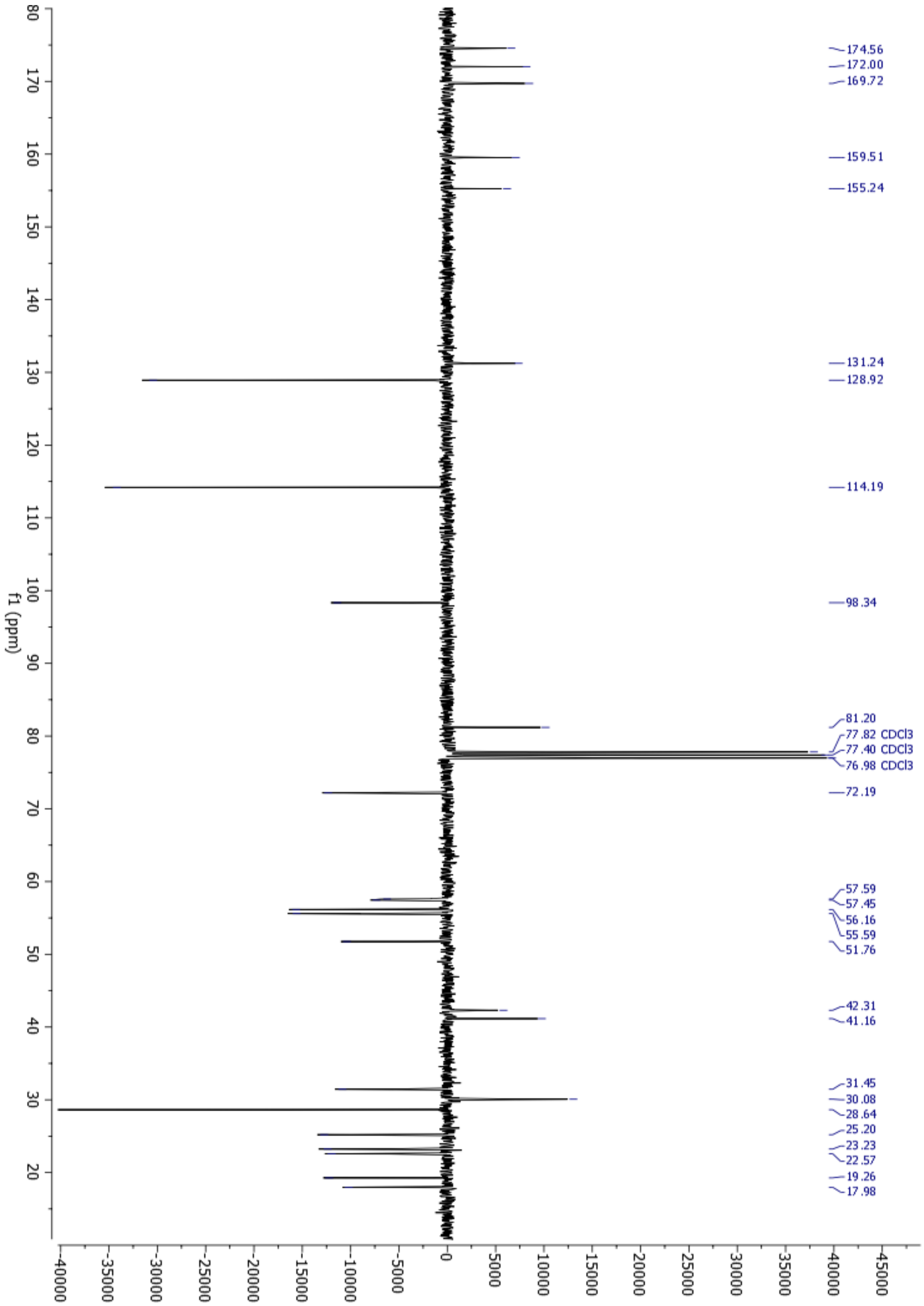

**Compound 4**

$^{1}H$ NMR (500 MHz, $CD_3CN$)

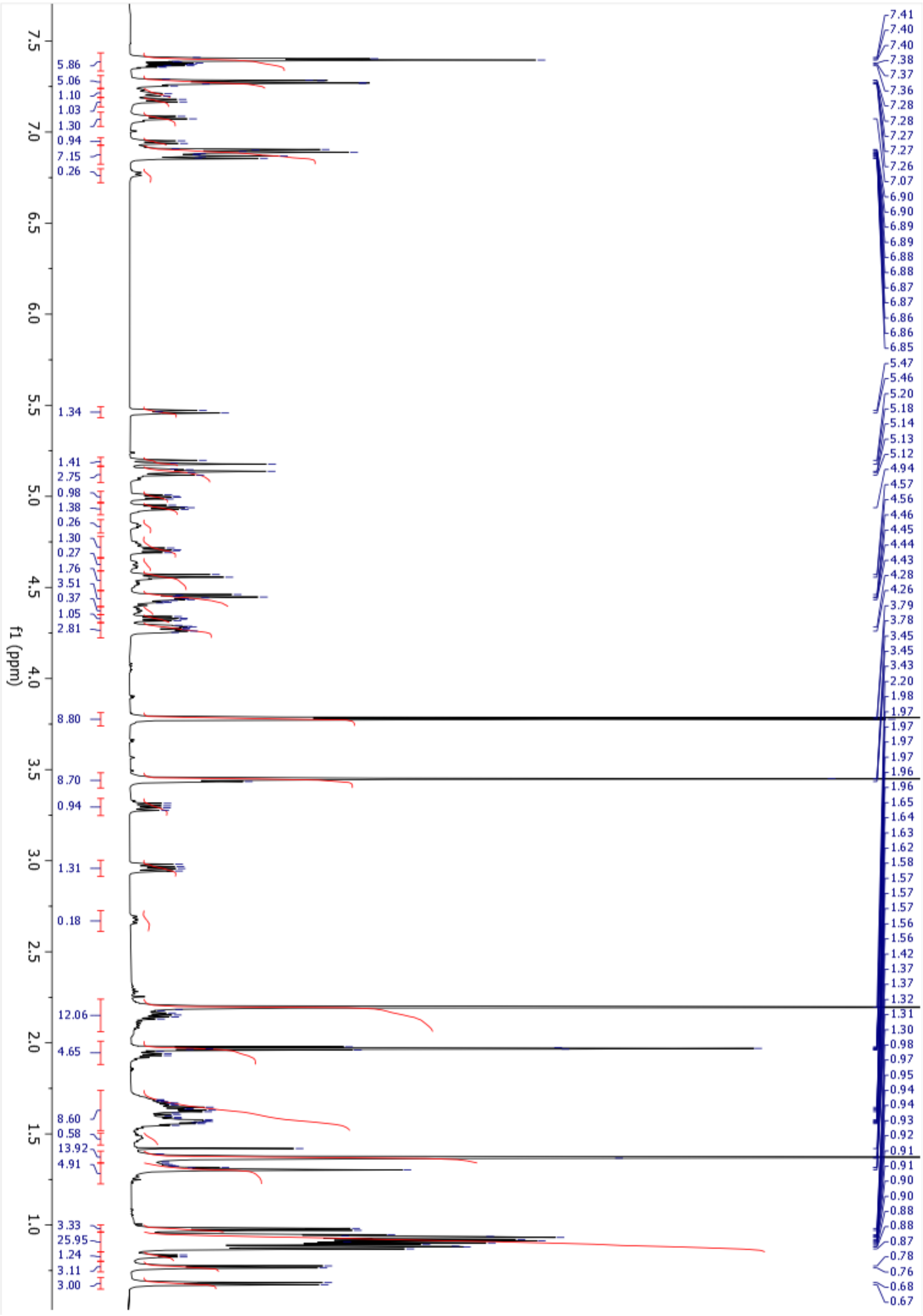

$^{13}C$ NMR (125 MHz, $CD_3CN$)

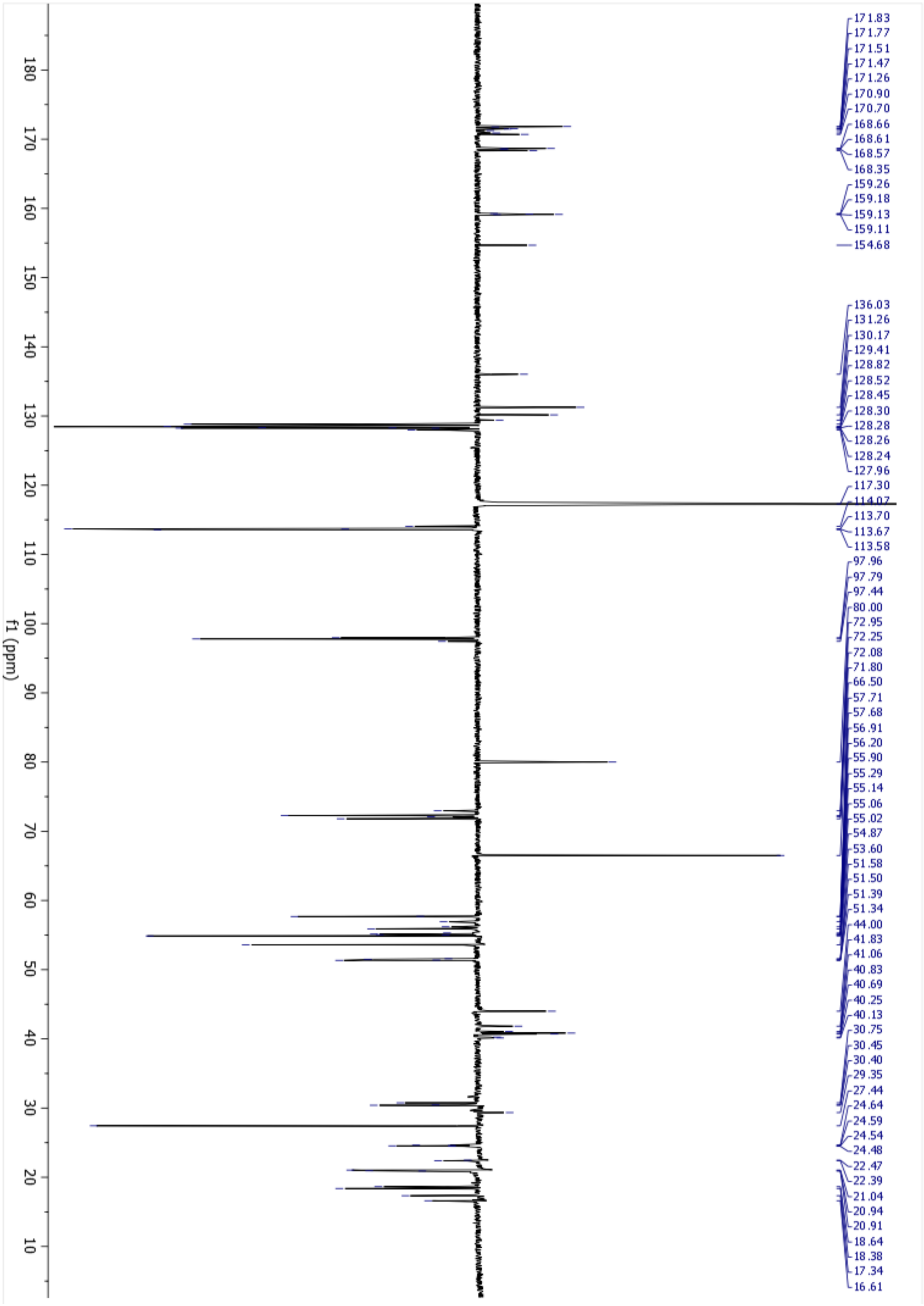

# 4. IR Spectrum of Peptide 4

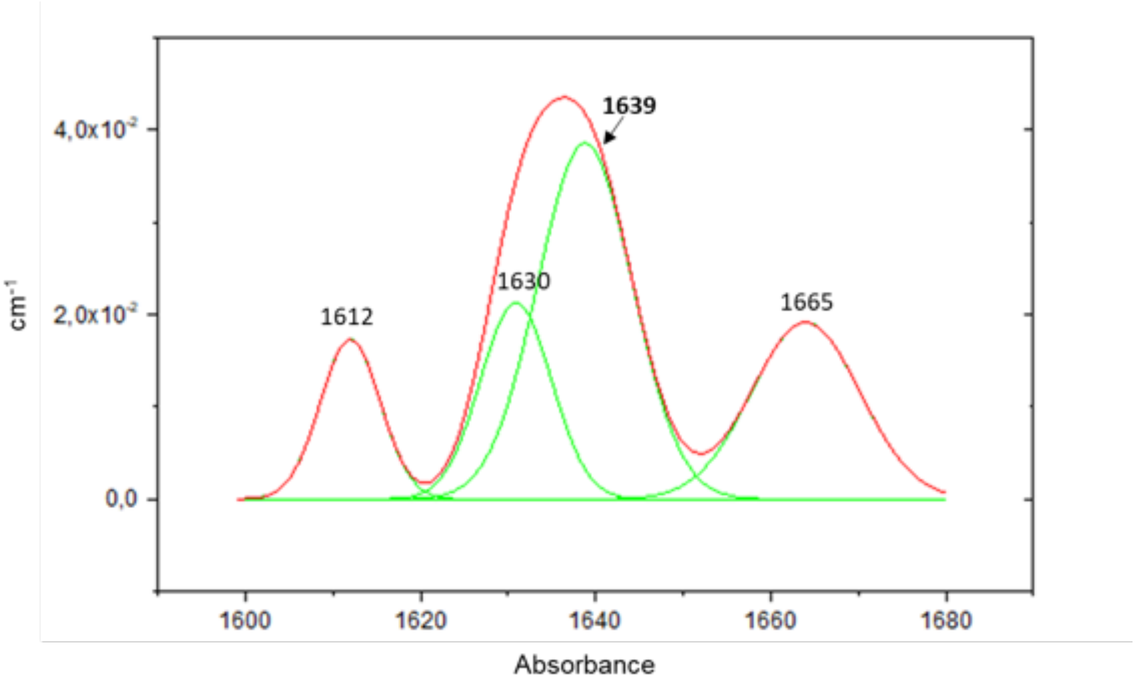


**Figure S8.** FTIR spectra of amide I portion acquired for peptide **4**.

# References


1. Bucci, R., Contini, A., Clerici, F., Pellegrino, S. & Gelmi, M. L. From glucose to enantiopure morpholino β-amino acid: a new tool for stabilizing γ-turns in peptides. *Org. Chem. Front.* **6**, 972–982 (2019).
2. Mahoney, N. M., Janmey, P. A. & Almo, S. C. Structure of the profilin-poly-L-proline complex involved in morphogenesis and cytoskeletal regulation. *Nat. Struct. Biol.* **4**, 953–960 (1997).
3. Maffucci, I. & Contini, A. Improved Computation of Protein–Protein Relative Binding Energies with the Nwat-MMGBSA Method. *J. Chem. Inf. Model.* **56**, 1692–1704 (2016).
4. Dupradeau, F.-Y. *et al.* The R.E.D. tools: advances in RESP and ESP charge derivation and force field library building. *Phys. Chem. Chem. Phys.* **12**, 7821–7839 (2010).
5. Chemical Computing Group ULC. Molecular Operating Environment (MOE), 2019.0102. (2019).
6. Frisch, M. J. *et al. Gaussian 09*. (Gaussian Inc., 2009).
7. Case, D. A. *et al. Amber 2018*. (2018).
8. Maier, J. A. *et al.* ff14SB: Improving the Accuracy of Protein Side Chain and Backbone Parameters from ff99SB. *J. Chem. Theory Comput.* **11**, 3696–3713 (2015).
9. Doshi, U. & Hamelberg, D. Reoptimization of the AMBER Force Field Parameters for Peptide Bond (Omega) Torsions Using Accelerated Molecular Dynamics. *J. Phys. Chem. B* **113**, 16590–16595 (2009).
10. Salomon-Ferrer, R., Götz, A. W., Poole, D., Le Grand, S. & Walker, R. C. Routine Microsecond Molecular Dynamics Simulations with AMBER on GPUs. 2. Explicit Solvent Particle Mesh Ewald. *J. Chem. Theory Comput.* **9**, 3878–3888 (2013).
11. Götz, A. W. *et al.* Routine microsecond molecular dynamics simulations with AMBER on GPUs. 1. generalized born. *J. Chem. Theory Comput.* **8**, 1542–1555 (2012).
12. Maffucci, I. & Contini, A. Improved Computation of Protein-Protein Relative Binding Energies with the Nwat-MMGBSA Method. *J. Chem. Inf. Model.* **56**, 1692–1704 (2016).
